# Crystal structures of *trans*-di­bromido­bis­(4-picoline)gold(III) tetra­bromido­aurate(III) nitro­methane monosolvate, bis­(2-picolinium) tetra­bromido­aurate(III) bromide, and five salts of the type picolinium or lutidinium tetra­halogenidoaurate(III)

**DOI:** 10.1107/S2056989025008801

**Published:** 2025-10-24

**Authors:** Cindy Döring, Peter G. Jones

**Affiliations:** a Institut für Anorganische und Analytische Chemie, Technische Universität Braunschweig, Hagenring 30, D-38106 Braunschweig, Germany; Universität Greifswald, Germany

**Keywords:** crystal structure, tetra­halogenidoaurate(III), hydrogen bond, halogen bond, coinage bond, halogen⋯π contact

## Abstract

2-Picolinium tetra­chlorido­aurate(III), (C_6_H_8_N)[AuCl_4_] or (2-PicH)[AuCl_4_], **1**, and 2-picolinium tetra­bromido­aurate(III), (C_6_H_8_N)[AuBr_4_] or (2-PicH)[AuBr_4_], **2**, both crystallize in the space group *P*1 with *Z* = 4, but are not isotypic. Bis(2-picolinium) tetra­bromido­aurate(III) bromide, (C_6_H_8_N)_2_[AuBr_4_]Br or (2-PicH)_2_[AuBr_4_]Br, **3**, crystallizes in the space group *P*1 with *Z* = 2. All atoms of **1**–**3** lie on general positions. 3-Picolinium tetra­bromido­aurate(III), (C_6_H_8_N)[AuCl_4_] or (3-PicH)[AuBr_4_], **4**, crystallizes in the space group *P*2_1_/*c* with *Z* = 4; the two independent anions each display inversion symmetry. *trans*-Di­bromido­bis­(4-picoline)gold(III) tetra­bromido­aurate(III) nitro­methane mono­sol­vate, [AuBr_2_(C_6_H_7_N)_2_](AuBr_4_]·CH_3_NO_2_ or [(4-Pic)_2_AuBr_2_](AuBr_4_]·CH_3_NO_2_, **5**, and 4-picolinium tetra­bromido­aurate(III), (C_6_H_8_N)[AuBr_4_] or (4-PicH)[AuBr_4_], **6**, both crystallize in the space group *P*1 with *Z* = 2; both involve two independent anions with inversion symmetry. 2,4-Lutidinium tetra­bromido­aurate(III), (C_7_H_10_N)[AuBr_4_] or (2,4-LutH)[AuBr_4_], **7**, crystallizes in the space group *P*2_1_2_1_2_1_ with *Z* = 4. All the gold(III) species show the expected square-planar geometry. The main inter­est centres on the packing patterns. In **1**, hydrogen bonds, Cl⋯Cl contacts, axial Au⋯Cl contacts (‘coinage bonds’) and Cl⋯π contacts combine to form layers parallel to (101). In **2**, similar contacts (but involving Br) link the residues to form corrugated layers parallel to the *ac* plane. In **3**, classical hydrogen bonds, Br⋯Br contacts and a coinage bond, all involving the free bromide ion, combine to produce rings of composition Au_2_Br_4_, which are then linked by another Br⋯Br contact, to form chains of residues parallel to [011]. In **4**, hydrogen bonds and a Br⋯Br contact generate chains of residues parallel to [101], which are in turn linked by a Br⋯π contact. In **5**, Br⋯Br contacts and coinage bonds link the anions and cations to form a corrugated layer structure parallel to the *ac* plane, involving six-membered Au_2_Br_4_ and ten-membered Au_4_Br_6_ rings. A similar combination of contacts in **6** leads to a layer structure parallel to the *ab* plane, also involving a pattern of six- and ten-membered rings topologically analogous to that of **5**. However, the angles in the rings of the two layers differ appreciably, and **6** also contains a short Br⋯π contact. In **7**, a hydrogen bond combines with a coinage bond to produce a ribbon of residues parallel to the *a* axis. Three further, longer and perhaps borderline, Br⋯Br and Br⋯*Cg* contacts link the ribbons to form a three-dimensional pattern.

## Abstract 2

Packing patterns of the title compounds are analysed in terms of hydrogen bonds, halogen bonds, coinage bonds and halogen⋯π contacts.

## 1. Chemical context

In this series of publications, we have structurally investigated several classes of amine complexes of gold(I) and gold(III) halides, whereby the term ‘amine’ has been used loosely to include aza­aromatics; several tetra­halogenidoaurate(III) salts of protonated amines have also been included. The previous part (Part 19; Döring & Jones, 2025*b* ▸) presented some 3,5-lutidine derivatives; general comments given there apply to the current paper as well. Background material was given in Parts 18 and (especially) 12 of this series (Döring & Jones, 2025*a* ▸, 2023 ▸).
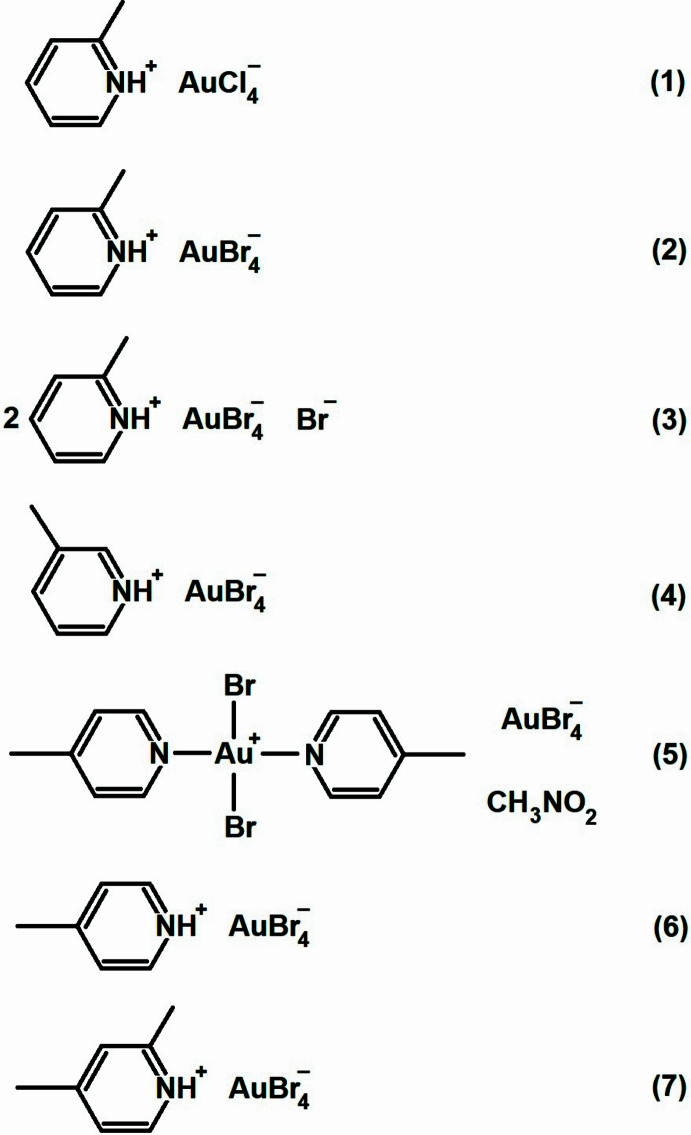


Here we present the structures of the following picoline (methyl­pyridine, abbreviated to Pic) or lutidine (di­methyl­pyridine, abbreviated to Lut) derivatives: 2-picolinium tetra­chlorido­aurate(III), (2-PicH)[AuCl_4_], **1** and tetrabromidoaurate(III), (2-PicH)[AuBr_4_], **2**; bis­(2-picolinium) tetra­bromido­aurate(III) bromide, (2-PicH)_2_[AuBr_4_]Br, **3**; 3-picolinium tetra­bromido­aurate(III), (3-PicH)[AuBr_4_], **4**; *trans*-di­bromido­bis­(4-picoline)gold(III) tetra­bromido­aurate(III) nitro­methane mono­solvate, [(4-Pic)_2_AuBr_2_](AuBr_4_](CH_3_NO_2_), **5**; 4-picolinium tetra­bromido­aurate(III), (4-PicH)[AuBr_4_], **6** and 2,4-lutidinium tetra­bromido­aurate(III), (2,4-LutH)[AuBr_4_], **7.**

## 2. Structural commentary

**Figure 1:**
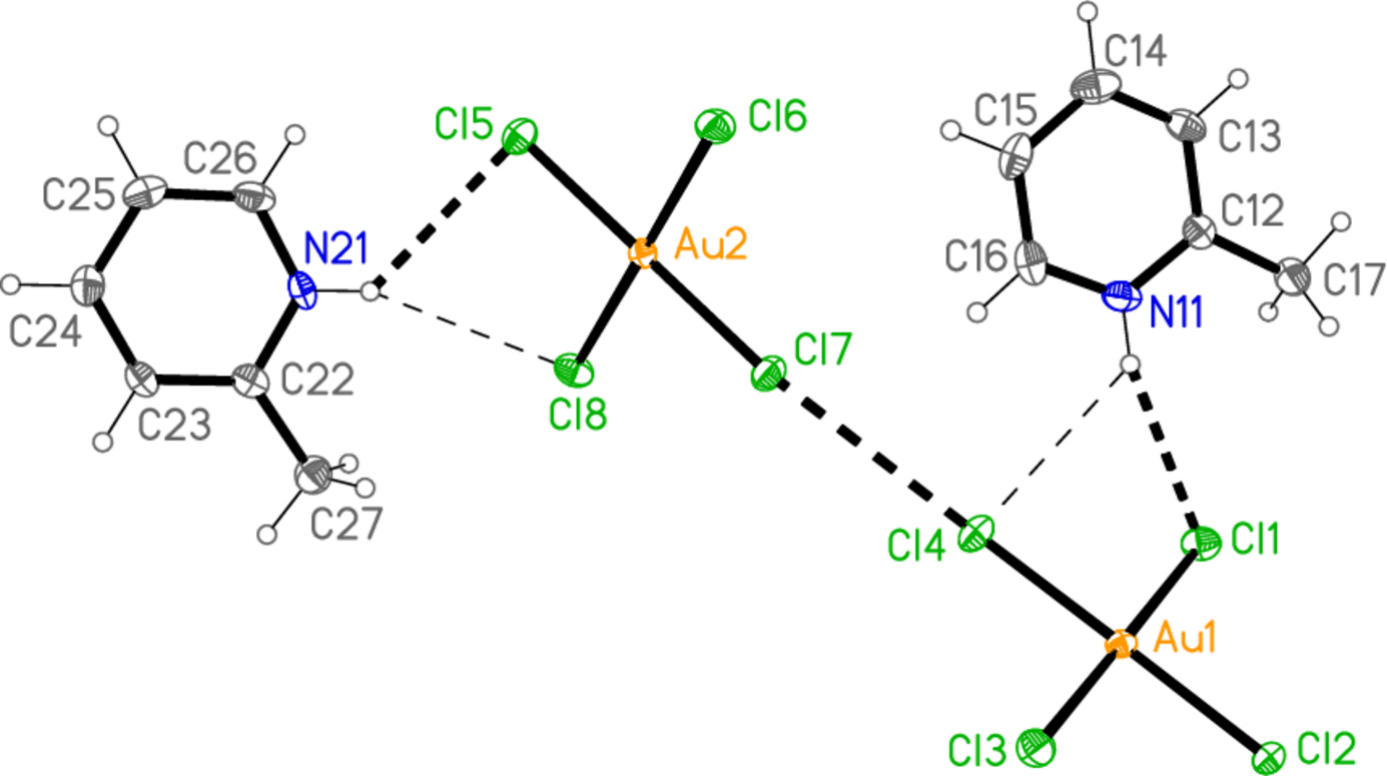
The asymmetric unit of compound **1** in the crystal. Ellipsoids are drawn at the 50% level for all structures. Dashed lines indicate Cl⋯Cl contacts or the shorter components of three-centre hydrogen bonds (thick) or the longer such components (thin).

All compounds except the nitro­methane solvate **5** crystallize solvent-free. In the Figures (Figs. 1 ▸–7 ▸ ▸ ▸ ▸ ▸ ▸), the asymmetric units have been extended by symmetry where necessary to show complete residues; the dashed lines indicate short contacts, which are discussed in *Supra­molecular features*. All ellipsoids are drawn at the 50% level. Selected mol­ecular dimensions are shown in Tables 1 ▸–7 ▸ ▸ ▸ ▸ ▸ ▸.

Compounds **1** and **2** both crystallize in *P*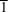 with *Z* = 4 but are not isotypic. Compound **3** crystallizes in *P*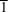 with *Z* = 2. All atoms of **1**–**3** lie on general positions. Compound **4** crystallizes in *P*2_1_/*c* with *Z* = 4; there are two independent anions, each with inversion symmetry. Compounds **5** and **6** crystallize in *P*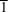 with *Z* = 2; both involve two independent anions, each with inversion symmetry. Compound **7** crystallizes in *P*2_1_2_1_2_1_ with *Z* = 4.

All the gold(III) species show the expected square-planar geometry. The tetra­halogenidoaurate(III) anions are close to the expected 4/*mmm* local symmetry, whereby the Au—Br bond lengths lie in the range 2.4130 (12)–2.4340 (6) Å and the largest deviations from 90 and 180° angles are 1.1 and 4.5°, respectively. In the cation of compound **5**, the Au—N and Au—Br bond lengths are, as expected, similar to those of the *trans*-[(3,5-Lut)_2_AuBr_2_] cation in its tribromide salt [Au—N 2.025 (2) and Au—Br 2.4174 (3) in the first and Au—N 2.020 (4), 2.032 (4), Au—Br 2.4090 (4) Å in the second polymorph; Döring & Jones, 2025*b* ▸]. The angles between the gold(III) coordination plane and the picoline ring plane of **5** are 56.4 (2)° in the first cation and 58.3 (2)° in the second. The C—N—C angles of the lutidine ligands in **5** are close to 120°, whereas the corresponding angles of the picolinium and lutidinium cations in **1**–**4**, **6** and **7** lie in the range 122.9 (7)–124.9 (11)°.

## 3. Supra­molecular features

In the packing diagrams, atom labels indicate atoms of the asymmetric unit. Hydrogen atoms of the ring CH groups are omitted; we subjectively assess the C—H⋯halogen contacts to be less important than N—H⋯halogen (except perhaps for the sole chloride derivative **1**; see below). Clearly, there is an implicit contradiction in the description of packing in terms of a few selected contacts and the fact that the packing energies almost certainly involve significant contributions from a much larger number of van der Waals contacts such as H⋯H [*cf*. the comments of Dance (2003 ▸)]. In the text, primes (′) indicate previously defined or generalized symmetry operators. Hydrogen bonds are listed in Tables 7 ▸–14 ▸ ▸ ▸ ▸ ▸ ▸ ▸. The rings are numbered with respect to the first digit of the nitro­gen atom numbers; thus ring 2 is based on the nitro­gen atom N21. The abbreviation ‘*Cgn*’ refers to the centre of gravity of the ring *n*. For many contact types, there was no clear cutoff distance for a ‘significant’ contact/inter­action, and some borderline cases were arbitrarily omitted for clarity; some of these are commented on explicitly below.

Our recent investigations have analysed packing patterns in terms of secondary inter­actions such as hydrogen bonds, halogen bonds [for reviews see *e.g.* Cavallo *et al.* (2016 ▸) or Metrangolo *et al.* (2008 ▸)] or coinage bonds [a recent formalization, in terms of π holes at the gold atom, of the axial contacts to square-planar gold(III) centres; Daolio *et al.* (2021 ▸) and Pizzi *et al.*, 2022 ▸)]. Less common features (Döring & Jones, 2025*b* ▸) are the mixed stacking of aromatic rings and tetra­halogenidoaurate ions, and contacts of the type halogen⋯π (which may be regarded as a special form of halogen bond).

The asymmetric unit of compound **1** (Fig. 1 ▸) was chosen to include the two asymmetric three-centre hydrogen-bond systems of the type N—H⋯(Cl,Cl), together with the short contact Cl4⋯Cl7 [3.3947 (13) Å, with angles Au1—Cl4⋯Cl7 = 157.89 (5) and Au2—Cl7⋯Cl4 = 154.42 (4)°]. Another such contact is Cl2⋯Cl5(−1 + *x*, *y*, 1 + *z*) [3.4586 (14) Å, with Au1—Cl2⋯Cl5’ = 153.16 (4) and Au2—Cl5⋯Cl2′ = 152.76 (5)°]. These combine with the short axial contacts (coinage bonds) Au1⋯Cl6(1 − *x*, −*y*, 1 − *z*) = 3.5947 (10) and Au2⋯Cl3(1 − *x*, 1 − *y*, 1 − *z*) = 3.3963 (10) Å to form a layer structure parallel to (101) (Fig. 8 ▸). The Cl⋯Cl linkages run horizontally in Fig. 8 ▸, parallel to [10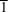], whereas the coinage bonds link the anions vertically (parallel to the *b* axis). The structure also involves a considerable number of ‘weak’ C—H⋯Cl hydrogen bonds, notably the three-centre system H16⋯(Cl4, Cl7) and one component of the double-acceptor system (H02, H26)⋯Cl5 within the asymmetric unit, but these are not included in Fig. 1 ▸ or Fig. 8 ▸.

The chlorine atoms Cl2 and Cl7 are also involved in the short Cl⋯π contacts Cl2⋯*Cg*2(1 − *x*, 1 − *y*, 1 − *z*) = 3.398 (2) and Cl7⋯*Cg*1(1 − *x*, −*y*, 1 − *z*) = 3.399 (2) Å, with Au—Cl⋯*Cg* angles of 121.3 and 117.5°, respectively. Fig. 9 ▸ shows the layer of Fig. 8 ▸ viewed from the side (parallel to the *b* axis), showing the appreciable thickness of the layers and the linking role of the Cl⋯π contacts. The significantly longer contacts Cl1⋯*Cg*1(−*x*, −*y*, 1 − *z*) = 3.673 (2) and Cl5⋯*Cg*2(1 − *x*, 1 − *y*, −*z*) = 3.695 (2) Å may play a minor structural role in linking the layers, but have been omitted from the packing diagrams.

**Figure 2:**
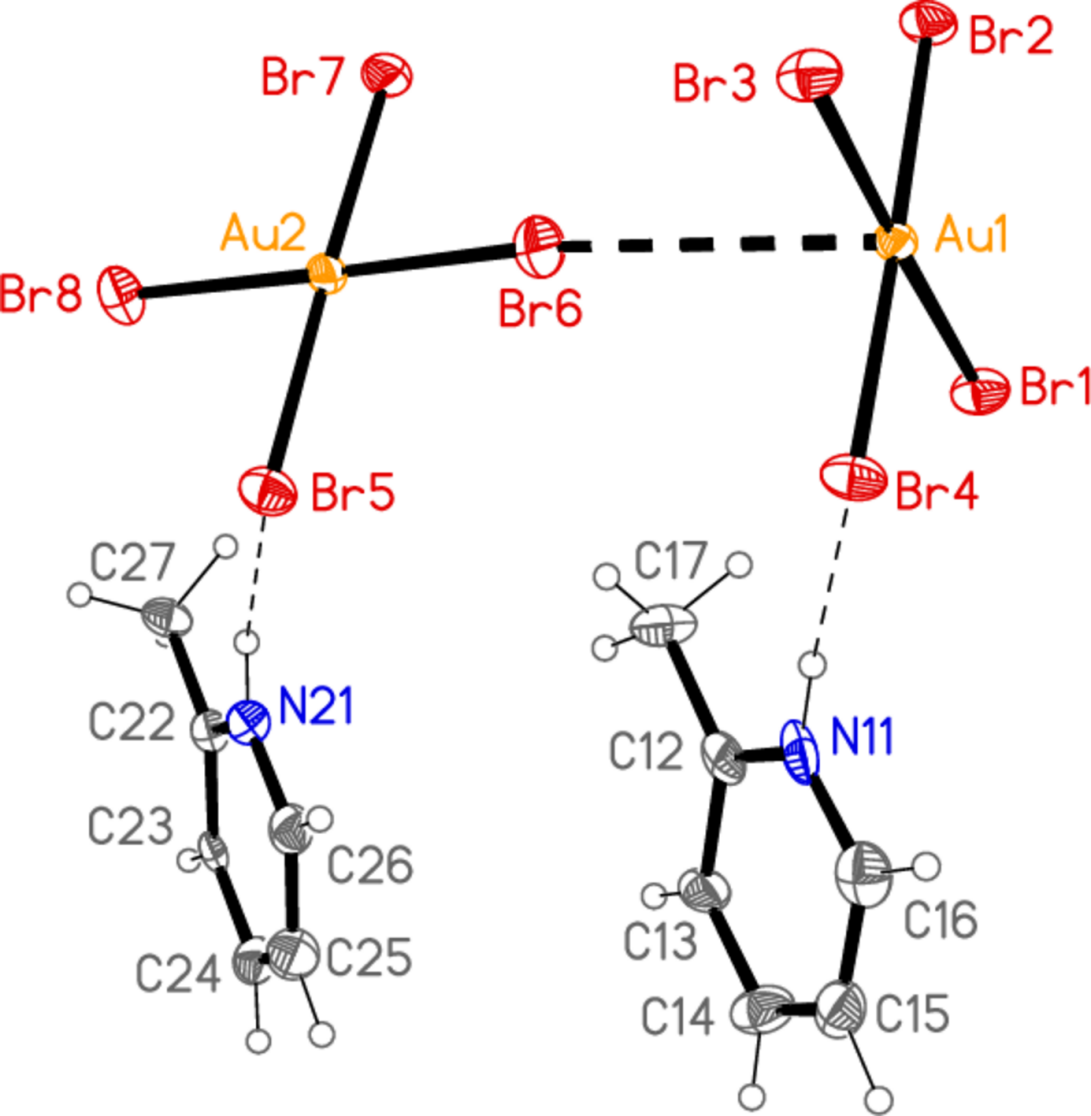
The asymmetric unit of compound **2** in the crystal. Dashed lines indicate an Au⋯Br contact (thick) or hydrogen bonds (thin).

The asymmetric unit of compound **2** (Fig. 2 ▸) was chosen to contain the two classical hydrogen bonds of the type N—H⋯Br and the coinage bond Au1⋯Br6 [3.6926 (13) Å]. There are also two further such contacts [Au1⋯Br8(*x*, *y*, 1 + *z*) = 3.7660 (12) and Au2⋯Br1(1 − *x*, 1 − *y*, 1 − *z*) = 3.5899 (11) Å] and two short bromine-bromine contacts [Br4⋯Br4(−*x*, 1 − *y*, 2 − *z*) = 3.576 (2) Å, with Au1—Br4⋯Br4′ = 146.85 (6)°, and Br5⋯Br5(−*x*, 1 − *y*, 1 − *z*) = 3.622 (2) Å, with Au2—Br5⋯Br5’ = 144.27 (6)°]. The two coinage bonds at Au1 link the anions to form chains parallel to the *c* axis, and these chains are cross-linked by the remaining contacts to form a layer structure parallel to the *ac* plane (Fig. 10 ▸). As for **1**, the layer also contains halogen⋯π contacts, namely Br7⋯*Cg*1(1 − *x*, 1 − *y*, 1 − *z*) = 3.499 (4) and Br2⋯*Cg*2(1 − *x*, 1 − *y*, 1 − *z*) = 3.468 (6) Å (with Au—Br⋯*Cg* angles of 126.9 and 119.6°, respectively) and the somewhat longer Br5⋯*Cg*1(-x, 1 − y, 1 − z) = 3.703 (4) and Br4⋯*Cg*2(-x, 1 − y, 1 − z) = 3.764 (5) Å, all within the layer. To avoid overloading the packing diagram, just one of each contact (those involving the rings of the asymmetric unit) has been included explicitly. A projection of the structure parallel to the *c* axis (omitting Br⋯π contacts; Fig. 11 ▸) shows the corrugated nature of the layers. The contacts Br2⋯Br2(1 − *x*, 2 − *y*, 1 − *z*) = 3.813 (2) and Br7⋯Br7(1 − *x*, 2 − *y*, −*z*) 3.872 (2) Å between the layers may be too long to be significant.

**Figure 3:**
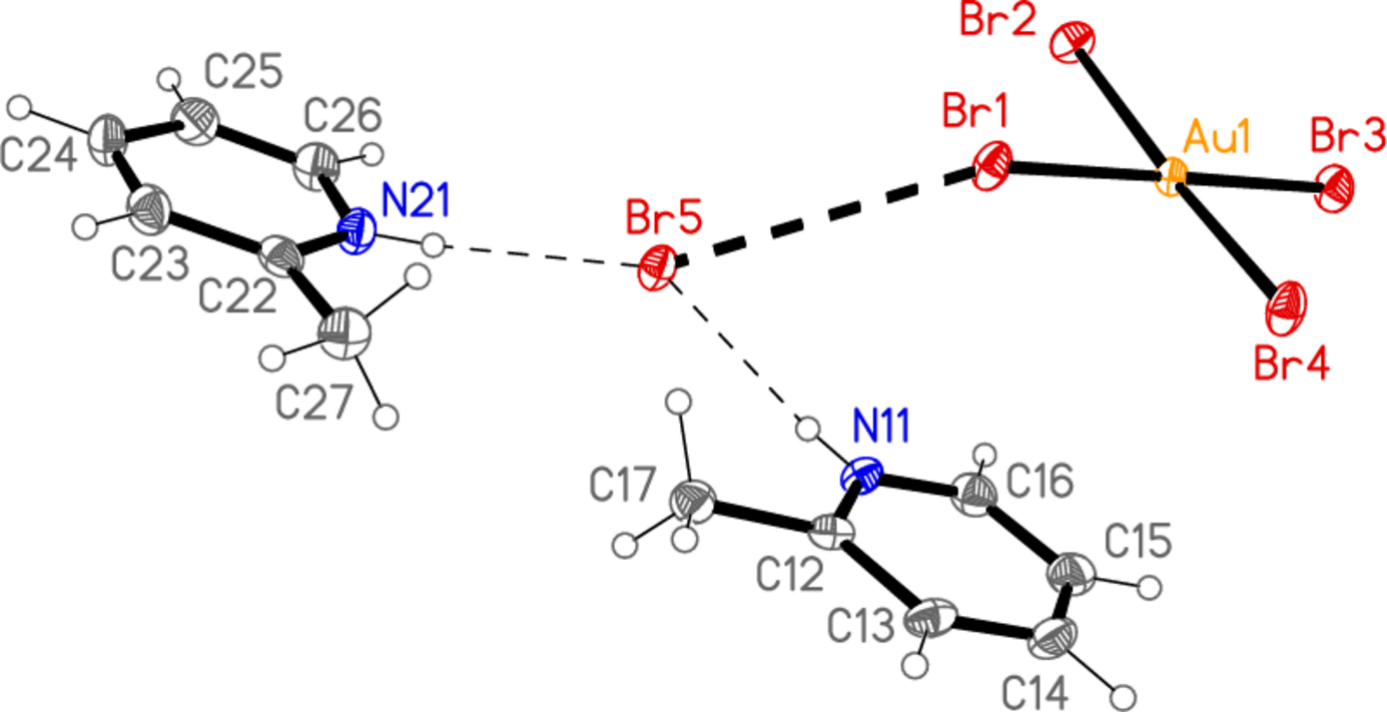
The asymmetric unit of compound **3** in the crystal. Dashed lines indicate a Br⋯Br contact (thick) or hydrogen bonds (thin).

The asymmetric unit of compound **3** (Fig. 3 ▸) contains two classical N—H⋯Br hydrogen bonds and the contact Br1⋯Br5 [3.7399 (6) Å, with Au1—Br1⋯Br5 = 150.30 (2)°]; the free bromide ion Br5 is involved in all three of these inter­actions. The coinage bond Au1⋯Br5(−*x*, 2 − *y*, 1 − *z*) = 3.7451 (5) Å] then leads to rings of composition Au_2_Br_4_, which are further linked by the contact Br3⋯Br3(−*x*, 3 − *y*, −*z*) = 3.5948 (8) Å, with Au1—Br3⋯Br3′ = 153.53 (2)°, to form chains of residues parallel to [01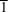] (Fig. 12 ▸).

**Figure 4:**
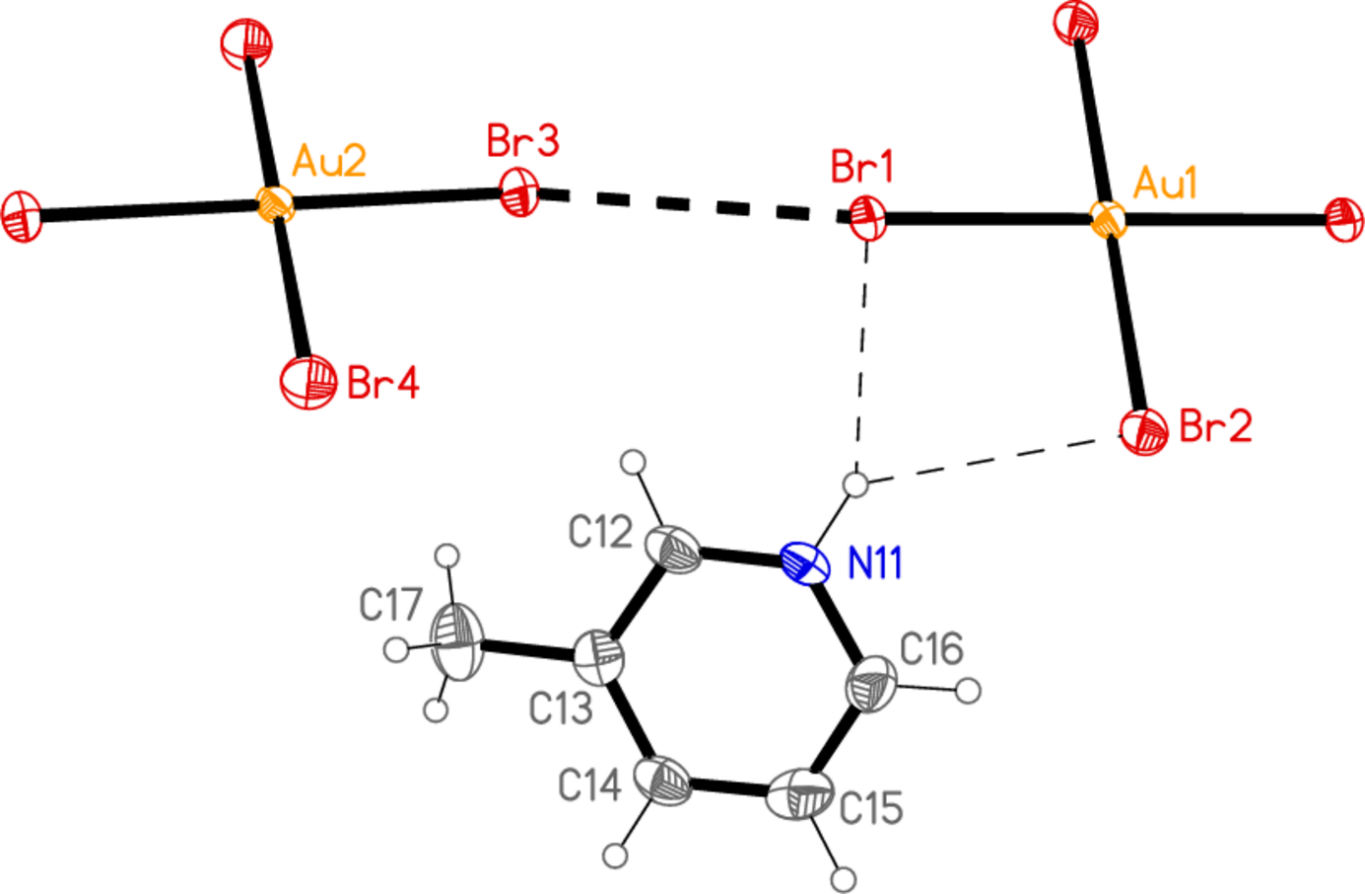
The asymmetric unit of compound **4** in the crystal, extended by symmetry to form complete anions. Dashed lines indicate a Br⋯Br contact (thick) or hydrogen bonds (thin).

The asymmetric unit of compound **4** (extended by symmetry to generate complete ions; Fig. 4 ▸) contains the three-centre hydrogen bond N11—H01⋯(Br1, Br2) and the contact Br1⋯Br3 [3.4957 (8) Å, with Au1—Br1⋯Br3 = 162.53 (3) and Au2—Br3⋯Br1 = 155.20 (3)°]. Together with the two inversion operators corresponding to the special positions of the gold atoms, this generates a chain of residues parallel to [101] in the region *y* ≃ 0. Three such chains are shown in Fig. 13 ▸. The linear moieties Br2—Au1—Br2′ and Br4—Au2—Br4′ are inclined to (10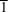) in the opposite sense for the central chain compared to the other two chains. Adjacent chains are linked by the Br⋯π contact Br1⋯*Cg*(1 − *x*, −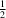 + *y*, 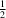 − *z*) = 3.528 (2) Å, with Au—Br⋯*Cg* = 111.5°. The packing is further complicated by a series of borderline contacts: the coinage bonds Au1⋯Br4′ = 3.8290 (6) and Au2⋯Br2′ = 3.8282 (6) Å (operator 1 − *x*, −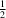 + *y*, 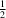 − *z*) and the contact Br2⋯Br4(*x*, 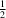 − *y*, 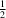 + *z*) = 3.8374 (9) Å, which connect the anions to form a three-dimensional network. There is also a somewhat longer Br⋯π contact, namely Br3⋯*Cg*(−*x*, −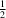 + *y*, 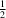 − *z*) = 3.767 (2) Å.

**Figure 5:**
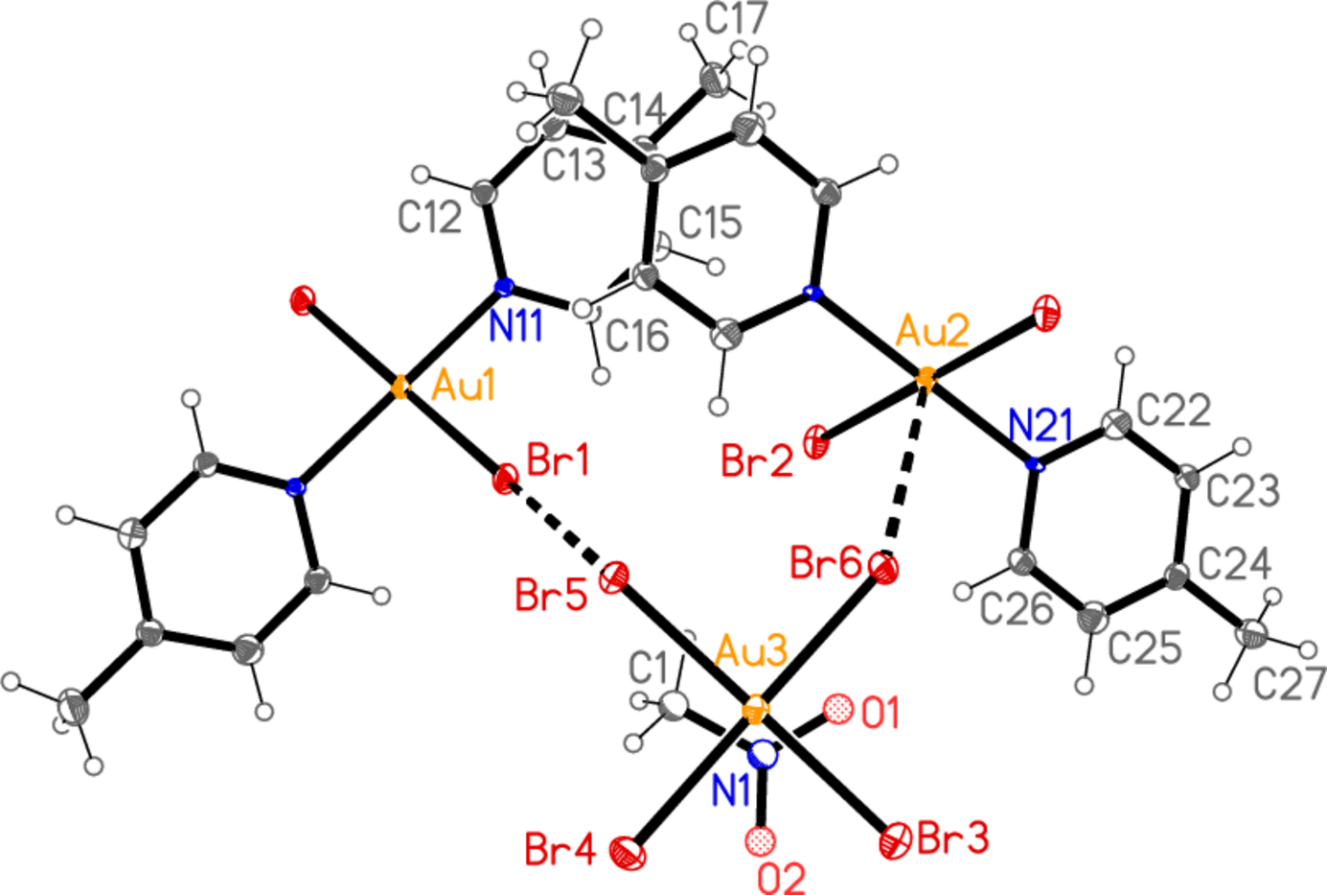
The asymmetric unit of compound **5** in the crystal, extended by symmetry to form complete cations. Dashed lines indicate Au⋯Br or Br⋯Br contacts. The solvent mol­ecule is drawn as spherical atoms of arbitrary radius.

The asymmetric unit of compound **5** consists of two half cations and one anion (Fig. 5 ▸), and includes the short contact Br1⋯Br5 [3.5058 (14) Å, with Au1—Br1⋯Br5 = 158.16 (5) and Au3—Br5⋯Br1 = 125.72 (4)°] and the coinage bond Au2⋯Br6 = 3.3709 (11) Å. Further contacts Br2⋯Br6(−1 + *x*, *y*, *z*) = 3.5407 (15) Å [with Au2—Br2⋯Br6′ = 166.48 (5) and Br2⋯Br6′—Au3′ = 106.50 (4)°] link the anions and cations to form a corrugated layer structure (Fig. 14 ▸) parallel to the *ac* plane, involving six-membered Au_2_Br_4_ rings (with two Au—Br bonds from cations, two Au⋯Br and two Br⋯Br contacts) and ten-membered Au_4_Br_6_ rings (with four Au—Br bonds from anions and two from cations, two Au⋯Br and two Br⋯Br contacts). The atoms Br5 and Br6 take part in both types of ring, and each has two short contacts (Au⋯Br and Br⋯Br), thus attaining an approximately trigonal–planar geometry; *cf*. the unusually narrow Br⋯Br—Au angles at these atoms (see above), which differ greatly from the usual approximately linear values. A closely analogous pattern was observed for the triclinic polymorph of the related compound *trans*-di­bromido­bis­(3,5-lutidine)gold(III) tribromide (Döring & Jones, 2025*b* ▸). A projection of the structure of **5** parallel to the *a* axis (Fig. 15 ▸) shows the corrugation, with the anions constituting the fold regions. In view of the poorly resolved nature of the solvent mol­ecule, we do not comment on it in detail, except to point out that its oxygen atoms accept two short hydrogen bonds from CH donors.

The asymmetric unit of compound **6** contains the contact Br2⋯Br3 [3.5839 (9) Å, with Au1—Br2⋯Br3 very narrow at 82.96 (2) and Au2—Br3⋯Br2 = 164.03 (3)°], together with the classical hydrogen bond N11—H01⋯Br3. The hydrogen bonding might be regarded as a three-centre system including a longer branch H01⋯Br3; however, the position of H01 is not well-determined, with s.u.’s of *ca*. 0.1 Å for the H⋯Br distances. In combination with the coinage bond Au2⋯Br2(1 − *x*, 1 − *y*, 2 − *z*), 3.3777 (6) Å, a layer structure parallel to the *ab* plane is formed (Fig. 16 ▸), which consists of six- and ten-membered rings forming a pattern topologically analogous to that of **5**, despite the major chemical differences between **5** and **6** (*e.g.* the presence of coordinated or protonated pyridine rings). However, the angles in the rings of the two layers differ appreciably; particularly notable in **6** are the angles Au1—Br2⋯Br3 and the nearly linear Au1—Br2⋯Au2′ [162.50 (2)°]. There are also two Br⋯π contacts, namely Br4⋯*Cg*(1 + *x*, *y*, *z*) = 3.694 (3) Å, with Au2—Br4⋯*Cg*′ = 113.1°, and the perhaps borderline Br1⋯*Cg*(1 − *x*, 1 − *y*, 1 − *z*) = 3.8240 (3) Å, with Au1—Br1⋯*Cg*′ = 126.7°. These lie within the layers but are not drawn in Fig. 16 ▸ because they are almost parallel to the view direction. Fig. 17 ▸ shows these contacts clearly, together with the rather long Br1⋯Br4(1 − *x*, 1 − *y*, 1 − *z*) contact of 3.7167 (10) Å, with Au1—Br1⋯Br4′ = 155.27 (3) and Au2—Br4⋯Br1′ = 164.43 (3)°, which links the layers at *z* ≃ 0 and 1.

**Figure 7:**
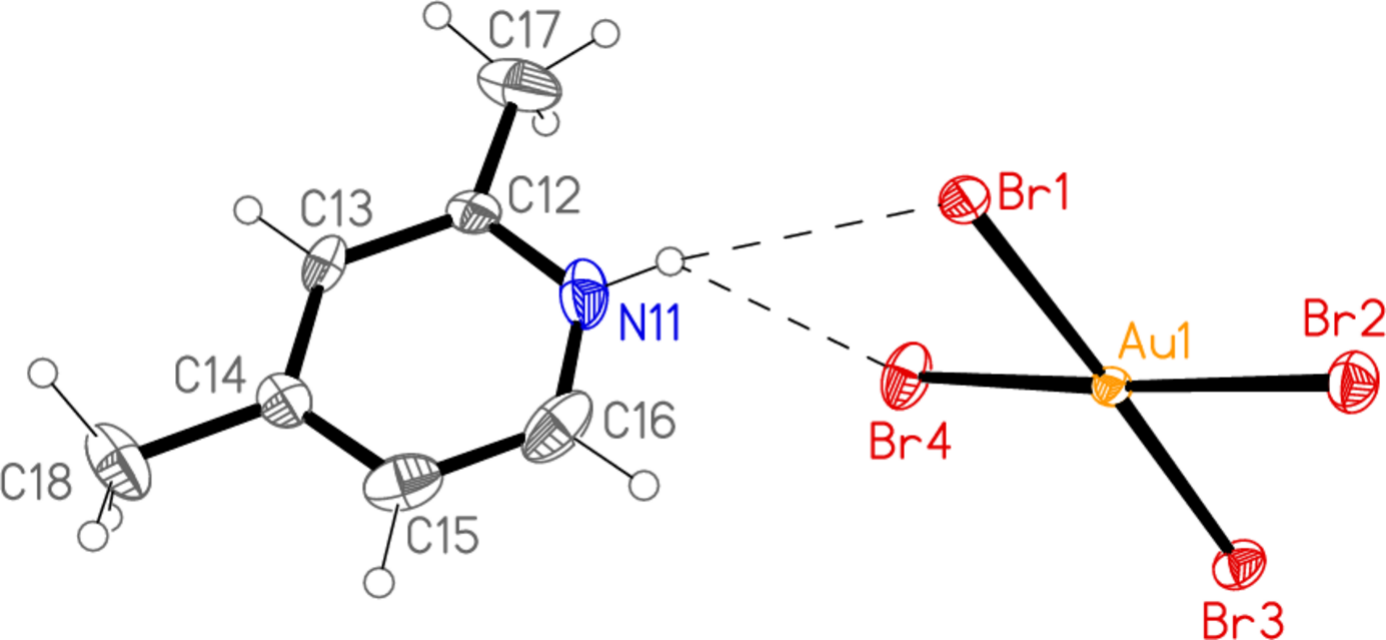
The asymmetric unit of compound **7** in the crystal. Dashed lines indicate a three-centre hydrogen-bond system.

**Figure 8:**
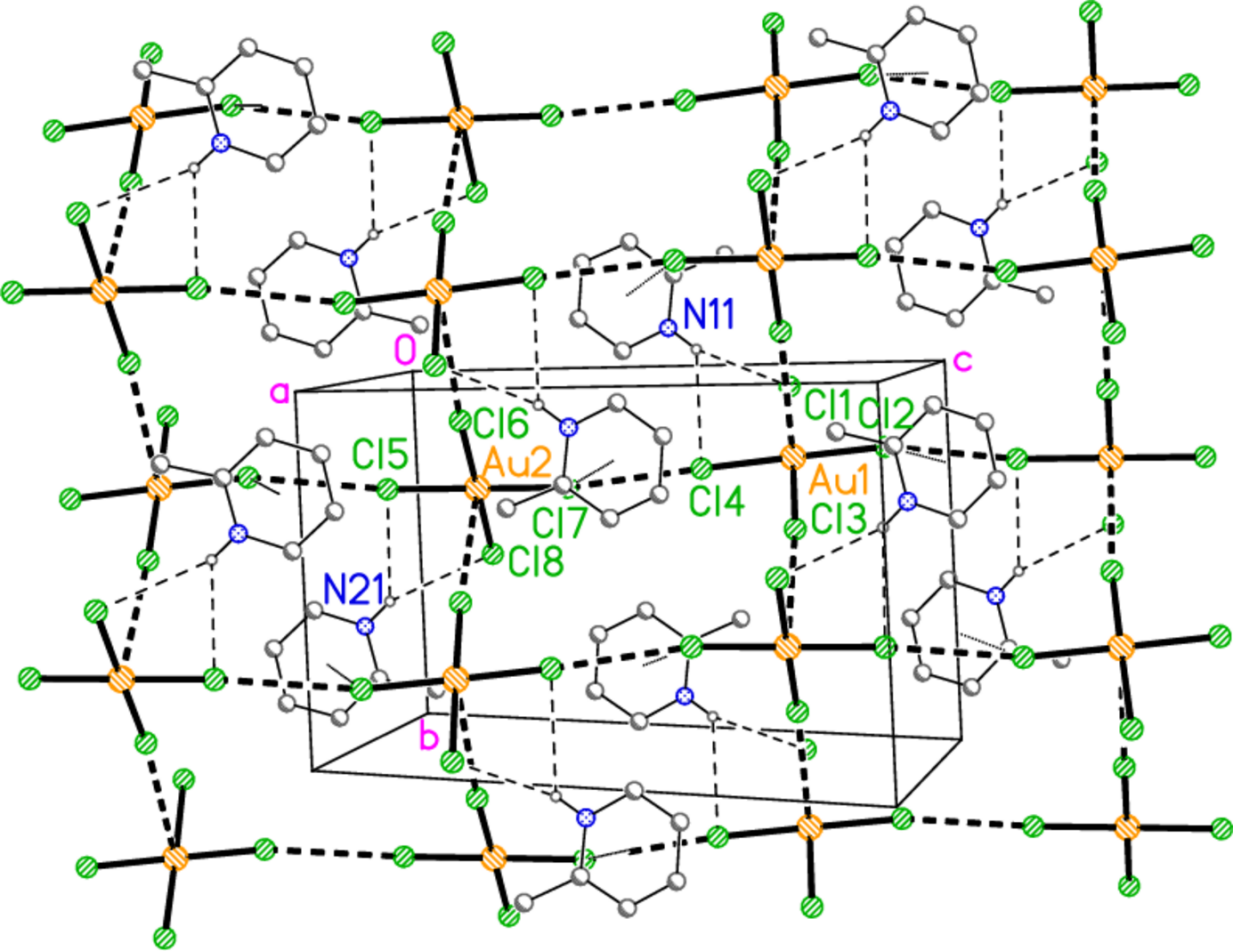
Packing diagram of compound **1**, viewed perpendicular to (101). Dashed lines indicate Cl⋯Cl or Au⋯Cl contacts (thick) or hydrogen bonds (thin). The Cl⋯π contacts are shown as faint dotted lines (although some are obscured in this view direction, as are the labelled atoms Cl2 and Cl7).

**Figure 9:**
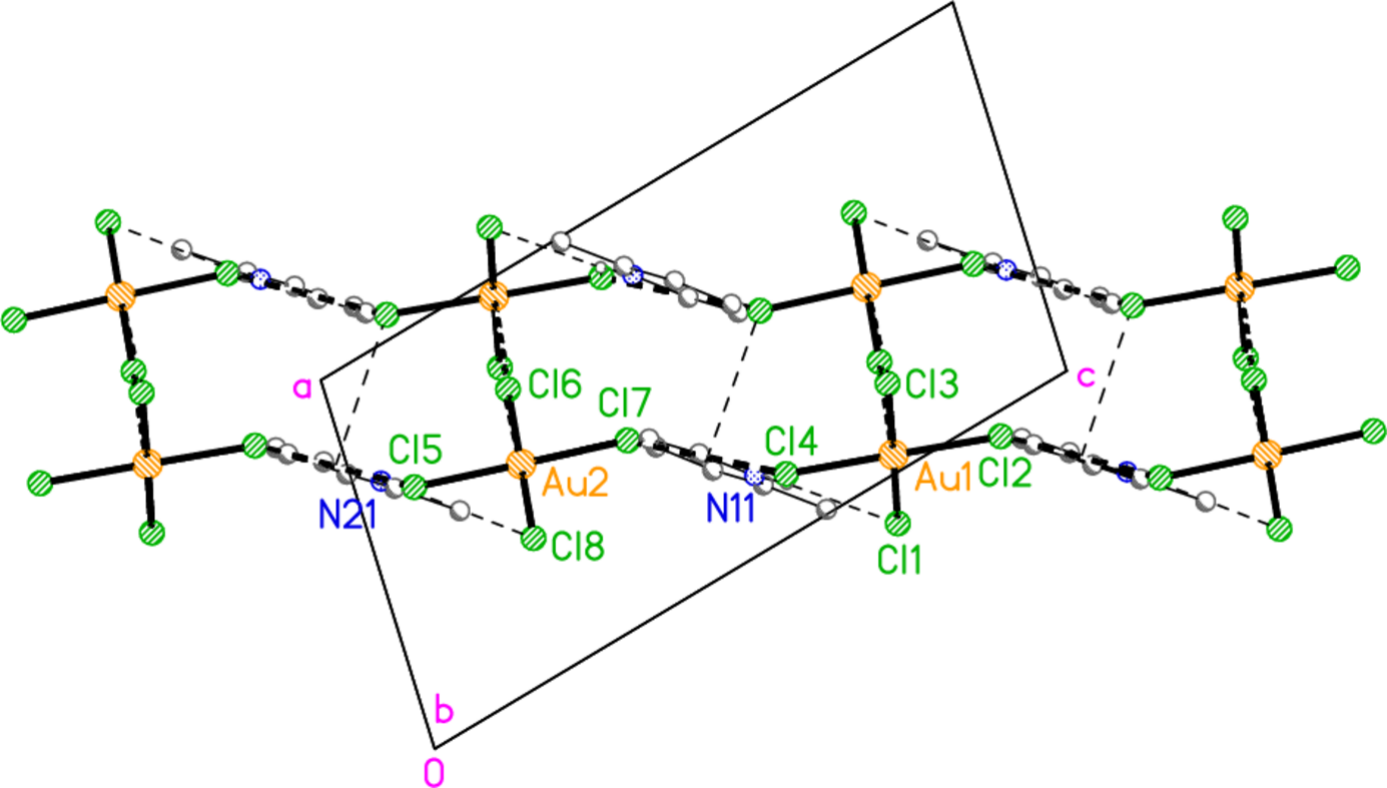
The packing of compound **1** projected parallel to the *b* axis (same atoms as in Fig. 8 ▸), showing the linking role of the Cl⋯π inter­actions.

**Figure 10:**
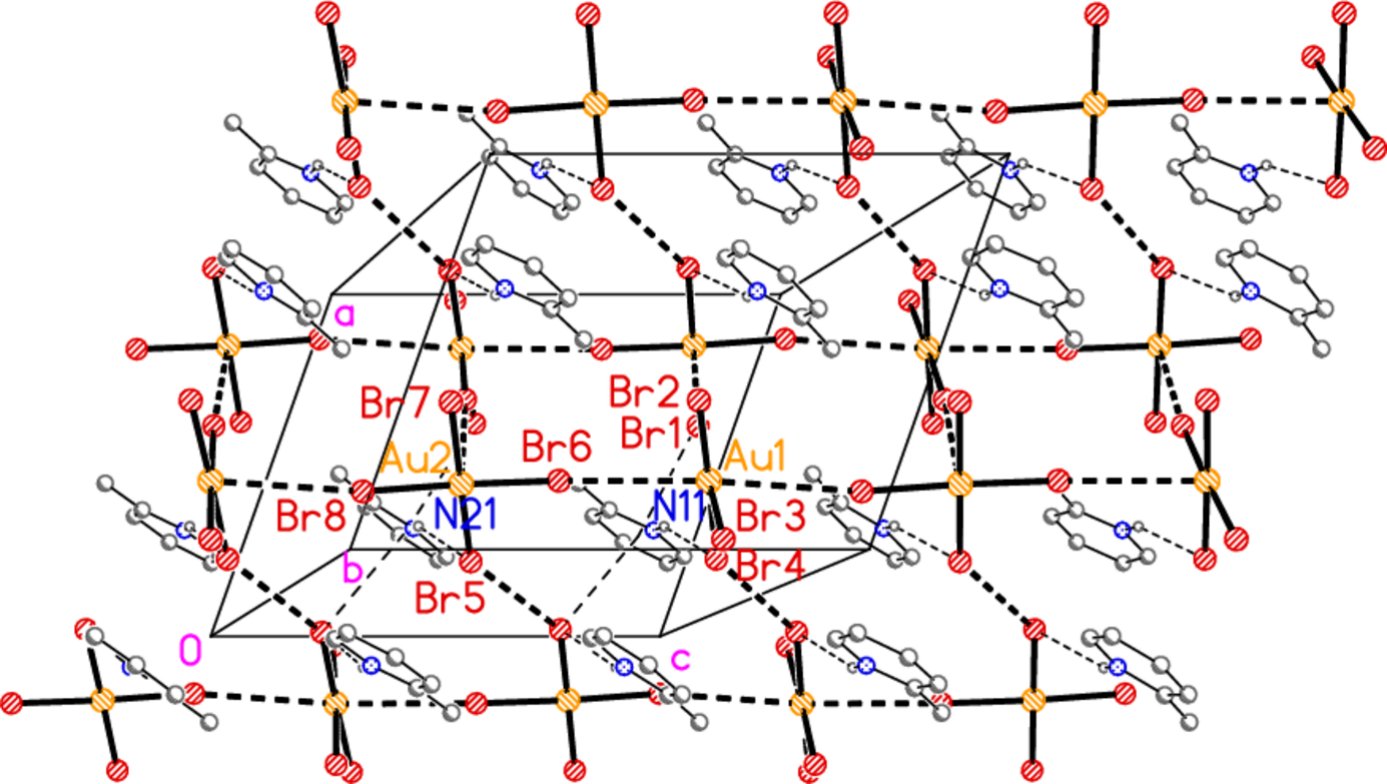
The packing of compound **2**, viewed perpendicular to the *ac* plane. Dashed lines indicate Br⋯Br or Au⋯Br contacts (thick) or hydrogen bonds (thin). Four representative Br⋯π inter­actions (involving the parent rings at N11 and N21) have also been included as thin dashed lines.

**Figure 11:**
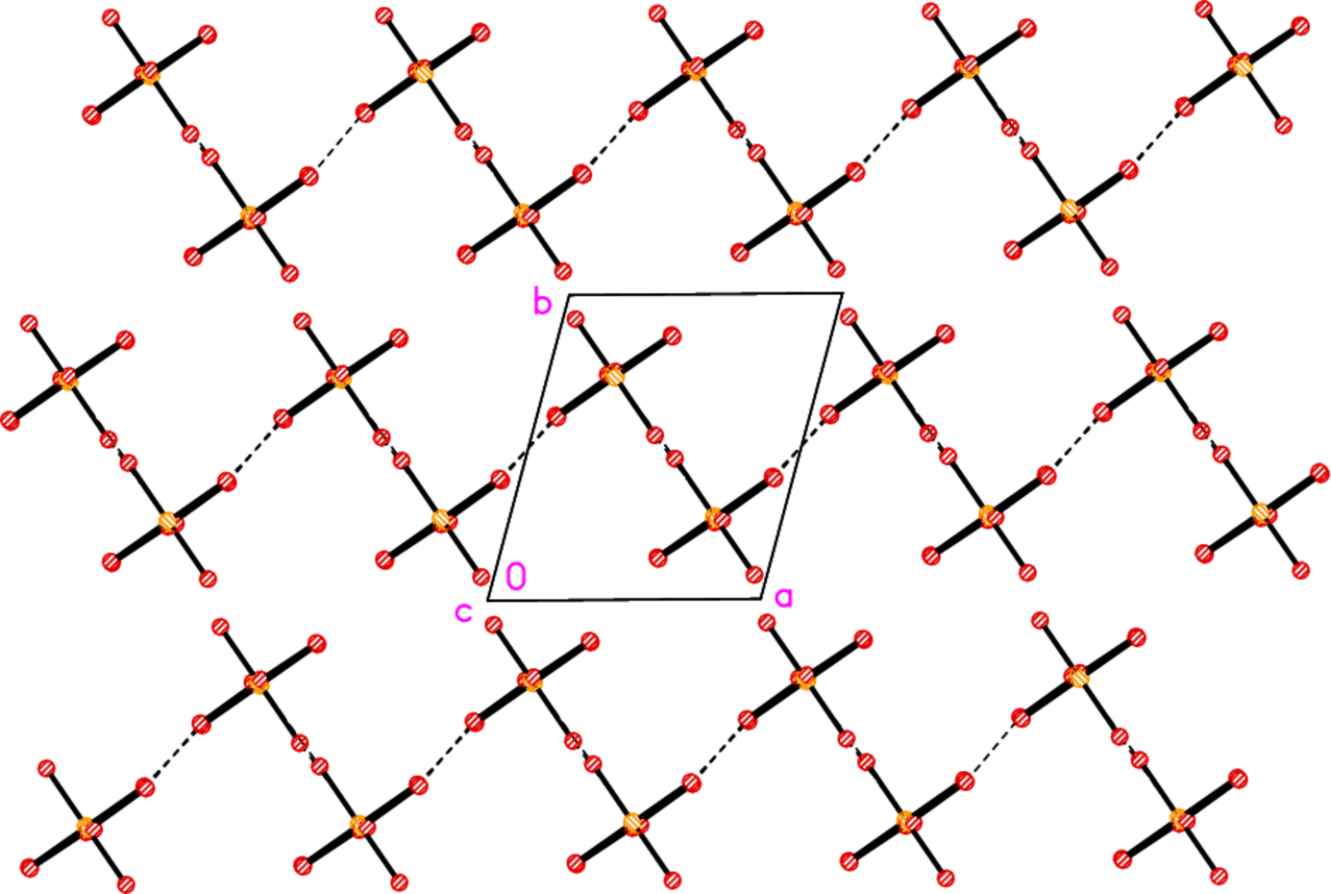
A layer of compound **2**, projected parallel to the *c* axis. Cations and Br⋯π inter­actions are omitted.

**Figure 12:**
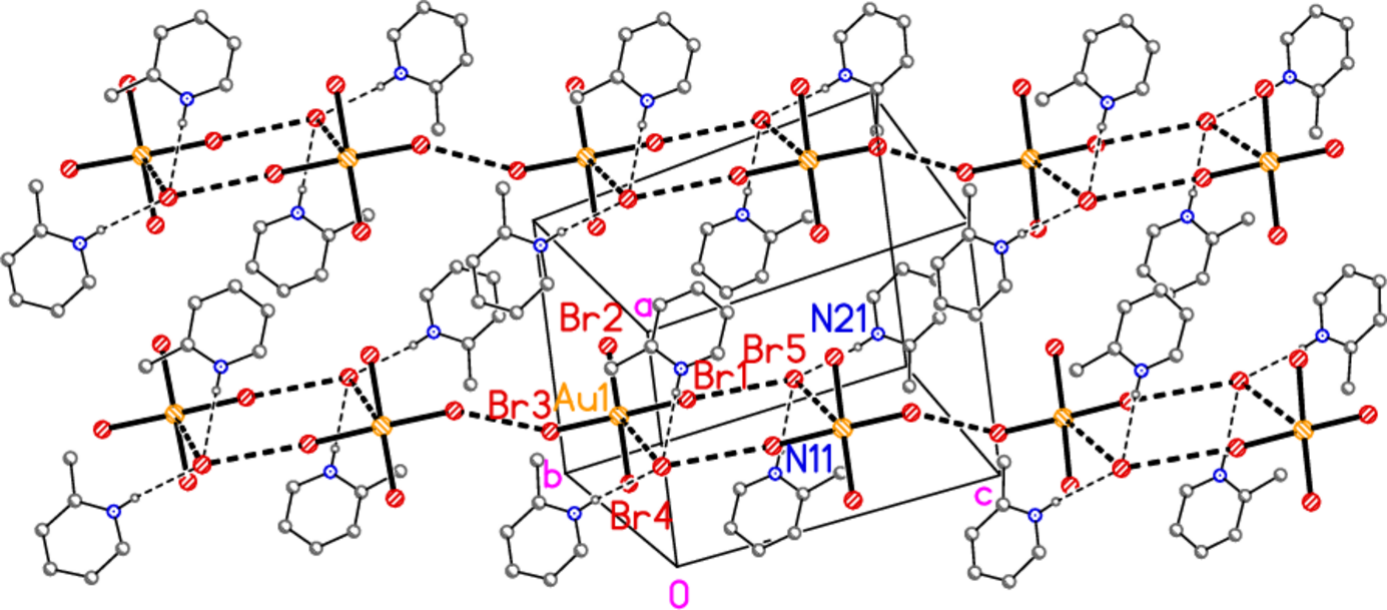
The packing of compound **3**, viewed perpendicular to (011), showing chains of residues parallel to [01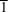]. Dashed lines indicate Au⋯Br and Br⋯Br contacts (thick) or hydrogen bonds (thin).

**Figure 13:**
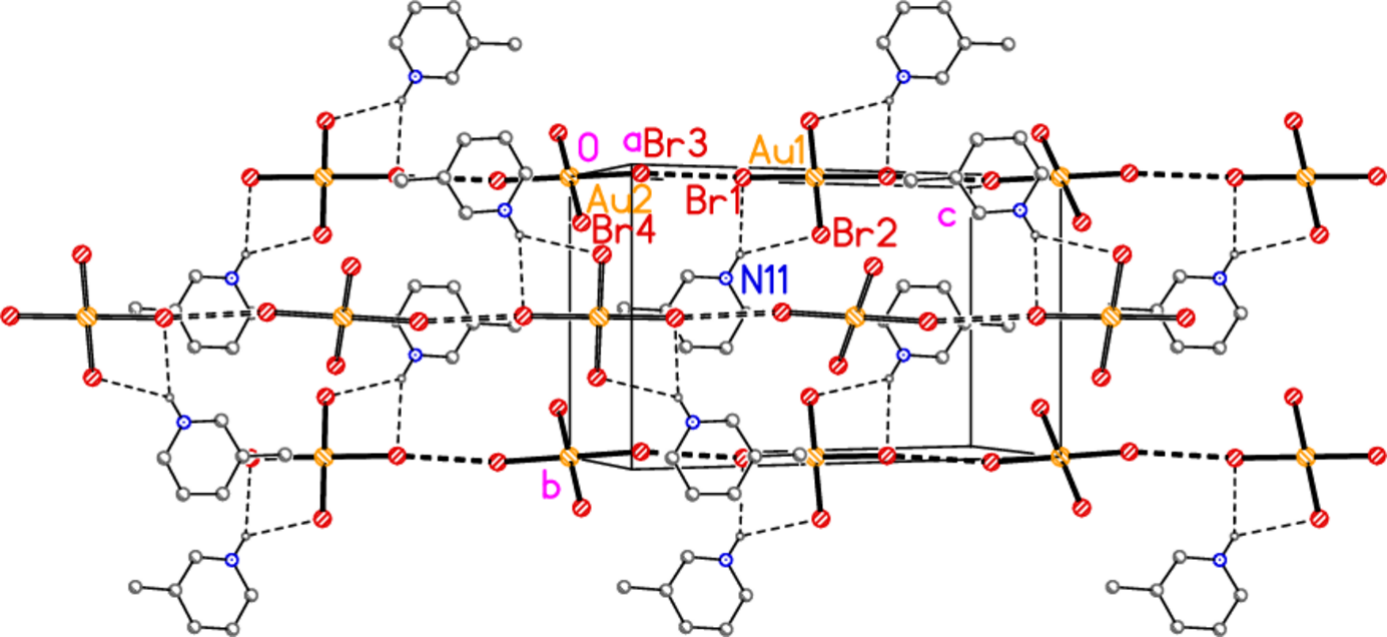
The packing of compound **4**, viewed perpendicular to (10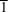), showing chains of residues parallel to [101]. Dashed lines indicate Br⋯Br contacts (thick) or hydrogen bonds (thin). Contacts of the type Br⋯π are not drawn explicitly, because they are almost parallel to the view direction; however, these can be recognized *e.g.* for the ring based on N11, from the ring centre to the bromine atom overlapped with the left-hand edge of this ring. This is a simplified view in which several borderline contacts are not included (see text).

**Figure 14:**
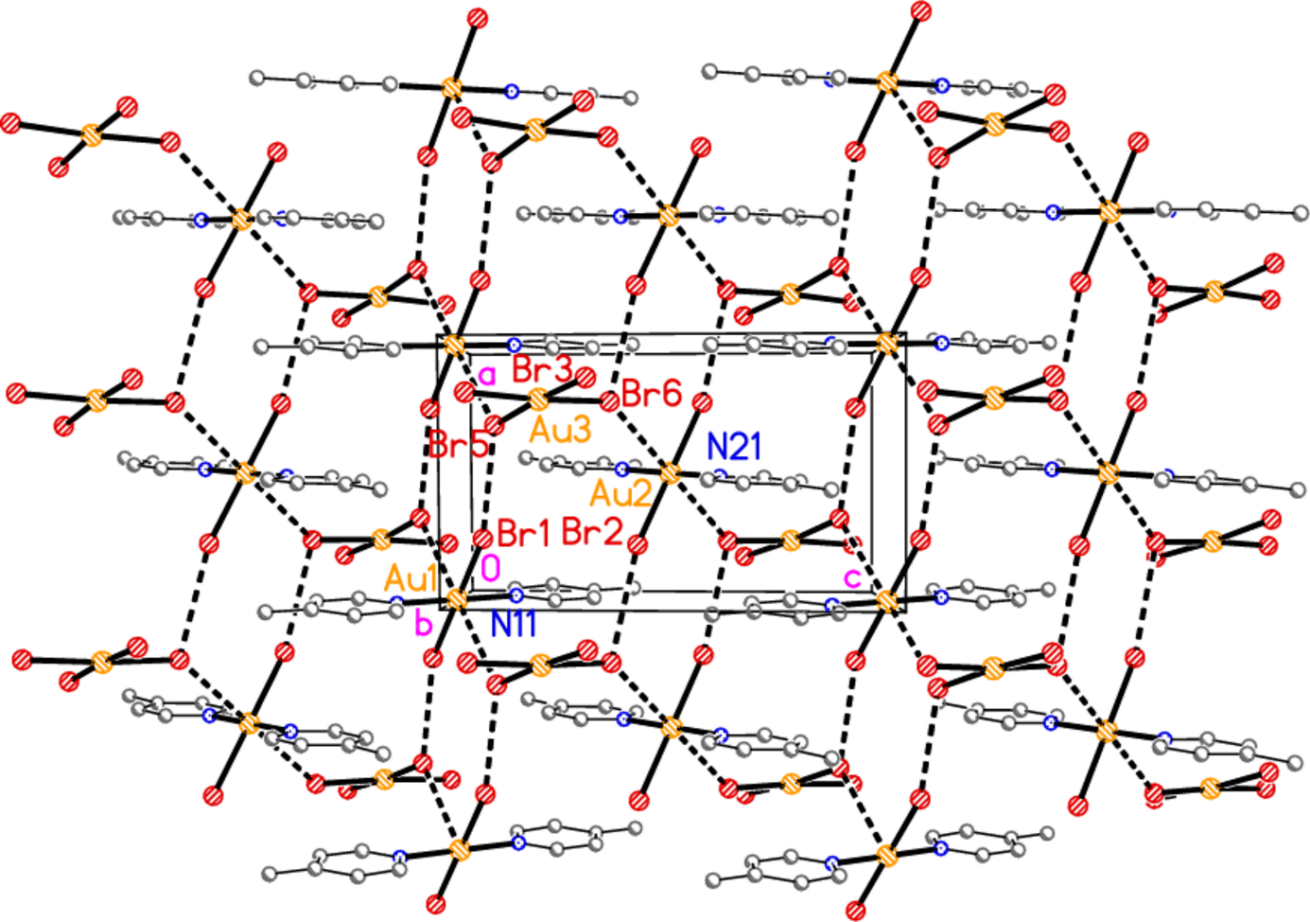
Packing diagram of compound **5** (without solvent), showing the layer structure parallel to the *ac* plane; the view direction is perpendicular to that plane. Thick dashed lines indicate Br⋯Br or Au⋯Br contacts.

**Figure 15:**
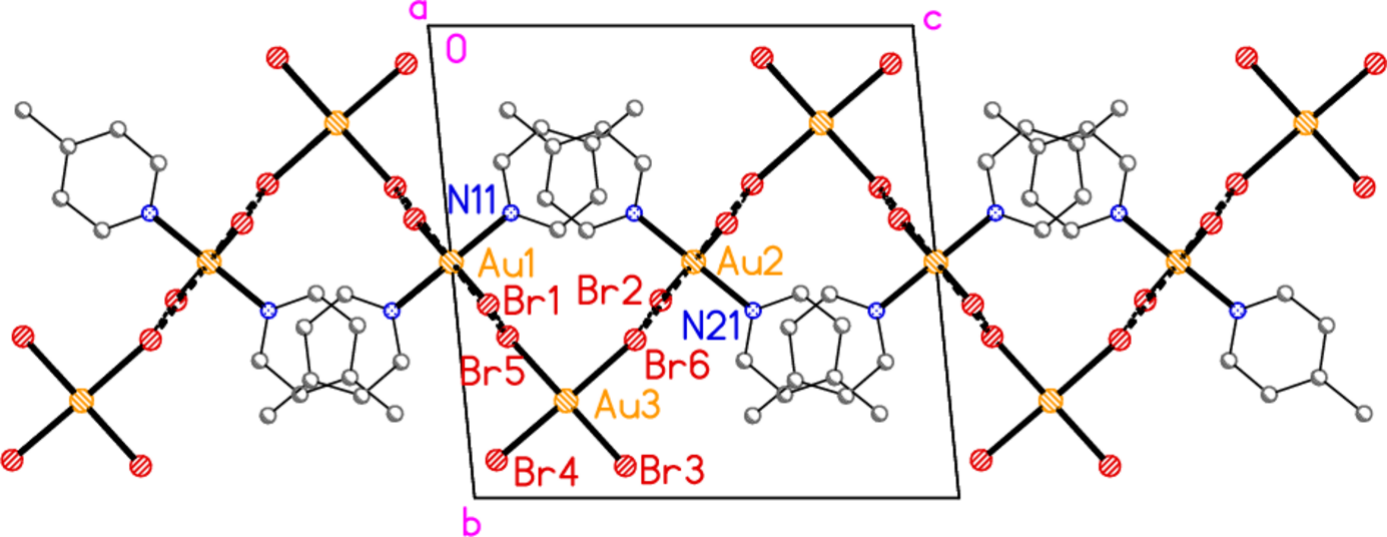
The packing of compound **5** projected parallel to the *a* axis, showing the corrugation of the layer.

**Figure 16:**
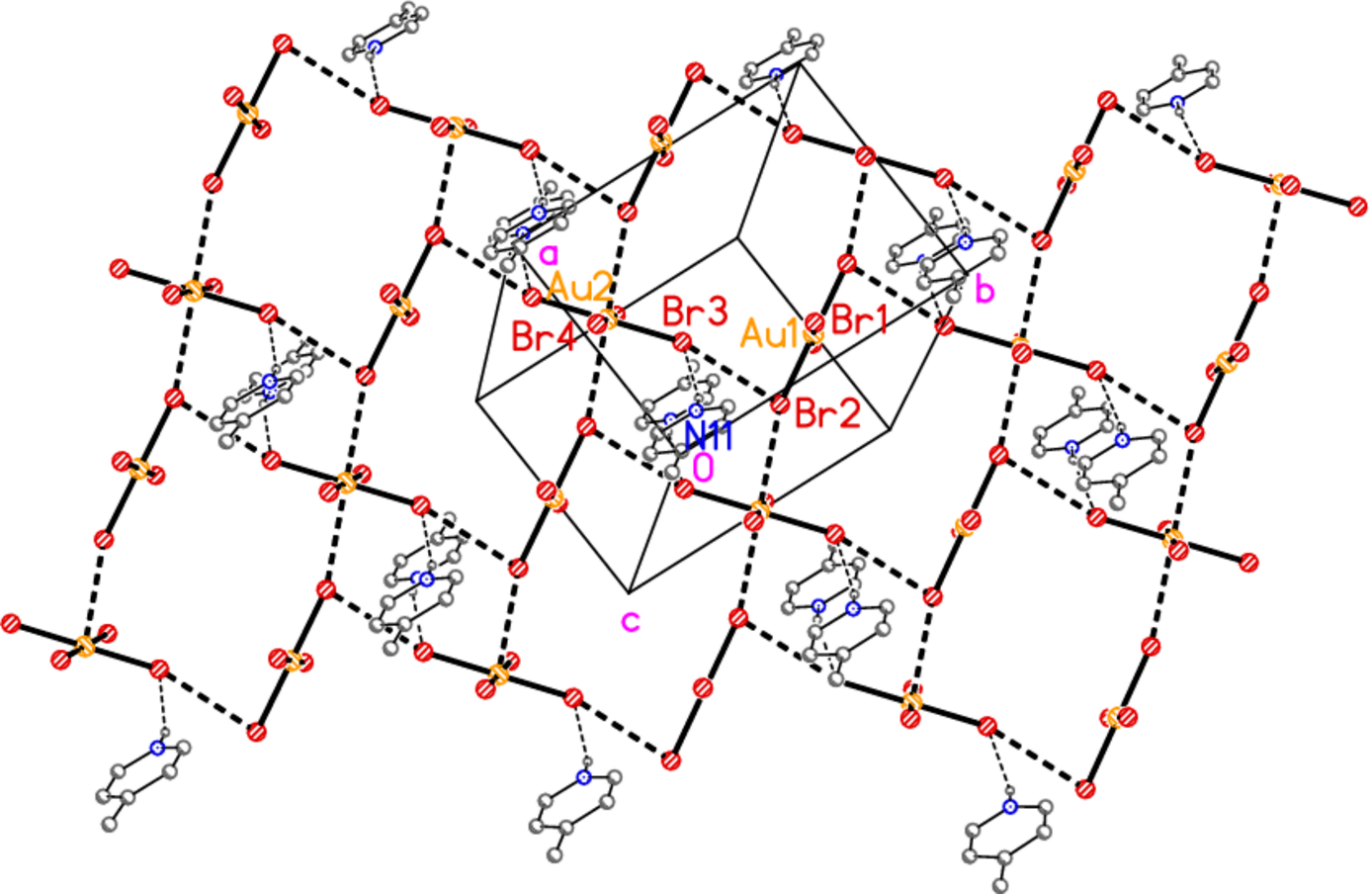
Packing diagram of compound **6**, showing the layer structure parallel to the *ab* plane; the view direction is perpendicular to that plane in the region *z* ≃ 1. Dashed lines indicate Br⋯Br or Au⋯Br contacts (thick) or hydrogen bonds (thin).

**Figure 17:**
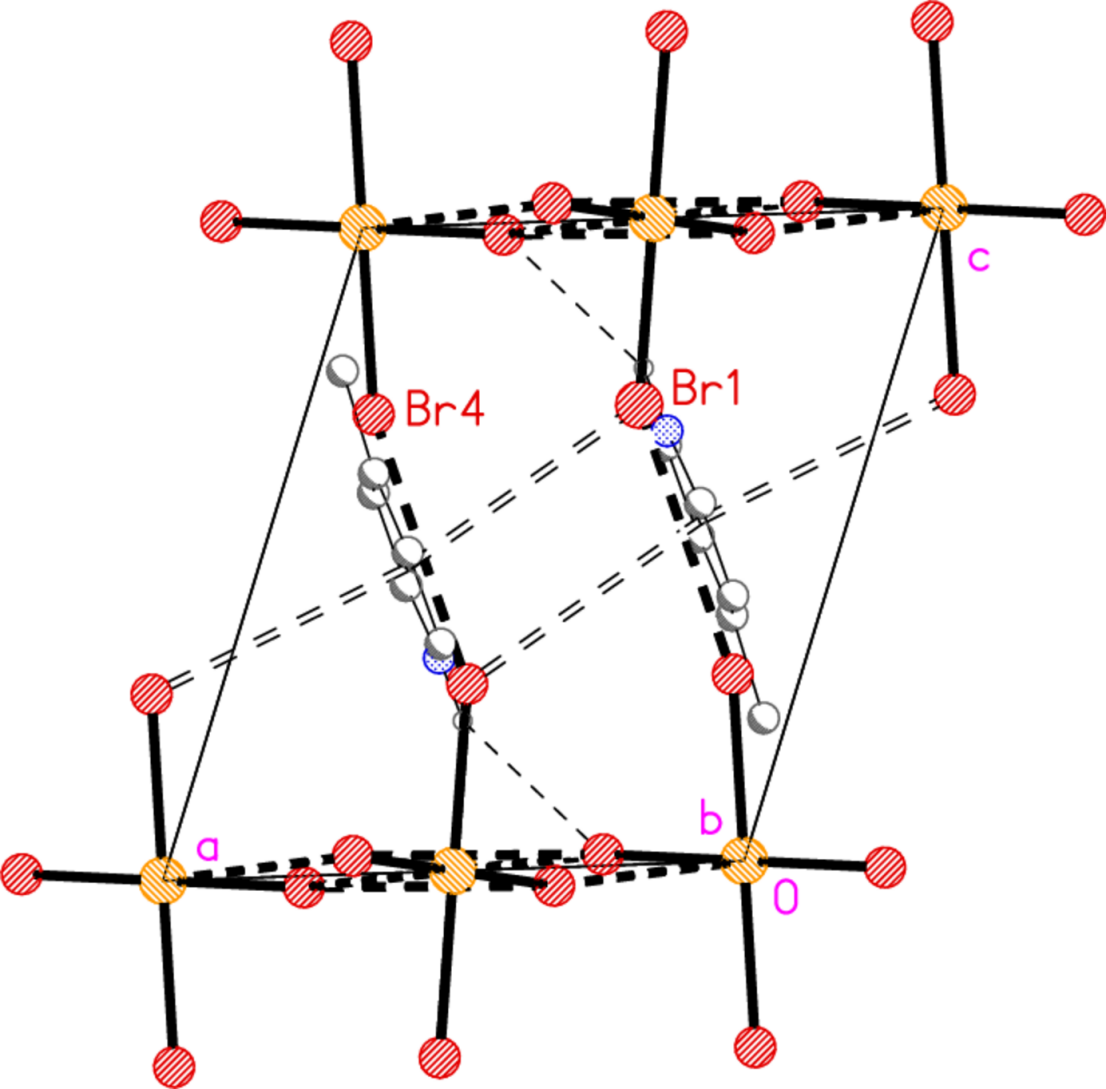
The packing of compound **6** projected parallel to the *b* axis, showing the Br⋯π contacts (open dashed bonds) and the linkage of the layers at *z* ≃ 0 and 1 by the contacts Br1⋯Br4.

**Figure 18:**
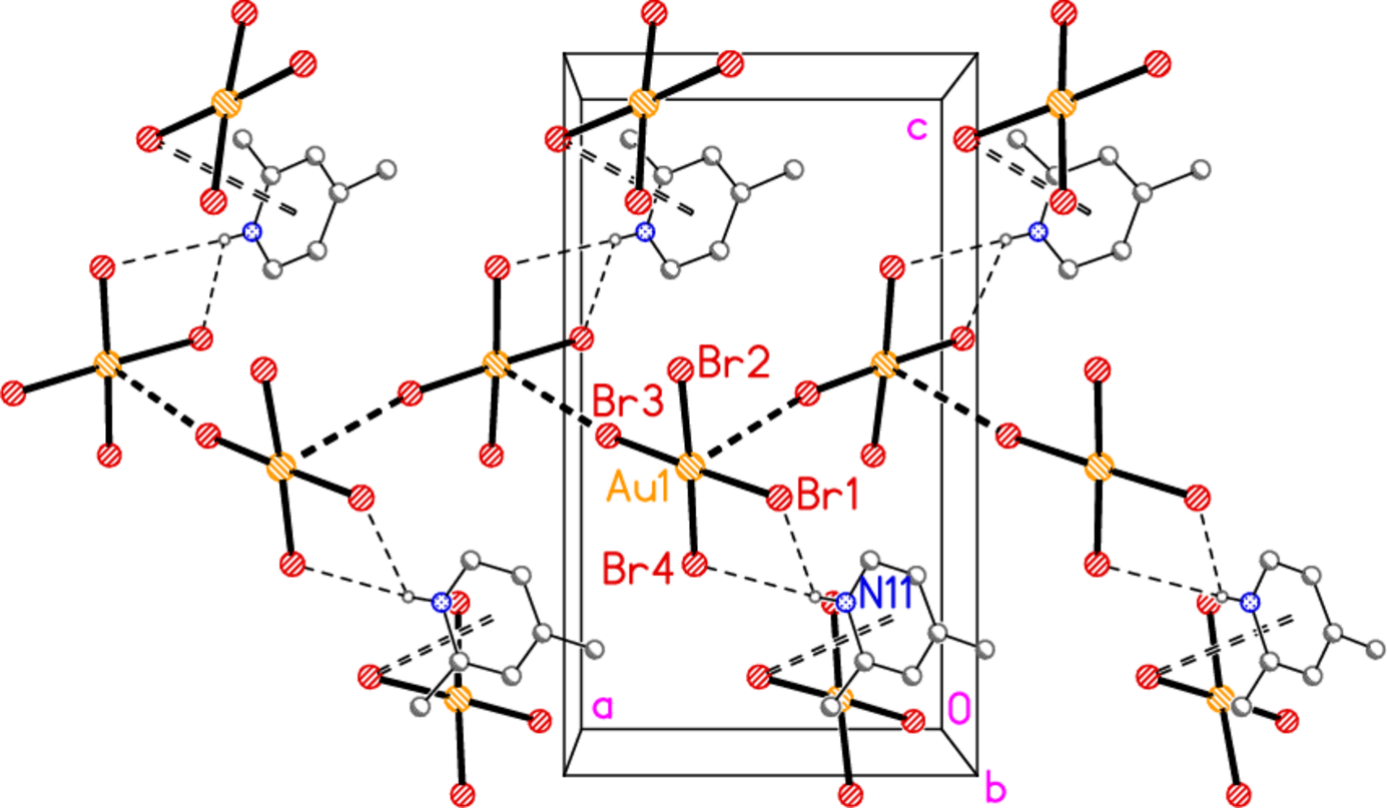
The packing of compound **7** viewed parallel to the *b* axis in the range *y* ≃ 0.75. Dashed lines indicate Au⋯Br contacts (thick) or hydrogen bonds (thin). The main ribbon of residues (see text) runs horizontally in the centre of the diagram; peripheral Br⋯π contacts (two further ribbons) are shown top and bottom as open dashed bonds.

The packing of compound **7** displays fewer striking features than the other structures. For structures in space group *P*2_1_2_1_2_1_, it is often difficult to produce easily inter­pretable packing diagrams, because the combination of mutually perpendicular 2_1_ axes seldom produces motifs that are easily shown in two dimensions. This generalization also holds for **7**. The asymmetric unit (Fig. 7 ▸) shows the three-centre classical hydrogen bond. This combines with the coinage bond Au1⋯Br3 (−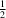 + *x*, 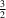 − *y*, 1 − *z*) = 3.6391 (7) Å to produce a ribbon of residues parallel to the *a* axis, seen clearly running horizontally through the centre of Fig. 18 ▸. However, the further, longer, contacts Br1⋯Br4(1 − *x*, 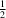 + *y*, 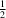 − *z*) = 3.7854 (10), Br2⋯Br4(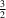 − *x*, 2 − *y*, 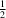 + *z*) = 3.8126 (9) and Br1⋯*Cg*(1 − *x*, 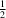 + *y*, 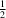 − *z*) = 3.735 (3) Å involve the other two screw axes. Only the peripheral Br⋯π contacts are also shown in Fig. 18 ▸. The Au1—Br1⋯*Cg* angle is extremely narrow at 77.2°, associated with an Au1⋯*Cg* distance of 3.980 (3) Å.

The recent papers in this series have shown some uncommon packing motifs. We reported several examples of linear Au—*X*⋯*X*—Au groupings, where *X* = Cl or Br, in an earlier paper (Döring & Jones, 2016 ▸), and a literature search appeared in part 18 (Döring & Jones, 2025*a* ▸). The first type of inter­action is reminiscent of the classical halogen bond C—*X*⋯*X*—C, for which two types were differentiated by Pedireddi *et al.* (1994 ▸) in terms of the C—*X*⋯*X* angles; type 1 with both angles approximately equal and type 2 with angles of approximately 90 and 180°. The latter were thought to be more significant, and were inter­preted in terms of a σ hole in the extension of one C—*X* bond. We are however not aware of any similar theoretical treatment of Au—*X*⋯*X*—Au contacts. Another motif is the stacking of pyridinium rings and square-planar [Au*X*_4_]^−^ ions (where *X* = Cl or Br), for which a literature search was reported in the previous paper (Döring & Jones, 2025*b* ▸). A third type of motif consists of Au—*X*⋯π contacts (generally with narrow angles at the *X* atom), for which we are also unaware of any theoretical analysis.

## 4. Database survey

The searches employed the routine ConQuest (Bruno *et al.*, 2002 ▸), part of Version 2025.1.1 of the Cambridge Structural Database (Groom *et al.*, 2016 ▸). In the first search, systems involving four-coordinate gold with two coordinated halogen atoms and two coordinated pyridines (including substituted pyridines) were sought. Only four compounds were found, all involving cations with a *trans* configuration at the gold atom.

The oldest such structure is the pyridine derivative *trans*-[Py_2_AuCl_2_]Cl·H_2_O, part of the pioneering work of Strähle in establishing the structures of ‘simple’ gold complexes (refcode BENYEY; Adams & Strähle, 1982 ▸), later redetermined (BENYEY01) by Bowling *et al.* (2023 ▸). The structure [(3-Lut)_2_AuCl_2_]SbF_6_ was included in Part 2 of this series (HILNOF; Jones & Ahrens, 1998 ▸). The ternary Au^III^ derivative [Py_2_AuBr_2_]·2[PyAuBr_3_]·[AuBr_4_] (WOQMEU; Peters *et al.*, 2000 ▸) and its chlorine analogue (KILFIV; Bourosh *et al.*, 2007 ▸) were also found. The two structures appear to be isotypic; curiously, the newer reference does not mention the older one.

**Figure 19:**
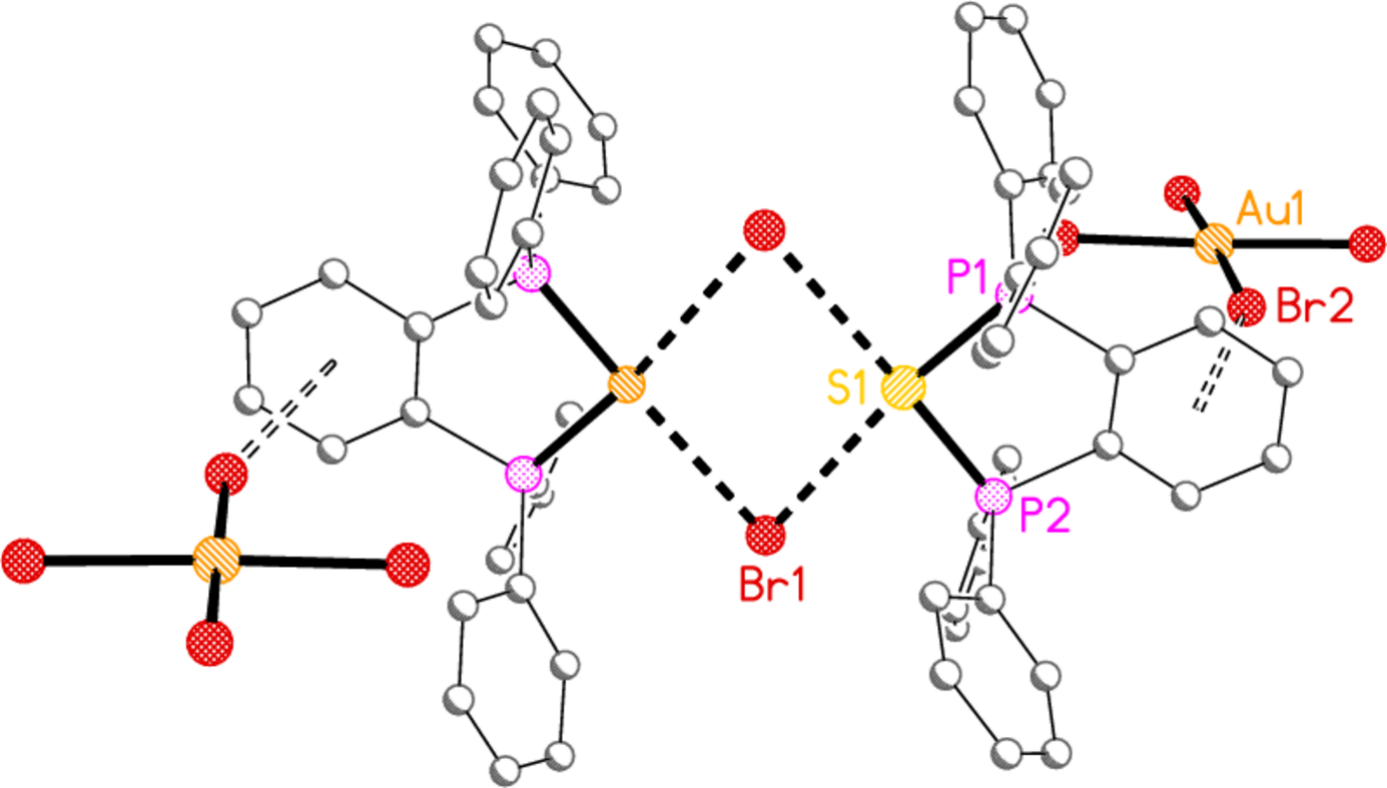
One formula unit of 1,1,3,3-tetra­phenyl-1,3-di­hydro-2,1,3-benzo­thiadi­phosphole-1,3-diium bromide tetra­bromido­aurate(III) di­chloro­methane hemisolvate (Taouss & Jones, 2011 ▸), excluding solvent, showing the short S⋯Br^−^ and Au—Br⋯π contacts (full and open dashed bonds respectively); only the former were discussed at the time. The ensemble displays crystallographic inversion symmetry. The other independent formula unit shows no Br⋯π contacts, but is instead involved in Br⋯Cl contacts to the solvent mol­ecule.

In the second search, the shortest (< 3.5 Å) Br⋯π contacts from [AuBr_4_]^−^ ions to aromatic six-membered rings (containing any combination of C and N atoms) were sought. This gave five hits; the first three involve nitro­gen heterocycles. In bis­(2,2′-bi­pyridine)­dibromido­gold(III) di­bromido­aurate(I) tetra­bromido­aurate(III) (AHOFAG; compound **10** in Chernyshev *et al.*, 2015 ▸), the distance of 3.482 Å may correspond to a stacking inter­action of the anion and cation, with the Au—Br⋯π angle of 88.5° corresponding to an almost parallel orientation of the two moieties. In 2-(quinolin-2-yl)quinolinium tetra­bromido­aurate(III) (AHOGIP; compound **18**, *ibid*.) the distance is 3.489 Å and the angle 98.1°. In di­bromido-(2,2′-bi­pyridine)­gold(III) tetra­bromido­aurate(III) (XEMCEY01; compound **11b** in Hayoun *et al.*, 2006 ▸) the distance is 3.424 Å and the angle rather wider at 120.6°. The final two hits involve phenyl rings; both come from our own work, but we did not report the Br⋯π contacts at the time. In 5-(diphen­yl(bromo)­phospho­nio)[2.2]para­cyclo­phane tetra­bromido­aurate(III) (BOKNOH; compound **5** in Upmann *et al.*, 2019 ▸), the distance is 3.492 Å and the angle 164.6°, whereas in 1,1,3,3-tetra­phenyl-1,3-di­hydro-2,1,3-benzo­thiadi­phosphole-1,3-diium bromide tetra­bromido­aurate(III) di­chloro­methane hemisolvate (ODAWOH; compound **3** in Taouss & Jones, 2011 ▸; Fig. 19 ▸), the distance is 3.447 Å and the angle 156.9°. We note that the Au—Br⋯π angles differ greatly between systems involving heterocyclic or phenyl rings.

## 5. Synthesis and crystallization

Compound **1**: In an attempt to obtain single crystals of tri­chlorido­(2-picoline)gold(III), a sample was dissolved in di­chloro­methane and the solution was overlayered with diisopropyl ether. Yellow irregular blocks of **1** were obtained. Analysis: calculated C 16.65, H 1.86, N 3.24; found C 16.88, H 1.77, N 3.20%.

Compound **2**: In an attempt to obtain single crystals of tri­bromido­(2-picoline)gold(III), 90 mg of bromido­(tetra­hydro­thio­phene)­gold(I) were added to 2 mL of 2-picoline and suspended overnight using an ultrasonic bath. The white solid product, assumed to be bis­(2-picoline)gold(I) di­bromido­aurate(I), was suspended in 2 mL of di­chloro­methane, and two drops of elemental bromine were added. The solution was distributed over five ignition tubes and overlayered with various precipitants. In the tube using diisopropyl ether, crystals of **2** in the form of red hexa­gonal plates were obtained. Analysis: calculated C 11.80, H 1.32, N 2.29; found C 12.00, H 1.32, N 2.44%.

Compound **3**: A further attempt to obtain single crystals of tri­bromido­(2-picoline)gold(III), using slightly varied amounts, led to red plates of **3** when diisopropyl ether was used as precipitant.

Compound **4**: Crystallization attempts analogous to those producing **2**, but using 3-picoline, led to red blocks of **4**. Analysis: calculated C 11.80, H 1.32, N 2.29; found C 12.11, H 1.40, N 2.41%.

Compound **5**: In an attempt to obtain single crystals of tri­bromido­(4-picoline)gold(III), 40 mg of bis­(4-picoline)gold(I) di­bromido­aurate(I) were dissolved in 2.5 mL of nitro­methane, and 3 drops of elemental bromine were added. Crystallization attempts as above led to red plates of **5** when diethyl ether was used as precipitant.

Compound **6**: A further attempt to obtain single crystals of tri­bromido­(4-picoline)gold(III), using di­chloro­methane as solvent (as above for **2**), led to red plates of **6** when diethyl ether was used as precipitant.

More details are given in the PhD thesis of CD (Döring, 2016 ▸). However, details of the crystallization of **7** have unfortunately been lost.

## 6. Refinement

Details of the measurements and refinements are given in Table 15 ▸. Structures were refined anisotropically on *F*^2^. Hydrogen atoms of the rings were included at calculated positions and refined using a riding model with C—H = 0.95 Å. Methyl groups were included as idealized rigid groups with C—H = 0.98 Å and H—C—H = 109.5°, and were allowed to rotate but not tip (command ‘AFIX 137’), but the methyl hydrogen-atom positions thus determined should be inter­preted with caution in the presence of heavy atoms. *U* values of the hydrogen atoms were fixed at 1.5 × *U*_eq_ of the parent carbon atoms for methyl groups and 1.2 × *U*_eq_ of the parent carbon atoms for other hydrogen atoms.

*Exceptions and special features*

Compound **1**: The crystal was a non-merohedral twin (by 180° rotation about the *b* axis). The structure was refined using the ‘HKLF 5’ method. The scale factor (relative volume of the second twinning component) refined to 0.1823 (7). The twin data reduction merges equivalent reflections, so that *R*_int_ is meaningless. The intensity dataset comprised all non-overlapped reflections from the major component and all overlapped reflections, so that the number of reflections should be inter­preted with caution. The NH hydrogen atoms were refined freely but with N—H distances restrained to be approximately equal (‘SADI’).

Compound **2**: The structure was a non-merohedral twin (by 180° rotation about the vector b* + c*). The structure was refined using the ‘HKLF 5’ method. Although the relative volume of the smaller component was only 0.0400 (5), the results were significantly improved compared to a non-twin refinement. The twin data reduction merges equivalent reflections, so that *R*_int_ is meaningless. The intensity dataset comprised all non-overlapped reflections from the major component and all overlapped reflections, so that the number of reflections should be inter­preted with caution. The atoms of the second picolinium cation were disordered, and the two positions were refined using the restraint ‘SAME’. The atoms of the minor disorder component were refined isotropically. Appropriate constraints and restraints (‘RIGU’ for the major component, ‘SIMU’ and idealized ‘phen­yl’ ring geometry for the minor component, which had an occupation factor of only 0.184 (11)) were employed to improve refinement stability, but the dimensions of disordered groups should always be inter­preted with caution. In the discussion, only the major disorder position is presented. The low goodness-of-fit is probably attributable to the weak data. The low completeness (96%) is probably caused by the ‘remove outliers’ option employed during the data reduction. The NH hydrogen atoms were refined using a riding model with N—H 0.88 Å and *U*(H) fixed at 1.2 × *U*_eq_ of the parent nitro­gen atoms.

Compound **3**: The NH hydrogen atoms were refined freely but with N—H distances restrained to be approximately equal (‘SADI’).

Compounds **4** and **6**: The NH hydrogen atoms were refined freely.

Compound **5**: The crystal was a non-merohedral twin by 180° rotation about the *b* axis. The structure was refined using the ‘HKLF 5’ method. The scale factor (relative volume of the second twinning component) refined to 0.4557 (6). The detwinning routines merge equivalent reflections, so that *R*_int_ is meaningless. The intensity dataset comprised all non-overlapped reflections from both components and all overlapped reflections, so that the number of reflections should be inter­preted with caution. For some unexplained reason, the *U* values of the anion and cation are unusually low, which led to problems in refining the light atoms anisotropically; *U* values of the cation C and N atoms were restrained to be approximately isotropic (thus avoiding NPD atoms) using the command ‘ISOR’. The solvent mol­ecule is badly resolved and has high *U* values, but no disorder model could be developed (and the occupation factor, when freely refined, had a value close to 1). It was refined isotropically. A referee has correctly commented that the ‘ISOR’ restraint is quite harsh, so that an isotropic refinement of the light atoms might be better. This is a moot point; our final decisions to refine the solvent isotropically and the cation C and N atoms anisotropically with restraints are clearly to some extent subjective.

Compound **7**: The NH hydrogen atom was refined freely. Slow convergence of the methyl hydrogen atoms at C18 may indicate some rotational disorder of this group. The compound is achiral and crystallizes only by chance in a Sohncke space group. An extinction correction was applied, whereby the extinction coefficient, as implemented in *SHELXL2019* (Sheldrick, 2015 ▸), refined to 0.00101 (7).

## Supplementary Material

Crystal structure: contains datablock(s) 1, 2, 3, 4, 5, 6, 7, global. DOI: 10.1107/S2056989025008801/yz2072sup1.cif

Structure factors: contains datablock(s) 1. DOI: 10.1107/S2056989025008801/yz20721sup2.hkl

Structure factors: contains datablock(s) 2. DOI: 10.1107/S2056989025008801/yz20722sup3.hkl

Structure factors: contains datablock(s) 3. DOI: 10.1107/S2056989025008801/yz20723sup4.hkl

Structure factors: contains datablock(s) 4. DOI: 10.1107/S2056989025008801/yz20724sup5.hkl

Structure factors: contains datablock(s) 5. DOI: 10.1107/S2056989025008801/yz20725sup6.hkl

Structure factors: contains datablock(s) 6. DOI: 10.1107/S2056989025008801/yz20726sup7.hkl

Structure factors: contains datablock(s) 7. DOI: 10.1107/S2056989025008801/yz20727sup8.hkl

CCDC references: 2145217, 2145214, 2145215, 2145213, 2145230, 2145210, 2145209

Additional supporting information:  crystallographic information; 3D view; checkCIF report

## Figures and Tables

**Figure 6:**
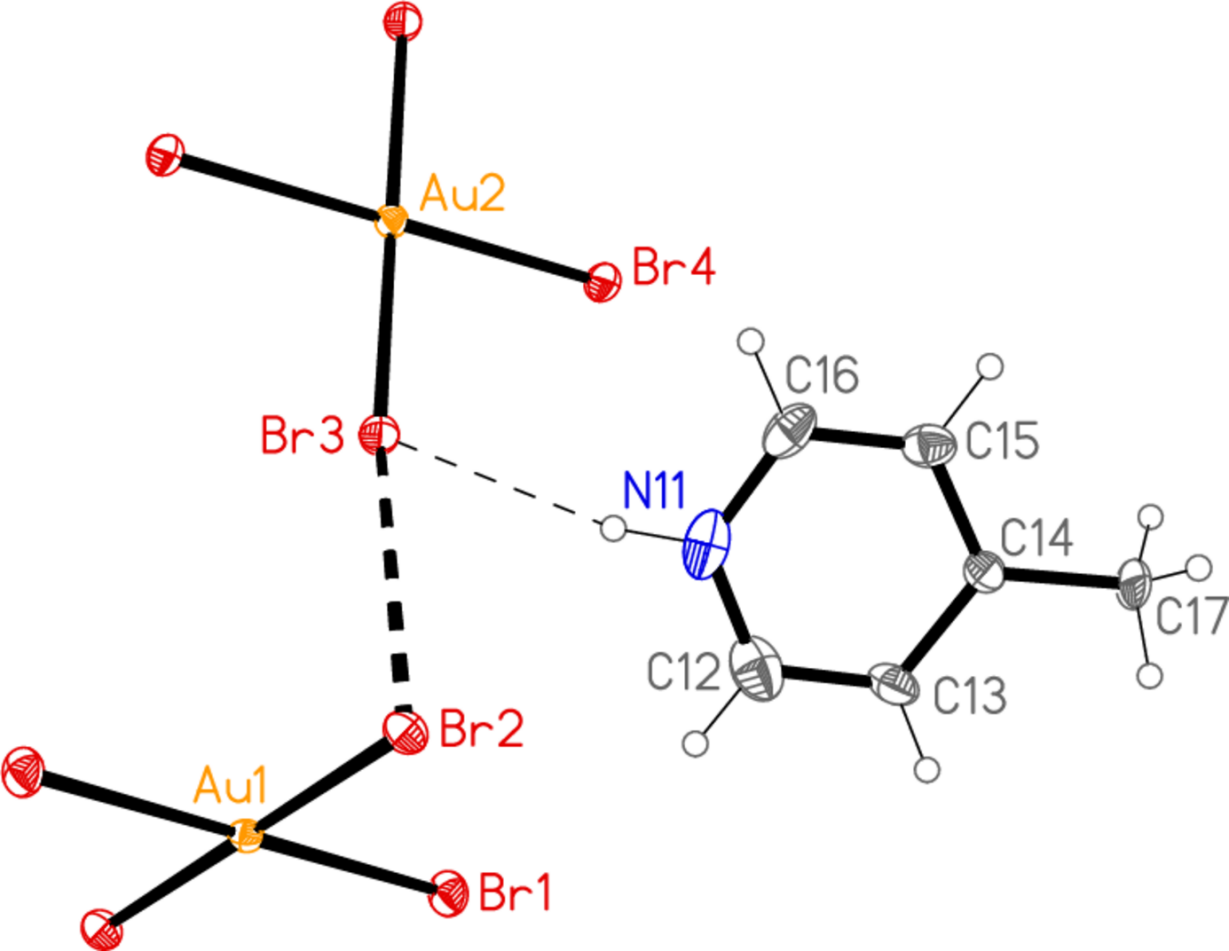
The asymmetric unit of compound **6** in the crystal, extended by symmetry to form complete anions. Dashed lines indicate a hydrogen bond (thin) or a Br⋯Br contact (thick).

**Table 1:** Selected geometric parameters (Å, °) for **1**[Chem scheme1]

Au1—Cl2	2.2752 (10)	Au2—Cl7	2.2737 (10)
Au1—Cl3	2.2814 (10)	Au2—Cl5	2.2832 (11)
Au1—Cl4	2.2827 (10)	Au2—Cl6	2.2851 (10)
Au1—Cl1	2.2837 (10)	Au2—Cl8	2.2852 (10)
			
Cl2—Au1—Cl3	89.73 (4)	Cl7—Au2—Cl6	89.60 (4)
Cl2—Au1—Cl4	179.35 (4)	Cl5—Au2—Cl6	90.19 (4)
Cl3—Au1—Cl4	90.13 (4)	Cl7—Au2—Cl8	90.69 (4)
Cl2—Au1—Cl1	90.81 (4)	Cl5—Au2—Cl8	89.52 (4)
Cl3—Au1—Cl1	177.95 (4)	Cl6—Au2—Cl8	179.31 (4)
Cl4—Au1—Cl1	89.35 (4)	C16—N11—C12	123.5 (4)
Cl7—Au2—Cl5	179.62 (4)	C26—N21—C22	123.8 (4)

**Table 2:** Selected geometric parameters (Å, °) for **2**[Chem scheme1]

Au1—Br2	2.4157 (12)	Au2—Br7	2.4135 (13)
Au1—Br3	2.4235 (11)	Au2—Br6	2.4206 (13)
Au1—Br1	2.4283 (11)	Au2—Br8	2.4215 (12)
Au1—Br4	2.4290 (12)	Au2—Br5	2.4253 (12)
			
Br2—Au1—Br3	90.20 (4)	Br7—Au2—Br8	90.20 (5)
Br2—Au1—Br1	90.21 (4)	Br6—Au2—Br8	177.40 (5)
Br3—Au1—Br1	178.16 (5)	Br7—Au2—Br5	177.75 (5)
Br2—Au1—Br4	178.64 (5)	Br6—Au2—Br5	89.33 (4)
Br3—Au1—Br4	89.88 (4)	Br8—Au2—Br5	89.98 (4)
Br1—Au1—Br4	89.75 (4)	C12—N11—C16	124.1 (10)
Br7—Au2—Br6	90.58 (5)	C22—N21—C26	124.9 (11)

**Table 3:** Selected geometric parameters (Å, °) for **3**[Chem scheme1]

Au1—Br1	2.4206 (4)	Au1—Br3	2.4243 (4)
Au1—Br4	2.4232 (4)	Au1—Br2	2.4314 (4)
			
Br1—Au1—Br4	90.687 (14)	Br4—Au1—Br2	175.465 (16)
Br1—Au1—Br3	177.503 (16)	Br3—Au1—Br2	89.255 (15)
Br4—Au1—Br3	91.130 (15)	C16—N11—C12	124.4 (4)
Br1—Au1—Br2	89.069 (14)	C22—N21—C26	124.5 (4)

**Table 4:** Selected geometric parameters (Å, °) for **4**[Chem scheme1]

Au1—Br1	2.4241 (6)	Au2—Br3	2.4207 (6)
Au1—Br2	2.4284 (6)	Au2—Br4	2.4251 (6)
			
Br1^i^—Au1—Br1	180.0	Br3—Au2—Br4	90.11 (2)
Br1—Au1—Br2	89.68 (2)	Br3—Au2—Br4^ii^	89.89 (2)
Br1—Au1—Br2^i^	90.33 (2)	Br4—Au2—Br4^ii^	180.0
Br2—Au1—Br2^i^	180.0	C12—N11—C16	123.5 (6)
Br3^ii^—Au2—Br3	180.0		

Symmetry codes: (i) 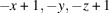; (ii) 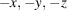.

**Table 5:** Selected geometric parameters (Å, °) for **5**[Chem scheme1]

Au1—N11	2.032 (8)	Au3—Br3	2.4130 (12)
Au1—Br1	2.4220 (10)	Au3—Br4	2.4201 (12)
Au2—N21	2.019 (8)	Au3—Br6	2.4257 (12)
Au2—Br2	2.4214 (11)	Au3—Br5	2.4258 (12)
			
N11^i^—Au1—N11	180.0	Br4—Au3—Br6	176.54 (5)
N11—Au1—Br1	89.7 (2)	Br3—Au3—Br5	176.38 (4)
N11—Au1—Br1^i^	90.3 (2)	Br4—Au3—Br5	90.39 (4)
Br1—Au1—Br1^i^	180.0	Br6—Au3—Br5	89.81 (4)
N21^ii^—Au2—N21	180.0 (4)	C16—N11—C12	120.3 (9)
N21—Au2—Br2	90.3 (2)	C16—N11—Au1	120.8 (7)
N21—Au2—Br2^ii^	89.7 (2)	C12—N11—Au1	118.9 (6)
Br2—Au2—Br2^ii^	180.0	C22—N21—C26	119.5 (9)
Br3—Au3—Br4	89.83 (4)	C22—N21—Au2	120.9 (7)
Br3—Au3—Br6	90.19 (4)	C26—N21—Au2	119.5 (7)

Symmetry codes: (i) 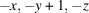; (ii) 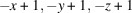.

**Table 6:** Selected geometric parameters (Å, °) for **6**[Chem scheme1]

Au1—Br1	2.4240 (6)	Au2—Br3	2.4282 (6)
Au1—Br2	2.4282 (6)	Au2—Br4	2.4340 (6)
			
Br1^i^—Au1—Br1	180.0	Br3—Au2—Br4	90.21 (2)
Br1—Au1—Br2	89.23 (2)	Br3—Au2—Br4^ii^	89.79 (2)
Br1—Au1—Br2^i^	90.77 (2)	Br4—Au2—Br4^ii^	180.0
Br2—Au1—Br2^i^	180.0	C12—N11—C16	122.9 (7)
Br3^ii^—Au2—Br3	180.0 (3)		

Symmetry codes: (i) 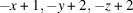; (ii) 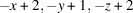.

**Table 7:** Selected geometric parameters (Å, °) for **7**[Chem scheme1]

Au1—Br2	2.4151 (7)	Au1—Br3	2.4247 (7)
Au1—Br4	2.4217 (7)	Au1—Br1	2.4310 (7)
			
Br2—Au1—Br4	175.99 (3)	Br4—Au1—Br1	89.69 (2)
Br2—Au1—Br3	89.73 (2)	Br3—Au1—Br1	177.57 (3)
Br4—Au1—Br3	90.40 (3)	C16—N11—C12	124.0 (7)
Br2—Au1—Br1	90.35 (2)		

**Table 8:** Hydrogen-bond geometry (Å, °) for **1**[Chem scheme1]

*D*—H⋯*A*	*D*—H	H⋯*A*	*D*⋯*A*	*D*—H⋯*A*
N11—H01⋯Cl1	0.88 (3)	2.66 (3)	3.510 (4)	163 (4)
N11—H01⋯Cl4	0.88 (3)	2.96 (4)	3.562 (4)	127 (3)
N21—H02⋯Cl5	0.87 (3)	2.77 (3)	3.421 (3)	132 (3)
N21—H02⋯Cl8	0.87 (3)	2.93 (3)	3.756 (4)	159 (3)
C13—H13⋯Cl4^i^	0.95	2.88	3.756 (4)	154
C13—H13⋯Cl7^i^	0.95	2.96	3.639 (4)	129
C16—H16⋯Cl4	0.95	2.83	3.505 (5)	129
C16—H16⋯Cl7	0.95	2.74	3.621 (4)	154
C17—H17*C*⋯Cl7^ii^	0.98	2.86	3.691 (4)	144
C23—H23⋯Cl5^iii^	0.95	2.88	3.803 (4)	164
C25—H25⋯Cl3^iv^	0.95	2.93	3.811 (5)	156
C26—H26⋯Cl2^iv^	0.95	2.72	3.635 (4)	161
C26—H26⋯Cl5	0.95	2.88	3.484 (5)	123
C27—H27*B*⋯Cl2^v^	0.98	2.87	3.841 (4)	170

Symmetry codes: (i) 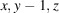; (ii) 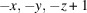; (iii) 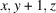; (iv) 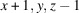; (v) 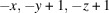.

**Table 9:** Hydrogen-bond geometry (Å, °) for **2**[Chem scheme1]

*D*—H⋯*A*	*D*—H	H⋯*A*	*D*⋯*A*	*D*—H⋯*A*
N11—H01⋯Br4	0.88	2.62	3.422 (9)	153
N21—H02⋯Br5	0.88	2.60	3.369 (10)	147
C13—H13⋯Br2^i^	0.95	3.10	3.783 (11)	131
C16—H16⋯Br4^ii^	0.95	2.96	3.870 (11)	162
C17—H17*A*⋯Br1	0.98	3.04	3.800 (11)	136
C17—H17*A*⋯Br6^iii^	0.98	3.11	3.796 (10)	128
C17—H17*C*⋯Br1^iii^	0.98	3.03	3.706 (11)	127
C23—H23⋯Br7^i^	0.95	3.11	3.765 (11)	128
C25—H25⋯Br3^i^	0.95	2.95	3.894 (13)	171
C26—H26⋯Br5^iv^	0.95	2.86	3.811 (13)	177
C27—H27*A*⋯Br1^iii^	0.98	3.08	3.940 (13)	147
C27—H27*B*⋯Br4^v^	0.98	3.03	3.994 (13)	169
C27—H27*C*⋯Br8^vi^	0.98	3.05	3.814 (13)	136

Symmetry codes: (i) 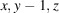; (ii) 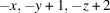; (iii) 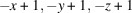; (iv) 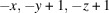; (v) 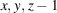; (vi) 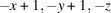.

**Table 10:** Hydrogen-bond geometry (Å, °) for **3**[Chem scheme1]

*D*—H⋯*A*	*D*—H	H⋯*A*	*D*⋯*A*	*D*—H⋯*A*
N11—H01⋯Br5	0.80 (4)	2.43 (4)	3.225 (3)	172 (6)
N21—H02⋯Br5	0.80 (4)	2.39 (4)	3.191 (4)	173 (5)
C13—H13⋯Br1^i^	0.95	3.13	3.961 (4)	146
C15—H15⋯Br2^ii^	0.95	3.07	3.941 (4)	152
C16—H16⋯Br1	0.95	3.08	3.614 (4)	117
C16—H16⋯Br5^iii^	0.95	2.91	3.751 (4)	148
C17—H17*B*⋯Br1^i^	0.98	2.99	3.932 (4)	162
C17—H17*C*⋯Br2^iii^	0.98	3.02	3.758 (4)	133
C26—H26⋯Br2^iv^	0.95	2.81	3.602 (4)	142
C27—H27*A*⋯Br3^iii^	0.98	3.00	3.800 (5)	140

Symmetry codes: (i) 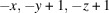; (ii) 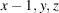; (iii) 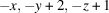; (iv) 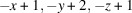.

**Table 11:** Hydrogen-bond geometry (Å, °) for **4**[Chem scheme1]

*D*—H⋯*A*	*D*—H	H⋯*A*	*D*⋯*A*	*D*—H⋯*A*
N11—H01⋯Br1	0.93 (6)	2.61 (6)	3.432 (5)	149 (5)
N11—H01⋯Br2	0.93 (6)	2.84 (6)	3.539 (6)	134 (5)
C12—H12⋯Br3	0.95	2.93	3.874 (7)	175
C15—H15⋯Br1^iii^	0.95	2.87	3.724 (7)	151
C16—H16⋯Br2	0.95	3.05	3.637 (7)	122
C16—H16⋯Br4^iv^	0.95	2.93	3.726 (7)	143

Symmetry codes: (iii) 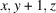; (iv) 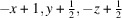.

**Table 12:** Hydrogen-bond geometry (Å, °) for **5**[Chem scheme1]

*D*—H⋯*A*	*D*—H	H⋯*A*	*D*⋯*A*	*D*—H⋯*A*
C12—H12⋯Br4^iii^	0.95	2.99	3.771 (11)	141
C12—H12⋯Br5^iii^	0.95	3.06	3.629 (10)	120
C13—H13⋯O2^iv^	0.95	2.54	3.240 (15)	131
C15—H15⋯Br2^v^	0.95	2.96	3.802 (10)	149
C16—H16⋯Br6^vi^	0.95	3.05	3.826 (10)	140
C22—H22⋯Br5^ii^	0.95	3.04	3.766 (11)	135
C23—H23⋯Br1^vii^	0.95	3.06	3.883 (9)	146
C26—H26⋯Br6	0.95	3.07	3.665 (10)	122
C26—H26⋯O1	0.95	2.39	3.235 (16)	147
C1—H1*B*⋯Br3^vi^	0.98	3.03	3.737 (17)	130

Symmetry codes: (ii) 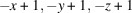; (iii) 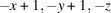; (iv) 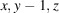; (v) 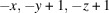; (vi) 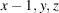; (vii) 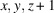.

**Table 13:** Hydrogen-bond geometry (Å, °) for **6**[Chem scheme1]

*D*—H⋯*A*	*D*—H	H⋯*A*	*D*⋯*A*	*D*—H⋯*A*
N11—H01⋯Br2	0.91 (11)	3.04 (10)	3.628 (7)	124 (8)
N11—H01⋯Br3	0.91 (11)	2.56 (11)	3.395 (7)	154 (9)
C12—H12⋯Br1	0.95	2.96	3.872 (8)	160
C15—H15⋯Br1^iii^	0.95	3.07	3.764 (7)	132
C16—H16⋯Br2^iv^	0.95	2.96	3.890 (7)	166
C13—H13⋯Br4^v^	0.95	3.04	3.750 (6)	133

Symmetry codes: (iii) 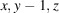; (iv) 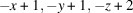; (v) 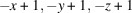.

**Table 14:** Hydrogen-bond geometry (Å, °) for **7**[Chem scheme1]

*D*—H⋯*A*	*D*—H	H⋯*A*	*D*⋯*A*	*D*—H⋯*A*
N11—H01⋯Br4	0.82 (8)	2.82 (7)	3.435 (7)	134 (7)
N11—H01⋯Br1	0.82 (8)	2.92 (8)	3.527 (6)	133 (7)
C15—H15⋯Br2^i^	0.95	2.96	3.622 (7)	128
C18—H18*B*⋯Br2^ii^	0.98	2.94	3.780 (8)	145
C16—H16⋯Br3^i^	0.95	3.03	3.981 (7)	177
C18—H18*A*⋯Br3^iii^	0.98	2.93	3.903 (7)	174
C17—H17*A*⋯Br4^iv^	0.98	3.01	3.862 (8)	146

Symmetry codes: (i) 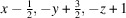; (ii) 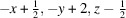; (iii) 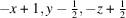; (iv) 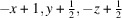.

**Table:** 

	**1**	**2**	**3**	**4**
Crystal data				
Chemical formula	(C_6_H_8_N)[AuCl_4_]	(C_6_H_8_N)[AuBr_4_]	(C_6_H_8_N)_2_[AuBr_4_]Br	(C_6_H_8_N)[AuBr_4_]
*M*_r_	432.90	610.74	784.78	610.74
Crystal system, space group	Triclinic, *P* 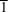	Triclinic, *P* 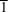	Triclinic, *P* 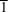	Monoclinic, *P*2_1_/*c*
Temperature (K)	100	100	100	100
*a*, *b*, *c* (Å)	8.0764 (3), 9.0839 (3), 15.3667 (6)	9.9208 (5), 11.9072 (6), 12.2765 (7)	8.7050 (2), 9.1257 (4), 13.8767 (6)	8.1918 (3), 9.3458 (3), 16.1449 (6)
α, β, γ (°)	87.792 (3), 76.132 (3), 85.391 (3)	65.423 (5), 70.506 (5), 68.459 (5)	77.246 (4), 80.023 (3), 61.718 (4)	90, 102.949 (4), 90
*V* (Å^3^)	1090.77 (7)	1198.31 (13)	943.79 (7)	1204.59 (8)
*Z*	4	4	2	4
Radiation type	Mo *K*α	Mo *K*α	Mo *K*α	Mo *K*α
μ (mm^−1^)	14.41	25.57	18.37	25.43
Crystal size (mm)	0.10 × 0.08 × 0.03	0.12 × 0.08 × 0.01	0.20 × 0.12 × 0.06	0.08 × 0.03 × 0.02

Data collection				
Diffractometer	Oxford Diffraction Xcalibur, Eos	Oxford Diffraction Xcalibur, Eos	Oxford Diffraction Xcalibur, Eos	Oxford Diffraction Xcalibur, Eos
Absorption correction	Multi-scan (*CrysAlis PRO*; Rigaku OD, 2020 ▸)	Multi-scan (*CrysAlis PRO*; Rigaku OD, 2020 ▸)	Multi-scan (*CrysAlis PRO*; Rigaku OD, 2020 ▸)	Multi-scan (*CrysAlis PRO*; Rigaku OD, 2020 ▸)
*T*_min_, *T*_max_	0.696, 1.000	0.497, 1.000	0.265, 1.000	0.328, 1.000
No. of measured, independent and observed [*I* > 2σ(*I*)] reflections	7091, 7091, 5430	5734, 5734, 3079	82101, 5671, 4933	38517, 2983, 2244
*R*_int_	–	–	0.057	0.079
θ values (°)	θ_max_ = 30.0, θ_min_ = 3.2	θ_max_ = 28.3, θ_min_ = 2.2	θ_max_ = 31.0, θ_min_ = 2.6	θ_max_ = 28.3, θ_min_ = 2.5
(sin θ/λ)_max_ (Å^−1^)	0.703	0.667	0.725	0.667

Refinement				
*R*[*F*^2^ > 2σ(*F*^2^)], *wR*(*F*^2^), *S*	0.022, 0.036, 0.85	0.039, 0.066, 0.78	0.030, 0.061, 1.05	0.031, 0.052, 1.04
No. of reflections	7091	5734	5671	2983
No. of parameters	228	238	191	117
No. of restraints	1	138	1	0
H-atom treatment	H atoms treated by a mixture of independent and constrained refinement	H-atom parameters constrained	H atoms treated by a mixture of independent and constrained refinement	H atoms treated by a mixture of independent and constrained refinement
Δρ_max_, Δρ_min_ (e Å^−3^)	1.33, −0.99	1.65, −1.21	2.27, −1.89	1.05, −0.99

**Table:** 

	**5**	**6**	**7**
Crystal data			
Chemical formula	[AuBr_2_(C_6_H_7_N)_2_][AuBr_4_]·CH_3_NO_2_	(C_6_H_8_N)[AuBr_4_]	(C_7_H_10_N)[AuBr_4_]
*M*_r_	1120.69	610.74	624.77
Crystal system, space group	Triclinic, *P* 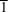	Triclinic, *P* 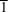	Orthorhombic, *P*2_1_2_1_2_1_
Temperature (K)	101	100	100
*a*, *b*, *c* (Å)	7.5336 (4), 12.49946 (10), 12.74241 (10)	7.5701 (3), 9.5159 (5), 9.5653 (5)	8.8797 (3), 9.4081 (4), 15.5202 (5)
α, β, γ (°)	84.400 (6), 89.908 (5), 86.012 (5)	112.616 (5), 104.788 (4), 96.401 (4)	90, 90, 90
*V* (Å^3^)	1191.26 (7)	597.79 (6)	1296.57 (8)
*Z*	2	2	4
Radiation type	Mo *K*α	Mo *K*α	Mo *K*α
μ (mm^−1^)	22.38	25.63	23.63
Crystal size (mm)	0.20 × 0.08 × 0.01	0.18 × 0.10 × 0.01	0.25 × 0.25 × 0.07

Data collection			
Diffractometer	Oxford Diffraction Xcalibur, Eos	Oxford Diffraction Xcalibur, Eos	Oxford Diffraction Xcalibur, Eos
Absorption correction	Multi-scan (*CrysAlis PRO*; Rigaku OD, 2020 ▸)	Multi-scan (*CrysAlis PRO*; Rigaku OD, 2020 ▸)	Multi-scan (*CrysAlis PRO*; Rigaku OD, 2020 ▸)
*T*_min_, *T*_max_	0.140, 1.000	0.298, 1.000	0.212, 1.000
No. of measured, independent and observed [*I* > 2σ(*I*)] reflections	6279, 6279, 5020	41754, 3555, 3064	33591, 3759, 3574
*R*_int_	0.104	0.075	0.066
θ values (°)	θ_max_ = 28.3, θ_min_ = 2.2	θ_max_ = 30.9, θ_min_ = 2.4	θ_max_ = 30.0, θ_min_ = 2.5
(sin θ/λ)_max_ (Å^−1^)	0.667	0.722	0.704

Refinement			
*R*[*F*^2^ > 2σ(*F*^2^)], *wR*(*F*^2^), *S*	0.038, 0.063, 0.94	0.034, 0.092, 1.08	0.024, 0.040, 1.04
No. of reflections	6279	3555	3759
No. of parameters	222	117	125
No. of restraints	84	0	0
H-atom treatment	H-atom parameters constrained	H atoms treated by a mixture of independent and constrained refinement	H atoms treated by a mixture of independent and constrained refinement
Δρ_max_, Δρ_min_ (e Å^−3^)	2.43, −1.74	2.05, −1.97	1.67, −1.19
Absolute structure	–	–	Flack *x* determined using 1420 quotients [(*I*^+^)−(*I*^−^)]/[(*I*^+^)+(*I*^−^)] (Parsons *et al.*, 2013 ▸)
Absolute structure parameter	–	–	−0.024 (6)

Computer programs: *CrysAlis PRO* (Rigaku OD, 2020 ▸), *SHELXS97* (Sheldrick, 2008 ▸), *SHELXL2019/3* (Sheldrick, 2015 ▸), *XP* (Bruker, 1998 ▸) and *publCIF* (Westrip, 2010 ▸).

## supplementary crystallographic information

### 2-Picolinium tetrachloridoaurate(III) (1) Crystal data

**Table:**

(C_6_H_8_N)[AuCl_4_]	*Z* = 4
*M**_r_* = 432.90	*F*(000) = 792
Triclinic, *P*1	*D*_x_ = 2.636 Mg m^−^^3^
*a* = 8.0764 (3) Å	Mo *K*α radiation, λ = 0.71073 Å
*b* = 9.0839 (3) Å	Cell parameters from 19407 reflections
*c* = 15.3667 (6) Å	θ = 3.3–29.7°
α = 87.792 (3)°	µ = 14.41 mm^−^^1^
β = 76.132 (3)°	*T* = 100 K
γ = 85.391 (3)°	Block, yellow
*V* = 1090.77 (7) Å^3^	0.10 × 0.08 × 0.03 mm

### 2-Picolinium tetrachloridoaurate(III) (1) Data collection

**Table:**

Oxford Diffraction Xcalibur, Eos diffractometer	7091 measured reflections
Radiation source: Enhance (Mo) X-ray Source	7091 independent reflections
Graphite monochromator	5430 reflections with *I* > 2σ(*I*)
Detector resolution: 16.1419 pixels mm^-1^	θ_max_ = 30.0°, θ_min_ = 3.2°
ω scan	*h* = −11→11
Absorption correction: multi-scan (CrysAlisPro; Rigaku OD, 2020)	*k* = −12→12
*T*_min_ = 0.696, *T*_max_ = 1.000	*l* = −21→21

### 2-Picolinium tetrachloridoaurate(III) (1) Refinement

**Table:**

Refinement on *F*^2^	Primary atom site location: structure-invariant direct methods
Least-squares matrix: full	Secondary atom site location: difference Fourier map
*R*[*F*^2^ > 2σ(*F*^2^)] = 0.022	Hydrogen site location: mixed
*wR*(*F*^2^) = 0.036	H atoms treated by a mixture of independent and constrained refinement
*S* = 0.85	*w* = 1/[σ^2^(*F*_o_^2^) + (0.0123*P*)^2^] where *P* = (*F*_o_^2^ + 2*F*_c_^2^)/3
7091 reflections	(Δ/σ)_max_ = 0.001
228 parameters	Δρ_max_ = 1.33 e Å^−^^3^
1 restraint	Δρ_min_ = −0.99 e Å^−^^3^

### 2-Picolinium tetrachloridoaurate(III) (1) Fractional atomic coordinates and isotropic or equivalent isotropic displacement parameters (Å^2^)

**Table:**

	*x*	*y*	*z*	*U*_iso_*/*U*_eq_	
Au1	0.04499 (2)	0.25067 (2)	0.73413 (2)	0.01327 (4)	
Cl1	−0.11813 (13)	0.06827 (11)	0.71157 (7)	0.0209 (2)	
Cl2	−0.05960 (13)	0.23114 (11)	0.88505 (7)	0.0181 (2)	
Cl3	0.21633 (13)	0.42704 (11)	0.75552 (7)	0.0210 (2)	
Cl4	0.14799 (14)	0.27269 (12)	0.58268 (7)	0.0234 (2)	
Au2	0.53370 (2)	0.29444 (2)	0.23295 (2)	0.01298 (4)	
Cl5	0.62742 (14)	0.28945 (11)	0.08054 (7)	0.0222 (2)	
Cl6	0.72287 (14)	0.09876 (11)	0.24628 (7)	0.0211 (2)	
Cl7	0.44219 (13)	0.29956 (11)	0.38481 (7)	0.0201 (2)	
Cl8	0.34721 (14)	0.49184 (11)	0.21892 (7)	0.0215 (2)	
N11	0.1874 (5)	−0.1127 (4)	0.5398 (3)	0.0211 (8)	
H01	0.125 (5)	−0.050 (4)	0.580 (3)	0.032 (14)*	
C12	0.1543 (5)	−0.2569 (4)	0.5481 (3)	0.0168 (9)	
C13	0.2573 (5)	−0.3512 (5)	0.4866 (3)	0.0211 (10)	
H13	0.237539	−0.453190	0.489956	0.025*	
C14	0.3881 (6)	−0.3010 (5)	0.4203 (3)	0.0265 (11)	
H14	0.458982	−0.368074	0.378915	0.032*	
C15	0.4161 (6)	−0.1524 (5)	0.4143 (3)	0.0234 (10)	
H15	0.505375	−0.116002	0.368413	0.028*	
C16	0.3138 (6)	−0.0590 (5)	0.4750 (3)	0.0236 (11)	
H16	0.331091	0.043435	0.471837	0.028*	
C17	0.0118 (5)	−0.3040 (5)	0.6223 (3)	0.0232 (10)	
H17A	0.032199	−0.277713	0.679882	0.035*	
H17B	0.005955	−0.411185	0.620687	0.035*	
H17C	−0.096390	−0.254147	0.615041	0.035*	
N21	0.6864 (5)	0.6566 (4)	0.0389 (2)	0.0179 (8)	
H02	0.624 (4)	0.595 (4)	0.075 (2)	0.011 (11)*	
C22	0.6517 (5)	0.8027 (4)	0.0540 (3)	0.0171 (10)	
C23	0.7491 (5)	0.8982 (4)	−0.0039 (3)	0.0195 (10)	
H23	0.728499	1.001657	0.004848	0.023*	
C24	0.8766 (6)	0.8453 (5)	−0.0747 (3)	0.0227 (10)	
H24	0.943633	0.912163	−0.114545	0.027*	
C25	0.9069 (6)	0.6942 (5)	−0.0878 (3)	0.0247 (11)	
H25	0.994546	0.656741	−0.136395	0.030*	
C26	0.8092 (6)	0.6009 (5)	−0.0299 (3)	0.0228 (10)	
H26	0.827441	0.497117	−0.037766	0.027*	
C27	0.5107 (5)	0.8485 (5)	0.1318 (3)	0.0238 (10)	
H27A	0.533940	0.802637	0.186868	0.036*	
H27B	0.402597	0.816864	0.123302	0.036*	
H27C	0.502637	0.956237	0.136610	0.036*	

### 2-Picolinium tetrachloridoaurate(III) (1) Atomic displacement parameters (Å^2^)

**Table:**

	*U*^11^	*U*^22^	*U*^33^	*U*^12^	*U*^13^	*U*^23^
Au1	0.01229 (9)	0.01474 (9)	0.01282 (9)	0.00096 (6)	−0.00351 (7)	−0.00171 (6)
Cl1	0.0191 (6)	0.0226 (6)	0.0230 (6)	−0.0042 (4)	−0.0069 (5)	−0.0050 (4)
Cl2	0.0216 (6)	0.0190 (5)	0.0131 (5)	−0.0018 (4)	−0.0023 (4)	−0.0011 (4)
Cl3	0.0201 (6)	0.0214 (5)	0.0225 (6)	−0.0067 (4)	−0.0049 (5)	−0.0018 (4)
Cl4	0.0271 (6)	0.0284 (6)	0.0132 (5)	−0.0020 (5)	−0.0021 (5)	−0.0008 (4)
Au2	0.01309 (9)	0.01294 (9)	0.01346 (9)	−0.00177 (7)	−0.00400 (7)	0.00014 (6)
Cl5	0.0267 (6)	0.0250 (6)	0.0144 (5)	−0.0019 (5)	−0.0037 (5)	−0.0002 (4)
Cl6	0.0194 (6)	0.0206 (6)	0.0220 (6)	0.0060 (4)	−0.0048 (4)	−0.0005 (4)
Cl7	0.0213 (6)	0.0235 (6)	0.0143 (5)	−0.0004 (4)	−0.0022 (4)	−0.0002 (4)
Cl8	0.0220 (6)	0.0174 (5)	0.0267 (6)	0.0047 (4)	−0.0105 (5)	−0.0013 (4)
N11	0.023 (2)	0.018 (2)	0.022 (2)	0.0032 (17)	−0.0060 (18)	−0.0051 (17)
C12	0.017 (2)	0.018 (2)	0.019 (2)	−0.0009 (18)	−0.0100 (19)	0.0006 (18)
C13	0.020 (2)	0.016 (2)	0.028 (3)	−0.0015 (19)	−0.008 (2)	−0.0043 (19)
C14	0.024 (3)	0.034 (3)	0.024 (3)	0.003 (2)	−0.010 (2)	−0.012 (2)
C15	0.019 (3)	0.035 (3)	0.017 (2)	−0.007 (2)	−0.006 (2)	0.006 (2)
C16	0.025 (3)	0.021 (2)	0.026 (3)	−0.006 (2)	−0.011 (2)	0.008 (2)
C17	0.021 (2)	0.021 (2)	0.026 (3)	−0.0012 (19)	−0.004 (2)	0.0017 (19)
N21	0.022 (2)	0.0140 (19)	0.020 (2)	−0.0062 (16)	−0.0083 (17)	0.0049 (15)
C22	0.020 (2)	0.017 (2)	0.018 (2)	0.0010 (18)	−0.012 (2)	−0.0006 (18)
C23	0.023 (3)	0.014 (2)	0.023 (2)	−0.0023 (18)	−0.007 (2)	0.0015 (18)
C24	0.022 (3)	0.023 (3)	0.022 (3)	−0.004 (2)	−0.004 (2)	0.0042 (19)
C25	0.024 (3)	0.029 (3)	0.020 (3)	0.005 (2)	−0.003 (2)	−0.006 (2)
C26	0.032 (3)	0.017 (2)	0.021 (2)	0.003 (2)	−0.010 (2)	−0.0057 (19)
C27	0.020 (2)	0.025 (2)	0.026 (3)	−0.0018 (19)	−0.004 (2)	−0.0003 (19)

### 2-Picolinium tetrachloridoaurate(III) (1) Geometric parameters (Å, º)

**Table:**

Au1—Cl2	2.2752 (10)	C16—H16	0.9500
Au1—Cl3	2.2814 (10)	C17—H17A	0.9800
Au1—Cl4	2.2827 (10)	C17—H17B	0.9800
Au1—Cl1	2.2837 (10)	C17—H17C	0.9800
Au2—Cl7	2.2737 (10)	N21—C26	1.347 (5)
Au2—Cl5	2.2832 (11)	N21—C22	1.351 (5)
Au2—Cl6	2.2851 (10)	N21—H02	0.87 (3)
Au2—Cl8	2.2852 (10)	C22—C23	1.371 (5)
N11—C16	1.351 (5)	C22—C27	1.486 (6)
N11—C12	1.353 (5)	C23—C24	1.378 (6)
N11—H01	0.88 (3)	C23—H23	0.9500
C12—C13	1.375 (6)	C24—C25	1.388 (6)
C12—C17	1.490 (6)	C24—H24	0.9500
C13—C14	1.373 (6)	C25—C26	1.359 (6)
C13—H13	0.9500	C25—H25	0.9500
C14—C15	1.383 (6)	C26—H26	0.9500
C14—H14	0.9500	C27—H27A	0.9800
C15—C16	1.361 (6)	C27—H27B	0.9800
C15—H15	0.9500	C27—H27C	0.9800
			
Cl2—Au1—Cl3	89.73 (4)	C12—C17—H17A	109.5
Cl2—Au1—Cl4	179.35 (4)	C12—C17—H17B	109.5
Cl3—Au1—Cl4	90.13 (4)	H17A—C17—H17B	109.5
Cl2—Au1—Cl1	90.81 (4)	C12—C17—H17C	109.5
Cl3—Au1—Cl1	177.95 (4)	H17A—C17—H17C	109.5
Cl4—Au1—Cl1	89.35 (4)	H17B—C17—H17C	109.5
Cl7—Au2—Cl5	179.62 (4)	C26—N21—C22	123.8 (4)
Cl7—Au2—Cl6	89.60 (4)	C26—N21—H02	118 (3)
Cl5—Au2—Cl6	90.19 (4)	C22—N21—H02	118 (3)
Cl7—Au2—Cl8	90.69 (4)	N21—C22—C23	117.5 (4)
Cl5—Au2—Cl8	89.52 (4)	N21—C22—C27	117.9 (4)
Cl6—Au2—Cl8	179.31 (4)	C23—C22—C27	124.6 (4)
C16—N11—C12	123.5 (4)	C22—C23—C24	120.5 (4)
C16—N11—H01	118 (3)	C22—C23—H23	119.8
C12—N11—H01	119 (3)	C24—C23—H23	119.8
N11—C12—C13	116.8 (4)	C23—C24—C25	120.0 (4)
N11—C12—C17	119.0 (4)	C23—C24—H24	120.0
C13—C12—C17	124.2 (4)	C25—C24—H24	120.0
C14—C13—C12	121.4 (4)	C26—C25—C24	118.9 (4)
C14—C13—H13	119.3	C26—C25—H25	120.5
C12—C13—H13	119.3	C24—C25—H25	120.5
C13—C14—C15	119.6 (4)	N21—C26—C25	119.5 (4)
C13—C14—H14	120.2	N21—C26—H26	120.3
C15—C14—H14	120.2	C25—C26—H26	120.3
C16—C15—C14	119.0 (4)	C22—C27—H27A	109.5
C16—C15—H15	120.5	C22—C27—H27B	109.5
C14—C15—H15	120.5	H27A—C27—H27B	109.5
N11—C16—C15	119.7 (4)	C22—C27—H27C	109.5
N11—C16—H16	120.1	H27A—C27—H27C	109.5
C15—C16—H16	120.1	H27B—C27—H27C	109.5
			
C16—N11—C12—C13	0.3 (6)	C26—N21—C22—C23	−0.6 (6)
C16—N11—C12—C17	179.9 (4)	C26—N21—C22—C27	179.2 (4)
N11—C12—C13—C14	0.4 (6)	N21—C22—C23—C24	0.2 (6)
C17—C12—C13—C14	−179.1 (4)	C27—C22—C23—C24	−179.5 (4)
C12—C13—C14—C15	−0.9 (6)	C22—C23—C24—C25	0.1 (6)
C13—C14—C15—C16	0.6 (6)	C23—C24—C25—C26	0.0 (6)
C12—N11—C16—C15	−0.5 (6)	C22—N21—C26—C25	0.7 (6)
C14—C15—C16—N11	0.0 (6)	C24—C25—C26—N21	−0.4 (6)

### 2-Picolinium tetrachloridoaurate(III) (1) Hydrogen-bond geometry (Å, º)

**Table:**

*D*—H···*A*	*D*—H	H···*A*	*D*···*A*	*D*—H···*A*
N11—H01···Cl1	0.88 (3)	2.66 (3)	3.510 (4)	163 (4)
N11—H01···Cl4	0.88 (3)	2.96 (4)	3.562 (4)	127 (3)
N21—H02···Cl5	0.87 (3)	2.77 (3)	3.421 (3)	132 (3)
N21—H02···Cl8	0.87 (3)	2.93 (3)	3.756 (4)	159 (3)
C13—H13···Cl4^i^	0.95	2.88	3.756 (4)	154
C13—H13···Cl7^i^	0.95	2.96	3.639 (4)	129
C16—H16···Cl4	0.95	2.83	3.505 (5)	129
C16—H16···Cl7	0.95	2.74	3.621 (4)	154
C17—H17*C*···Cl7^ii^	0.98	2.86	3.691 (4)	144
C23—H23···Cl5^iii^	0.95	2.88	3.803 (4)	164
C25—H25···Cl3^iv^	0.95	2.93	3.811 (5)	156
C26—H26···Cl2^iv^	0.95	2.72	3.635 (4)	161
C26—H26···Cl5	0.95	2.88	3.484 (5)	123
C27—H27*B*···Cl2^v^	0.98	2.87	3.841 (4)	170

Symmetry codes: (i) *x*, *y*−1, *z*; (ii) −*x*, −*y*, −*z*+1; (iii) *x*, *y*+1, *z*; (iv) *x*+1, *y*, *z*−1; (v) −*x*, −*y*+1, −*z*+1.

### 2-Picolinium tetrabromidoaurate(III) (2) Crystal data

**Table:**

(C_6_H_8_N)[AuBr_4_]	*Z* = 4
*M**_r_* = 610.74	*F*(000) = 1080
Triclinic, *P*1	*D*_x_ = 3.385 Mg m^−^^3^
*a* = 9.9208 (5) Å	Mo *K*α radiation, λ = 0.71073 Å
*b* = 11.9072 (6) Å	Cell parameters from 9800 reflections
*c* = 12.2765 (7) Å	θ = 2.6–28.4°
α = 65.423 (5)°	µ = 25.57 mm^−^^1^
β = 70.506 (5)°	*T* = 100 K
γ = 68.459 (5)°	Plate, red
*V* = 1198.31 (13) Å^3^	0.12 × 0.08 × 0.01 mm

### 2-Picolinium tetrabromidoaurate(III) (2) Data collection

**Table:**

Oxford Diffraction Xcalibur, Eos diffractometer	5734 measured reflections
Radiation source: Enhance (Mo) X-ray Source	5734 independent reflections
Graphite monochromator	3079 reflections with *I* > 2σ(*I*)
Detector resolution: 16.1419 pixels mm^-1^	θ_max_ = 28.3°, θ_min_ = 2.2°
ω scans	*h* = −13→13
Absorption correction: multi-scan (CrysAlisPro; Rigaku OD, 2020)	*k* = −15→15
*T*_min_ = 0.497, *T*_max_ = 1.000	*l* = −16→16

### 2-Picolinium tetrabromidoaurate(III) (2) Refinement

**Table:**

Refinement on *F*^2^	Primary atom site location: structure-invariant direct methods
Least-squares matrix: full	Secondary atom site location: difference Fourier map
*R*[*F*^2^ > 2σ(*F*^2^)] = 0.039	Hydrogen site location: inferred from neighbouring sites
*wR*(*F*^2^) = 0.066	H-atom parameters constrained
*S* = 0.78	*w* = 1/[σ^2^(*F*_o_^2^) + (0.0245*P*)^2^] where *P* = (*F*_o_^2^ + 2*F*_c_^2^)/3
5734 reflections	(Δ/σ)_max_ = 0.001
238 parameters	Δρ_max_ = 1.65 e Å^−^^3^
138 restraints	Δρ_min_ = −1.20 e Å^−^^3^

### 2-Picolinium tetrabromidoaurate(III) (2) Fractional atomic coordinates and isotropic or equivalent isotropic displacement parameters (Å^2^)

**Table:**

	*x*	*y*	*z*	*U*_iso_*/*U*_eq_	Occ. (<1)
Au1	0.24893 (4)	0.73071 (4)	0.73650 (4)	0.01420 (10)	
Au2	0.23715 (5)	0.74034 (4)	0.23848 (4)	0.01813 (11)	
Br1	0.45211 (11)	0.53835 (9)	0.74002 (10)	0.0225 (3)	
Br2	0.41979 (11)	0.86153 (10)	0.61615 (10)	0.0213 (3)	
Br3	0.04624 (10)	0.92151 (9)	0.73970 (10)	0.0234 (3)	
Br4	0.07638 (11)	0.59919 (10)	0.85305 (11)	0.0223 (3)	
Br5	0.06760 (11)	0.60600 (10)	0.35068 (11)	0.0244 (3)	
Br6	0.24442 (14)	0.74831 (11)	0.43021 (11)	0.0380 (3)	
Br7	0.41391 (12)	0.86653 (11)	0.12801 (12)	0.0304 (3)	
Br8	0.21872 (11)	0.73903 (11)	0.04725 (10)	0.0279 (3)	
N11	0.2281 (8)	0.3143 (8)	0.7980 (8)	0.024 (2)	
H01	0.217453	0.393332	0.791579	0.029*	
C12	0.2942 (11)	0.2807 (11)	0.6987 (10)	0.024 (3)	
C13	0.3099 (11)	0.1544 (9)	0.7086 (11)	0.026 (3)	
H13	0.355947	0.126238	0.639638	0.032*	
C14	0.2571 (11)	0.0736 (10)	0.8198 (11)	0.030 (3)	
H14	0.269790	−0.012329	0.827954	0.036*	
C15	0.1863 (11)	0.1123 (11)	0.9206 (11)	0.029 (3)	
H15	0.145934	0.055611	0.995838	0.034*	
C16	0.1754 (11)	0.2336 (11)	0.9101 (11)	0.028 (3)	
H16	0.132115	0.261779	0.979082	0.034*	
C17	0.3411 (11)	0.3798 (10)	0.5833 (10)	0.029 (3)	
H17A	0.422009	0.403015	0.590644	0.044*	
H17B	0.256987	0.455836	0.567583	0.044*	
H17C	0.375349	0.346268	0.515320	0.044*	
N21	0.2277 (14)	0.3355 (10)	0.2789 (10)	0.021 (3)	0.811 (11)
H02	0.212366	0.416875	0.267273	0.025*	0.811 (11)
C22	0.3108 (13)	0.2926 (10)	0.1846 (10)	0.020 (3)	0.811 (11)
C23	0.3347 (13)	0.1631 (10)	0.2041 (11)	0.018 (3)	0.811 (11)
H23	0.391882	0.128362	0.140566	0.021*	0.811 (11)
C24	0.2743 (13)	0.0860 (12)	0.3170 (11)	0.025 (3)	0.811 (11)
H24	0.291916	−0.002368	0.330825	0.030*	0.811 (11)
C25	0.1896 (15)	0.1346 (12)	0.4091 (12)	0.029 (3)	0.811 (11)
H25	0.147228	0.080813	0.485535	0.035*	0.811 (11)
C26	0.1664 (12)	0.2623 (11)	0.3900 (11)	0.024 (3)	0.811 (11)
H26	0.109024	0.298001	0.452936	0.028*	0.811 (11)
C27	0.3657 (14)	0.3817 (11)	0.0701 (12)	0.022 (3)	0.811 (11)
H27A	0.407052	0.435266	0.085961	0.033*	0.811 (11)
H27B	0.284258	0.436219	0.027675	0.033*	0.811 (11)
H27C	0.443272	0.334401	0.018778	0.033*	0.811 (11)
N21'	0.237 (5)	0.341 (3)	0.259 (3)	0.022 (9)*	0.189 (11)
H21'	0.192848	0.395390	0.299304	0.026*	0.189 (11)
C22'	0.233 (4)	0.213 (3)	0.317 (2)	0.030 (16)*	0.189 (11)
C23'	0.302 (4)	0.127 (2)	0.254 (3)	0.016 (7)*	0.189 (11)
H23'	0.299368	0.039981	0.293698	0.020*	0.189 (11)
C24'	0.375 (4)	0.168 (3)	0.132 (3)	0.015 (8)*	0.189 (11)
H24'	0.422263	0.108706	0.088376	0.018*	0.189 (11)
C25'	0.379 (3)	0.295 (3)	0.073 (2)	0.010 (11)*	0.189 (11)
H25'	0.428709	0.322950	−0.009882	0.012*	0.189 (11)
C26'	0.310 (4)	0.381 (2)	0.137 (3)	0.009 (10)*	0.189 (11)
H26'	0.312261	0.468470	0.097181	0.011*	0.189 (11)
C27'	0.151 (5)	0.183 (4)	0.442 (3)	0.013 (11)*	0.189 (11)
H27D	0.095052	0.122859	0.457159	0.020*	0.189 (11)
H27E	0.081266	0.261563	0.455908	0.020*	0.189 (11)
H27F	0.219220	0.143833	0.497500	0.020*	0.189 (11)

### 2-Picolinium tetrabromidoaurate(III) (2) Atomic displacement parameters (Å^2^)

**Table:**

	*U*^11^	*U*^22^	*U*^33^	*U*^12^	*U*^13^	*U*^23^
Au1	0.0133 (2)	0.0141 (2)	0.0155 (2)	−0.00532 (17)	−0.00297 (17)	−0.00378 (16)
Au2	0.0175 (2)	0.0141 (2)	0.0250 (2)	−0.00302 (17)	−0.00964 (18)	−0.00580 (18)
Br1	0.0174 (5)	0.0175 (5)	0.0279 (6)	−0.0036 (4)	−0.0026 (5)	−0.0057 (5)
Br2	0.0177 (5)	0.0201 (6)	0.0258 (7)	−0.0108 (5)	−0.0014 (5)	−0.0049 (5)
Br3	0.0159 (5)	0.0180 (5)	0.0320 (6)	−0.0034 (4)	−0.0049 (5)	−0.0055 (5)
Br4	0.0181 (6)	0.0190 (6)	0.0287 (7)	−0.0085 (5)	0.0010 (5)	−0.0086 (5)
Br5	0.0207 (6)	0.0242 (6)	0.0289 (7)	−0.0090 (5)	−0.0014 (5)	−0.0101 (5)
Br6	0.0582 (8)	0.0353 (7)	0.0310 (7)	−0.0148 (6)	−0.0220 (6)	−0.0092 (6)
Br7	0.0283 (6)	0.0220 (6)	0.0439 (8)	−0.0118 (5)	−0.0177 (6)	−0.0018 (6)
Br8	0.0261 (6)	0.0383 (7)	0.0263 (6)	−0.0123 (5)	−0.0065 (5)	−0.0136 (5)
N11	0.021 (5)	0.029 (5)	0.032 (6)	0.000 (4)	−0.016 (4)	−0.016 (5)
C12	0.012 (5)	0.033 (7)	0.030 (7)	−0.010 (5)	−0.007 (5)	−0.008 (6)
C13	0.027 (6)	0.012 (5)	0.043 (8)	−0.002 (5)	−0.018 (6)	−0.006 (5)
C14	0.023 (6)	0.020 (6)	0.037 (7)	−0.004 (5)	−0.007 (6)	−0.001 (6)
C15	0.022 (6)	0.037 (7)	0.024 (7)	−0.013 (5)	−0.007 (5)	−0.002 (6)
C16	0.019 (6)	0.038 (7)	0.028 (7)	−0.007 (5)	−0.003 (5)	−0.013 (6)
C17	0.037 (7)	0.019 (6)	0.027 (7)	−0.013 (5)	0.001 (6)	−0.006 (5)
N21	0.022 (4)	0.019 (4)	0.023 (4)	−0.006 (3)	−0.007 (3)	−0.006 (3)
C22	0.022 (5)	0.020 (4)	0.022 (4)	−0.006 (3)	−0.009 (3)	−0.006 (3)
C23	0.018 (4)	0.020 (4)	0.022 (4)	−0.007 (3)	−0.011 (3)	−0.007 (3)
C24	0.023 (5)	0.025 (4)	0.026 (4)	−0.006 (3)	−0.009 (3)	−0.004 (3)
C25	0.026 (5)	0.031 (4)	0.028 (5)	−0.011 (4)	−0.005 (4)	−0.006 (4)
C26	0.018 (5)	0.028 (4)	0.025 (4)	−0.007 (3)	−0.007 (3)	−0.005 (3)
C27	0.026 (6)	0.019 (5)	0.025 (5)	−0.008 (5)	−0.003 (5)	−0.012 (4)

### 2-Picolinium tetrabromidoaurate(III) (2) Geometric parameters (Å, º)

**Table:**

Au1—Br2	2.4157 (12)	C22—C27	1.439 (15)
Au1—Br3	2.4235 (11)	C23—C24	1.383 (13)
Au1—Br1	2.4283 (11)	C23—H23	0.9500
Au1—Br4	2.4290 (12)	C24—C25	1.368 (13)
Au2—Br7	2.4135 (13)	C24—H24	0.9500
Au2—Br6	2.4206 (13)	C25—C26	1.380 (13)
Au2—Br8	2.4215 (12)	C25—H25	0.9500
Au2—Br5	2.4253 (12)	C26—H26	0.9500
N11—C12	1.325 (13)	C27—H27A	0.9800
N11—C16	1.371 (13)	C27—H27B	0.9800
N11—H01	0.8800	C27—H27C	0.9800
C12—C13	1.410 (14)	N21'—C22'	1.3900
C12—C17	1.480 (14)	N21'—C26'	1.3900
C13—C14	1.365 (14)	N21'—H21'	0.8800
C13—H13	0.9500	C22'—C23'	1.3900
C14—C15	1.375 (15)	C22'—C27'	1.43 (2)
C14—H14	0.9500	C23'—C24'	1.3900
C15—C16	1.362 (14)	C23'—H23'	0.9500
C15—H15	0.9500	C24'—C25'	1.3900
C16—H16	0.9500	C24'—H24'	0.9500
C17—H17A	0.9800	C25'—C26'	1.3900
C17—H17B	0.9800	C25'—H25'	0.9500
C17—H17C	0.9800	C26'—H26'	0.9500
N21—C22	1.350 (12)	C27'—H27D	0.9800
N21—C26	1.355 (13)	C27'—H27E	0.9800
N21—H02	0.8800	C27'—H27F	0.9800
C22—C23	1.398 (12)		
			
Br2—Au1—Br3	90.20 (4)	C24—C23—C22	119.3 (11)
Br2—Au1—Br1	90.21 (4)	C24—C23—H23	120.3
Br3—Au1—Br1	178.16 (5)	C22—C23—H23	120.3
Br2—Au1—Br4	178.64 (5)	C25—C24—C23	121.2 (11)
Br3—Au1—Br4	89.88 (4)	C25—C24—H24	119.4
Br1—Au1—Br4	89.75 (4)	C23—C24—H24	119.4
Br7—Au2—Br6	90.58 (5)	C24—C25—C26	119.5 (12)
Br7—Au2—Br8	90.20 (5)	C24—C25—H25	120.3
Br6—Au2—Br8	177.40 (5)	C26—C25—H25	120.3
Br7—Au2—Br5	177.75 (5)	N21—C26—C25	118.0 (12)
Br6—Au2—Br5	89.33 (4)	N21—C26—H26	121.0
Br8—Au2—Br5	89.98 (4)	C25—C26—H26	121.0
C12—N11—C16	124.1 (10)	C22—C27—H27A	109.5
C12—N11—H01	117.9	C22—C27—H27B	109.5
C16—N11—H01	117.9	H27A—C27—H27B	109.5
N11—C12—C13	118.0 (11)	C22—C27—H27C	109.5
N11—C12—C17	117.8 (10)	H27A—C27—H27C	109.5
C13—C12—C17	124.2 (11)	H27B—C27—H27C	109.5
C14—C13—C12	118.2 (11)	C22'—N21'—C26'	120.0
C14—C13—H13	120.9	C22'—N21'—H21'	120.0
C12—C13—H13	120.9	C26'—N21'—H21'	120.0
C13—C14—C15	122.4 (11)	N21'—C22'—C23'	120.0
C13—C14—H14	118.8	N21'—C22'—C27'	115 (2)
C15—C14—H14	118.8	C23'—C22'—C27'	125 (2)
C16—C15—C14	118.5 (11)	C22'—C23'—C24'	120.0
C16—C15—H15	120.7	C22'—C23'—H23'	120.0
C14—C15—H15	120.7	C24'—C23'—H23'	120.0
C15—C16—N11	118.7 (11)	C25'—C24'—C23'	120.0
C15—C16—H16	120.6	C25'—C24'—H24'	120.0
N11—C16—H16	120.6	C23'—C24'—H24'	120.0
C12—C17—H17A	109.5	C24'—C25'—C26'	120.0
C12—C17—H17B	109.5	C24'—C25'—H25'	120.0
H17A—C17—H17B	109.5	C26'—C25'—H25'	120.0
C12—C17—H17C	109.5	C25'—C26'—N21'	120.0
H17A—C17—H17C	109.5	C25'—C26'—H26'	120.0
H17B—C17—H17C	109.5	N21'—C26'—H26'	120.0
C22—N21—C26	124.9 (11)	C22'—C27'—H27D	109.5
C22—N21—H02	117.6	C22'—C27'—H27E	109.5
C26—N21—H02	117.6	H27D—C27'—H27E	109.5
N21—C22—C23	117.1 (10)	C22'—C27'—H27F	109.5
N21—C22—C27	119.1 (11)	H27D—C27'—H27F	109.5
C23—C22—C27	123.8 (11)	H27E—C27'—H27F	109.5
			
C16—N11—C12—C13	0.7 (14)	C22—C23—C24—C25	1.1 (18)
C16—N11—C12—C17	178.2 (9)	C23—C24—C25—C26	−1 (2)
N11—C12—C13—C14	−0.4 (14)	C22—N21—C26—C25	0 (2)
C17—C12—C13—C14	−177.7 (9)	C24—C25—C26—N21	0.8 (19)
C12—C13—C14—C15	1.8 (16)	C26'—N21'—C22'—C23'	0.0
C13—C14—C15—C16	−3.4 (16)	C26'—N21'—C22'—C27'	−178 (4)
C14—C15—C16—N11	3.5 (15)	N21'—C22'—C23'—C24'	0.0
C12—N11—C16—C15	−2.3 (15)	C27'—C22'—C23'—C24'	178 (4)
C26—N21—C22—C23	0 (2)	C22'—C23'—C24'—C25'	0.0
C26—N21—C22—C27	178.2 (12)	C23'—C24'—C25'—C26'	0.0
N21—C22—C23—C24	−0.4 (17)	C24'—C25'—C26'—N21'	0.0
C27—C22—C23—C24	−178.6 (12)	C22'—N21'—C26'—C25'	0.0

### 2-Picolinium tetrabromidoaurate(III) (2) Hydrogen-bond geometry (Å, º)

**Table:**

*D*—H···*A*	*D*—H	H···*A*	*D*···*A*	*D*—H···*A*
N11—H01···Br4	0.88	2.62	3.422 (9)	153
N21—H02···Br5	0.88	2.60	3.369 (10)	147
C13—H13···Br2^i^	0.95	3.10	3.783 (11)	131
C16—H16···Br4^ii^	0.95	2.96	3.870 (11)	162
C17—H17*A*···Br1	0.98	3.04	3.800 (11)	136
C17—H17*A*···Br6^iii^	0.98	3.11	3.796 (10)	128
C17—H17*C*···Br1^iii^	0.98	3.03	3.706 (11)	127
C23—H23···Br7^i^	0.95	3.11	3.765 (11)	128
C25—H25···Br3^i^	0.95	2.95	3.894 (13)	171
C26—H26···Br5^iv^	0.95	2.86	3.811 (13)	177
C27—H27*A*···Br1^iii^	0.98	3.08	3.940 (13)	147
C27—H27*B*···Br4^v^	0.98	3.03	3.994 (13)	169
C27—H27*C*···Br8^vi^	0.98	3.05	3.814 (13)	136

Symmetry codes: (i) *x*, *y*−1, *z*; (ii) −*x*, −*y*+1, −*z*+2; (iii) −*x*+1, −*y*+1, −*z*+1; (iv) −*x*, −*y*+1, −*z*+1; (v) *x*, *y*, *z*−1; (vi) −*x*+1, −*y*+1, −*z*.

### Bis(2-picolinium) tetrabromidoaurate(III) bromide (3) Crystal data

**Table:**

(C_6_H_8_N)_2_[AuBr_4_]Br	*Z* = 2
*M**_r_* = 784.78	*F*(000) = 712
Triclinic, *P*1	*D*_x_ = 2.762 Mg m^−^^3^
*a* = 8.7050 (2) Å	Mo *K*α radiation, λ = 0.71073 Å
*b* = 9.1257 (4) Å	Cell parameters from 20583 reflections
*c* = 13.8767 (6) Å	θ = 2.6–30.6°
α = 77.246 (4)°	µ = 18.37 mm^−^^1^
β = 80.023 (3)°	*T* = 100 K
γ = 61.718 (4)°	Block, red
*V* = 943.79 (7) Å^3^	0.20 × 0.12 × 0.06 mm

### Bis(2-picolinium) tetrabromidoaurate(III) bromide (3) Data collection

**Table:**

Oxford Diffraction Xcalibur, Eos diffractometer	5671 independent reflections
Radiation source: Enhance (Mo) X-ray Source	4933 reflections with *I* > 2σ(*I*)
Graphite monochromator	*R*_int_ = 0.057
Detector resolution: 16.1419 pixels mm^-1^	θ_max_ = 31.0°, θ_min_ = 2.6°
ω scan	*h* = −12→12
Absorption correction: multi-scan (CrysAlisPro; Rigaku OD, 2020)	*k* = −12→13
*T*_min_ = 0.265, *T*_max_ = 1.000	*l* = −19→19
82101 measured reflections	

### Bis(2-picolinium) tetrabromidoaurate(III) bromide (3) Refinement

**Table:**

Refinement on *F*^2^	Primary atom site location: structure-invariant direct methods
Least-squares matrix: full	Secondary atom site location: difference Fourier map
*R*[*F*^2^ > 2σ(*F*^2^)] = 0.030	Hydrogen site location: mixed
*wR*(*F*^2^) = 0.061	H atoms treated by a mixture of independent and constrained refinement
*S* = 1.05	*w* = 1/[σ^2^(*F*_o_^2^) + (0.0262*P*)^2^ + 2.4868*P*] where *P* = (*F*_o_^2^ + 2*F*_c_^2^)/3
5671 reflections	(Δ/σ)_max_ = 0.001
191 parameters	Δρ_max_ = 2.27 e Å^−^^3^
1 restraint	Δρ_min_ = −1.89 e Å^−^^3^

### Bis(2-picolinium) tetrabromidoaurate(III) bromide (3) Fractional atomic coordinates and isotropic or equivalent isotropic displacement parameters (Å^2^)

**Table:**

	*x*	*y*	*z*	*U*_iso_*/*U*_eq_	
Au1	0.11407 (2)	1.06950 (2)	0.20581 (2)	0.01417 (5)	
Br1	0.21739 (5)	0.84140 (5)	0.34354 (3)	0.02118 (9)	
Br2	0.35511 (5)	1.12787 (5)	0.21659 (3)	0.02096 (9)	
Br3	0.01181 (6)	1.30578 (5)	0.07197 (3)	0.02360 (9)	
Br4	−0.11065 (6)	0.99528 (5)	0.18662 (3)	0.02435 (10)	
Br5	0.24780 (5)	0.68861 (5)	0.61608 (3)	0.01923 (9)	
N11	−0.1116 (4)	0.7454 (4)	0.5435 (3)	0.0158 (7)	
H01	−0.029 (6)	0.739 (7)	0.565 (4)	0.042 (17)*	
C12	−0.1567 (5)	0.6209 (5)	0.5830 (3)	0.0156 (7)	
C13	−0.2966 (5)	0.6254 (5)	0.5453 (3)	0.0202 (8)	
H13	−0.333098	0.540384	0.570994	0.024*	
C14	−0.3828 (6)	0.7529 (6)	0.4707 (3)	0.0239 (9)	
H14	−0.478342	0.754875	0.445198	0.029*	
C15	−0.3318 (6)	0.8782 (5)	0.4326 (3)	0.0243 (9)	
H15	−0.391066	0.966497	0.381258	0.029*	
C16	−0.1926 (6)	0.8710 (5)	0.4713 (3)	0.0214 (8)	
H16	−0.154300	0.955054	0.446813	0.026*	
C17	−0.0550 (5)	0.4911 (5)	0.6635 (3)	0.0198 (8)	
H17A	0.070258	0.445852	0.642561	0.030*	
H17B	−0.088797	0.399768	0.678181	0.030*	
H17C	−0.079237	0.542101	0.723123	0.030*	
N21	0.4354 (5)	0.4647 (4)	0.8123 (3)	0.0203 (7)	
H02	0.382 (7)	0.518 (6)	0.764 (4)	0.030 (15)*	
C22	0.4293 (5)	0.3250 (5)	0.8640 (3)	0.0194 (8)	
C23	0.5459 (6)	0.2340 (5)	0.9360 (3)	0.0234 (9)	
H23	0.544285	0.135196	0.975279	0.028*	
C24	0.6650 (6)	0.2845 (6)	0.9520 (3)	0.0254 (9)	
H24	0.746181	0.219766	1.000858	0.030*	
C25	0.6644 (6)	0.4315 (6)	0.8954 (3)	0.0262 (9)	
H25	0.744037	0.469295	0.905752	0.031*	
C26	0.5479 (6)	0.5197 (5)	0.8252 (3)	0.0233 (9)	
H26	0.545834	0.619640	0.785533	0.028*	
C27	0.3005 (6)	0.2792 (5)	0.8383 (3)	0.0244 (9)	
H27A	0.183763	0.375456	0.840022	0.037*	
H27B	0.299568	0.183031	0.886291	0.037*	
H27C	0.333344	0.248828	0.771654	0.037*	

### Bis(2-picolinium) tetrabromidoaurate(III) bromide (3) Atomic displacement parameters (Å^2^)

**Table:**

	*U*^11^	*U*^22^	*U*^33^	*U*^12^	*U*^13^	*U*^23^
Au1	0.01695 (9)	0.01300 (7)	0.01428 (7)	−0.00855 (6)	−0.00288 (5)	−0.00003 (5)
Br1	0.0220 (2)	0.01968 (18)	0.0236 (2)	−0.01275 (16)	−0.00915 (16)	0.00676 (15)
Br2	0.0201 (2)	0.01958 (18)	0.0278 (2)	−0.01295 (16)	−0.00256 (16)	−0.00265 (15)
Br3	0.0277 (2)	0.02139 (19)	0.0202 (2)	−0.01258 (17)	−0.00617 (17)	0.00623 (15)
Br4	0.0284 (2)	0.0291 (2)	0.0239 (2)	−0.02050 (19)	−0.01034 (17)	0.00396 (16)
Br5	0.0188 (2)	0.02026 (18)	0.0213 (2)	−0.01252 (16)	−0.00712 (15)	0.00465 (14)
N11	0.0130 (18)	0.0147 (14)	0.0201 (17)	−0.0066 (13)	−0.0024 (13)	−0.0015 (12)
C12	0.014 (2)	0.0153 (16)	0.0190 (19)	−0.0073 (15)	0.0024 (15)	−0.0059 (14)
C13	0.014 (2)	0.0223 (19)	0.027 (2)	−0.0104 (17)	0.0012 (16)	−0.0079 (16)
C14	0.015 (2)	0.031 (2)	0.027 (2)	−0.0099 (18)	−0.0026 (17)	−0.0088 (18)
C15	0.019 (2)	0.023 (2)	0.023 (2)	−0.0048 (18)	0.0006 (17)	−0.0031 (16)
C16	0.022 (2)	0.0188 (18)	0.021 (2)	−0.0091 (17)	0.0005 (17)	−0.0012 (15)
C17	0.019 (2)	0.0199 (18)	0.020 (2)	−0.0097 (17)	−0.0009 (16)	−0.0009 (15)
N21	0.021 (2)	0.0204 (16)	0.0190 (18)	−0.0096 (15)	−0.0051 (14)	0.0015 (14)
C22	0.021 (2)	0.0168 (18)	0.0174 (19)	−0.0074 (16)	0.0031 (16)	−0.0046 (15)
C23	0.027 (2)	0.0196 (19)	0.020 (2)	−0.0098 (18)	−0.0018 (17)	−0.0002 (16)
C24	0.027 (3)	0.027 (2)	0.018 (2)	−0.0090 (19)	−0.0064 (17)	−0.0007 (16)
C25	0.026 (3)	0.034 (2)	0.024 (2)	−0.018 (2)	−0.0012 (18)	−0.0060 (18)
C26	0.026 (2)	0.023 (2)	0.025 (2)	−0.0157 (18)	−0.0023 (18)	−0.0005 (16)
C27	0.026 (2)	0.025 (2)	0.027 (2)	−0.0147 (19)	−0.0026 (18)	−0.0039 (17)

### Bis(2-picolinium) tetrabromidoaurate(III) bromide (3) Geometric parameters (Å, º)

**Table:**

Au1—Br1	2.4206 (4)	C17—H17B	0.9800
Au1—Br4	2.4232 (4)	C17—H17C	0.9800
Au1—Br3	2.4243 (4)	N21—C22	1.338 (5)
Au1—Br2	2.4314 (4)	N21—C26	1.345 (5)
N11—C16	1.337 (5)	N21—H02	0.80 (4)
N11—C12	1.348 (5)	C22—C23	1.380 (6)
N11—H01	0.80 (4)	C22—C27	1.485 (6)
C12—C13	1.386 (5)	C23—C24	1.383 (6)
C12—C17	1.484 (6)	C23—H23	0.9500
C13—C14	1.378 (6)	C24—C25	1.395 (6)
C13—H13	0.9500	C24—H24	0.9500
C14—C15	1.385 (6)	C25—C26	1.359 (6)
C14—H14	0.9500	C25—H25	0.9500
C15—C16	1.377 (6)	C26—H26	0.9500
C15—H15	0.9500	C27—H27A	0.9800
C16—H16	0.9500	C27—H27B	0.9800
C17—H17A	0.9800	C27—H27C	0.9800
			
Br1—Au1—Br4	90.687 (14)	C12—C17—H17C	109.5
Br1—Au1—Br3	177.503 (16)	H17A—C17—H17C	109.5
Br4—Au1—Br3	91.130 (15)	H17B—C17—H17C	109.5
Br1—Au1—Br2	89.069 (14)	C22—N21—C26	124.5 (4)
Br4—Au1—Br2	175.465 (16)	C22—N21—H02	123 (4)
Br3—Au1—Br2	89.255 (15)	C26—N21—H02	112 (4)
C16—N11—C12	124.4 (4)	N21—C22—C23	116.8 (4)
C16—N11—H01	120 (4)	N21—C22—C27	117.7 (4)
C12—N11—H01	116 (4)	C23—C22—C27	125.5 (4)
N11—C12—C13	117.0 (4)	C22—C23—C24	121.1 (4)
N11—C12—C17	118.0 (3)	C22—C23—H23	119.5
C13—C12—C17	125.0 (4)	C24—C23—H23	119.5
C14—C13—C12	120.2 (4)	C23—C24—C25	119.2 (4)
C14—C13—H13	119.9	C23—C24—H24	120.4
C12—C13—H13	119.9	C25—C24—H24	120.4
C13—C14—C15	120.7 (4)	C26—C25—C24	118.8 (4)
C13—C14—H14	119.6	C26—C25—H25	120.6
C15—C14—H14	119.6	C24—C25—H25	120.6
C16—C15—C14	118.0 (4)	N21—C26—C25	119.7 (4)
C16—C15—H15	121.0	N21—C26—H26	120.2
C14—C15—H15	121.0	C25—C26—H26	120.2
N11—C16—C15	119.7 (4)	C22—C27—H27A	109.5
N11—C16—H16	120.1	C22—C27—H27B	109.5
C15—C16—H16	120.1	H27A—C27—H27B	109.5
C12—C17—H17A	109.5	C22—C27—H27C	109.5
C12—C17—H17B	109.5	H27A—C27—H27C	109.5
H17A—C17—H17B	109.5	H27B—C27—H27C	109.5
			
C16—N11—C12—C13	0.1 (6)	C26—N21—C22—C23	0.9 (6)
C16—N11—C12—C17	−179.4 (4)	C26—N21—C22—C27	−178.2 (4)
N11—C12—C13—C14	0.1 (6)	N21—C22—C23—C24	−1.2 (6)
C17—C12—C13—C14	179.5 (4)	C27—C22—C23—C24	177.7 (4)
C12—C13—C14—C15	−0.2 (7)	C22—C23—C24—C25	1.2 (7)
C13—C14—C15—C16	0.1 (7)	C23—C24—C25—C26	−0.9 (7)
C12—N11—C16—C15	−0.1 (6)	C22—N21—C26—C25	−0.5 (7)
C14—C15—C16—N11	0.0 (6)	C24—C25—C26—N21	0.5 (7)

### Bis(2-picolinium) tetrabromidoaurate(III) bromide (3) Hydrogen-bond geometry (Å, º)

**Table:**

*D*—H···*A*	*D*—H	H···*A*	*D*···*A*	*D*—H···*A*
N11—H01···Br5	0.80 (4)	2.43 (4)	3.225 (3)	172 (6)
N21—H02···Br5	0.80 (4)	2.39 (4)	3.191 (4)	173 (5)
C13—H13···Br1^i^	0.95	3.13	3.961 (4)	146
C15—H15···Br2^ii^	0.95	3.07	3.941 (4)	152
C16—H16···Br1	0.95	3.08	3.614 (4)	117
C16—H16···Br5^iii^	0.95	2.91	3.751 (4)	148
C17—H17*B*···Br1^i^	0.98	2.99	3.932 (4)	162
C17—H17*C*···Br2^iii^	0.98	3.02	3.758 (4)	133
C26—H26···Br2^iv^	0.95	2.81	3.602 (4)	142
C27—H27*A*···Br3^iii^	0.98	3.00	3.800 (5)	140

Symmetry codes: (i) −*x*, −*y*+1, −*z*+1; (ii) *x*−1, *y*, *z*; (iii) −*x*, −*y*+2, −*z*+1; (iv) −*x*+1, −*y*+2, −*z*+1.

### 3-Picolinium tetrabromidoaurate(III) (4) Crystal data

**Table:**

(C_6_H_8_N)[AuBr_4_]	*F*(000) = 1080
*M**_r_* = 610.74	*D*_x_ = 3.368 Mg m^−^^3^
Monoclinic, *P*2_1_/*c*	Mo *K*α radiation, λ = 0.71073 Å
*a* = 8.1918 (3) Å	Cell parameters from 4240 reflections
*b* = 9.3458 (3) Å	θ = 2.5–29.2°
*c* = 16.1449 (6) Å	µ = 25.43 mm^−^^1^
β = 102.949 (4)°	*T* = 100 K
*V* = 1204.59 (8) Å^3^	Block, red
*Z* = 4	0.08 × 0.03 × 0.02 mm

### 3-Picolinium tetrabromidoaurate(III) (4) Data collection

**Table:**

Oxford Diffraction Xcalibur, Eos diffractometer	2983 independent reflections
Radiation source: Enhance (Mo) X-ray Source	2244 reflections with *I* > 2σ(*I*)
Graphite monochromator	*R*_int_ = 0.079
Detector resolution: 16.1419 pixels mm^-1^	θ_max_ = 28.3°, θ_min_ = 2.5°
ω scan	*h* = −10→10
Absorption correction: multi-scan (CrysAlisPro; Rigaku OD, 2020)	*k* = −12→12
*T*_min_ = 0.328, *T*_max_ = 1.000	*l* = −21→21
38517 measured reflections	

### 3-Picolinium tetrabromidoaurate(III) (4) Refinement

**Table:**

Refinement on *F*^2^	Primary atom site location: structure-invariant direct methods
Least-squares matrix: full	Secondary atom site location: difference Fourier map
*R*[*F*^2^ > 2σ(*F*^2^)] = 0.031	Hydrogen site location: mixed
*wR*(*F*^2^) = 0.052	H atoms treated by a mixture of independent and constrained refinement
*S* = 1.04	*w* = 1/[σ^2^(*F*_o_^2^) + (0.0131*P*)^2^ + 1.6068*P*] where *P* = (*F*_o_^2^ + 2*F*_c_^2^)/3
2983 reflections	(Δ/σ)_max_ < 0.001
117 parameters	Δρ_max_ = 1.05 e Å^−^^3^
0 restraints	Δρ_min_ = −0.98 e Å^−^^3^

### 3-Picolinium tetrabromidoaurate(III) (4) Fractional atomic coordinates and isotropic or equivalent isotropic displacement parameters (Å^2^)

**Table:**

	*x*	*y*	*z*	*U*_iso_*/*U*_eq_	
Au1	0.500000	0.000000	0.500000	0.01472 (8)	
Au2	0.000000	0.000000	0.000000	0.01548 (8)	
Br1	0.37656 (7)	0.00308 (6)	0.34880 (4)	0.01977 (13)	
Br2	0.66401 (7)	0.21010 (6)	0.48320 (4)	0.02255 (14)	
Br3	0.08913 (8)	−0.01593 (6)	0.15315 (4)	0.02355 (14)	
Br4	0.22342 (8)	0.16900 (7)	−0.00263 (4)	0.02747 (16)	
N11	0.3806 (7)	0.3632 (6)	0.3076 (4)	0.0279 (13)	
H01	0.417 (7)	0.278 (7)	0.335 (4)	0.032 (19)*	
C12	0.2612 (8)	0.3555 (7)	0.2368 (4)	0.0271 (15)	
H12	0.224248	0.264649	0.213644	0.033*	
C13	0.1909 (7)	0.4771 (7)	0.1971 (4)	0.0255 (14)	
C14	0.2465 (8)	0.6064 (7)	0.2351 (4)	0.0287 (16)	
H14	0.199190	0.693320	0.210112	0.034*	
C15	0.3716 (9)	0.6102 (7)	0.3098 (5)	0.0346 (17)	
H15	0.410317	0.699323	0.335028	0.041*	
C16	0.4371 (9)	0.4870 (7)	0.3458 (4)	0.0327 (16)	
H16	0.521161	0.487523	0.396967	0.039*	
C17	0.0584 (8)	0.4692 (8)	0.1181 (5)	0.0411 (19)	
H17A	0.110098	0.463148	0.069059	0.062*	
H17B	−0.011840	0.555011	0.113177	0.062*	
H17C	−0.010741	0.384151	0.119848	0.062*	

### 3-Picolinium tetrabromidoaurate(III) (4) Atomic displacement parameters (Å^2^)

**Table:**

	*U*^11^	*U*^22^	*U*^33^	*U*^12^	*U*^13^	*U*^23^
Au1	0.01585 (15)	0.01527 (15)	0.01316 (16)	0.00233 (13)	0.00351 (12)	0.00140 (13)
Au2	0.01751 (16)	0.01467 (15)	0.01457 (16)	0.00047 (13)	0.00424 (12)	0.00174 (13)
Br1	0.0257 (3)	0.0191 (3)	0.0131 (3)	0.0007 (3)	0.0013 (2)	0.0016 (3)
Br2	0.0240 (3)	0.0215 (3)	0.0212 (3)	−0.0038 (3)	0.0030 (3)	0.0038 (3)
Br3	0.0310 (3)	0.0238 (3)	0.0148 (3)	−0.0015 (3)	0.0031 (3)	0.0018 (3)
Br4	0.0283 (3)	0.0277 (3)	0.0267 (4)	−0.0106 (3)	0.0070 (3)	0.0024 (3)
N11	0.040 (3)	0.019 (3)	0.023 (3)	−0.001 (3)	0.004 (3)	0.007 (2)
C12	0.036 (4)	0.022 (3)	0.027 (4)	0.003 (3)	0.014 (3)	0.005 (3)
C13	0.019 (3)	0.033 (4)	0.026 (4)	−0.002 (3)	0.009 (3)	0.003 (3)
C14	0.037 (4)	0.025 (3)	0.029 (4)	0.003 (3)	0.016 (3)	0.005 (3)
C15	0.053 (5)	0.026 (4)	0.033 (4)	−0.005 (3)	0.026 (4)	−0.008 (3)
C16	0.044 (4)	0.033 (4)	0.021 (4)	−0.007 (3)	0.006 (3)	−0.004 (3)
C17	0.027 (4)	0.064 (5)	0.031 (4)	0.002 (4)	0.005 (3)	0.004 (4)

### 3-Picolinium tetrabromidoaurate(III) (4) Geometric parameters (Å, º)

**Table:**

Au1—Br1^i^	2.4241 (6)	C12—H12	0.9500
Au1—Br1	2.4241 (6)	C13—C14	1.385 (8)
Au1—Br2	2.4284 (6)	C13—C17	1.480 (9)
Au1—Br2^i^	2.4285 (6)	C14—C15	1.397 (9)
Au2—Br3^ii^	2.4206 (6)	C14—H14	0.9500
Au2—Br3	2.4207 (6)	C15—C16	1.346 (9)
Au2—Br4	2.4251 (6)	C15—H15	0.9500
Au2—Br4^ii^	2.4251 (6)	C16—H16	0.9500
N11—C12	1.329 (8)	C17—H17A	0.9800
N11—C16	1.343 (8)	C17—H17B	0.9800
N11—H01	0.93 (6)	C17—H17C	0.9800
C12—C13	1.367 (8)		
			
Br1^i^—Au1—Br1	180.0	C12—C13—C14	117.1 (6)
Br1^i^—Au1—Br2	90.32 (2)	C12—C13—C17	120.9 (6)
Br1—Au1—Br2	89.68 (2)	C14—C13—C17	122.0 (6)
Br1^i^—Au1—Br2^i^	89.68 (2)	C13—C14—C15	120.6 (6)
Br1—Au1—Br2^i^	90.33 (2)	C13—C14—H14	119.7
Br2—Au1—Br2^i^	180.0	C15—C14—H14	119.7
Br3^ii^—Au2—Br3	180.0	C16—C15—C14	119.7 (6)
Br3^ii^—Au2—Br4	89.89 (2)	C16—C15—H15	120.2
Br3—Au2—Br4	90.11 (2)	C14—C15—H15	120.2
Br3^ii^—Au2—Br4^ii^	90.11 (2)	N11—C16—C15	118.4 (7)
Br3—Au2—Br4^ii^	89.89 (2)	N11—C16—H16	120.8
Br4—Au2—Br4^ii^	180.0	C15—C16—H16	120.8
C12—N11—C16	123.5 (6)	C13—C17—H17A	109.5
C12—N11—H01	118 (4)	C13—C17—H17B	109.5
C16—N11—H01	118 (4)	H17A—C17—H17B	109.5
N11—C12—C13	120.7 (6)	C13—C17—H17C	109.5
N11—C12—H12	119.7	H17A—C17—H17C	109.5
C13—C12—H12	119.7	H17B—C17—H17C	109.5
			
C16—N11—C12—C13	1.8 (10)	C17—C13—C14—C15	179.9 (6)
N11—C12—C13—C14	−1.7 (9)	C13—C14—C15—C16	−0.9 (10)
N11—C12—C13—C17	179.7 (6)	C12—N11—C16—C15	−1.3 (11)
C12—C13—C14—C15	1.3 (9)	C14—C15—C16—N11	0.8 (11)

Symmetry codes: (i) −*x*+1, −*y*, −*z*+1; (ii) −*x*, −*y*, −*z*.

### 3-Picolinium tetrabromidoaurate(III) (4) Hydrogen-bond geometry (Å, º)

**Table:**

*D*—H···*A*	*D*—H	H···*A*	*D*···*A*	*D*—H···*A*
N11—H01···Br1	0.93 (6)	2.61 (6)	3.432 (5)	149 (5)
N11—H01···Br2	0.93 (6)	2.84 (6)	3.539 (6)	134 (5)
C12—H12···Br3	0.95	2.93	3.874 (7)	175
C15—H15···Br1^iii^	0.95	2.87	3.724 (7)	151
C16—H16···Br2	0.95	3.05	3.637 (7)	122
C16—H16···Br4^iv^	0.95	2.93	3.726 (7)	143

Symmetry codes: (iii) *x*, *y*+1, *z*; (iv) −*x*+1, *y*+1/2, −*z*+1/2.

### trans-Dibromidobis(4-picoline)gold(III) tetrabromidoaurate(III) nitromethane monosolvate (5) Crystal data

**Table:**

[AuBr_2_(C_6_H_7_N)_2_](AuBr_4_]·CH_3_NO_2_	*Z* = 2
*M**_r_* = 1120.69	*F*(000) = 1000
Triclinic, *P*1	*D*_x_ = 3.124 Mg m^−^^3^
*a* = 7.5336 (4) Å	Mo *K*α radiation, λ = 0.71073 Å
*b* = 12.49946 (10) Å	Cell parameters from 9644 reflections
*c* = 12.74241 (10) Å	θ = 3.1–27.8°
α = 84.400 (6)°	µ = 22.38 mm^−^^1^
β = 89.908 (5)°	*T* = 101 K
γ = 86.012 (5)°	Plate, red
*V* = 1191.26 (7) Å^3^	0.20 × 0.08 × 0.01 mm

### trans-Dibromidobis(4-picoline)gold(III) tetrabromidoaurate(III) nitromethane monosolvate (5) Data collection

**Table:**

Oxford Diffraction Xcalibur, Eos diffractometer	6279 independent reflections
Radiation source: Enhance (Mo) X-ray Source	5020 reflections with *I* > 2σ(*I*)
Graphite monochromator	*R*_int_ = 0.104
Detector resolution: 16.1419 pixels mm^-1^	θ_max_ = 28.3°, θ_min_ = 2.2°
ω scans	*h* = −10→10
Absorption correction: multi-scan (CrysAlisPro; Rigaku OD, 2020)	*k* = −16→16
*T*_min_ = 0.140, *T*_max_ = 1.000	*l* = −16→16
6279 measured reflections	

### trans-Dibromidobis(4-picoline)gold(III) tetrabromidoaurate(III) nitromethane monosolvate (5) Refinement

**Table:**

Refinement on *F*^2^	Primary atom site location: structure-invariant direct methods
Least-squares matrix: full	Secondary atom site location: difference Fourier map
*R*[*F*^2^ > 2σ(*F*^2^)] = 0.038	Hydrogen site location: inferred from neighbouring sites
*wR*(*F*^2^) = 0.063	H-atom parameters constrained
*S* = 0.94	*w* = 1/[σ^2^(*F*_o_^2^) + (0.0252*P*)^2^] where *P* = (*F*_o_^2^ + 2*F*_c_^2^)/3
6279 reflections	(Δ/σ)_max_ = 0.003
222 parameters	Δρ_max_ = 2.43 e Å^−^^3^
84 restraints	Δρ_min_ = −1.74 e Å^−^^3^

### trans-Dibromidobis(4-picoline)gold(III) tetrabromidoaurate(III) nitromethane monosolvate (5) Fractional atomic coordinates and isotropic or equivalent isotropic displacement parameters (Å^2^)

**Table:**

	*x*	*y*	*z*	*U*_iso_*/*U*_eq_	
Au1	0.000000	0.500000	0.000000	0.00944 (13)	
Au2	0.500000	0.500000	0.500000	0.01041 (13)	
Au3	0.77534 (5)	0.79410 (4)	0.20867 (3)	0.01215 (10)	
Br1	0.24353 (13)	0.59056 (9)	0.06730 (8)	0.0154 (2)	
Br2	0.22259 (13)	0.58165 (9)	0.42403 (8)	0.0158 (2)	
Br3	0.84829 (15)	0.93250 (9)	0.31755 (8)	0.0233 (3)	
Br4	0.79712 (17)	0.91994 (10)	0.05310 (8)	0.0263 (3)	
Br5	0.68233 (13)	0.65824 (9)	0.10090 (8)	0.0148 (2)	
Br6	0.77248 (14)	0.66483 (9)	0.36344 (8)	0.0169 (3)	
N11	0.0090 (10)	0.3960 (7)	0.1331 (6)	0.0067 (18)	
C12	0.0297 (12)	0.2882 (9)	0.1240 (8)	0.013 (2)	
H12	0.036614	0.262700	0.056126	0.016*	
C13	0.0406 (13)	0.2166 (10)	0.2124 (8)	0.019 (2)	
H13	0.062460	0.142055	0.204576	0.023*	
C14	0.0208 (13)	0.2492 (9)	0.3144 (8)	0.015 (2)	
C15	−0.0010 (13)	0.3615 (9)	0.3170 (8)	0.018 (2)	
H15	−0.011695	0.390088	0.383267	0.021*	
C16	−0.0074 (12)	0.4314 (8)	0.2261 (7)	0.011 (2)	
H16	−0.023901	0.506762	0.231234	0.014*	
C17	0.0267 (14)	0.1743 (9)	0.4093 (8)	0.023 (3)	
H17A	−0.055374	0.118067	0.401622	0.034*	
H17B	−0.008381	0.213182	0.470056	0.034*	
H17C	0.147905	0.141219	0.420182	0.034*	
N21	0.4855 (10)	0.6030 (7)	0.6127 (6)	0.0060 (18)	
C22	0.4823 (13)	0.5669 (9)	0.7143 (8)	0.017 (3)	
H22	0.491126	0.491270	0.733175	0.021*	
C23	0.4668 (12)	0.6346 (9)	0.7931 (8)	0.011 (2)	
H23	0.462877	0.605894	0.864793	0.013*	
C24	0.4568 (13)	0.7448 (9)	0.7672 (8)	0.013 (2)	
C25	0.4612 (13)	0.7810 (10)	0.6609 (8)	0.020 (3)	
H25	0.455491	0.856246	0.640001	0.024*	
C26	0.4739 (13)	0.7089 (9)	0.5848 (8)	0.013 (2)	
H26	0.474284	0.735097	0.512306	0.016*	
C27	0.4375 (14)	0.8215 (9)	0.8526 (8)	0.019 (3)	
H27A	0.555048	0.831116	0.881651	0.029*	
H27B	0.361279	0.791532	0.908827	0.029*	
H27C	0.383740	0.891352	0.822309	0.029*	
N1	0.3239 (14)	0.9121 (9)	0.2892 (8)	0.036 (3)*	
O1	0.3561 (15)	0.8660 (10)	0.3761 (9)	0.083 (4)*	
O2	0.3164 (11)	1.0091 (7)	0.2727 (6)	0.044 (3)*	
C1	0.290 (2)	0.8516 (14)	0.2045 (14)	0.079 (5)*	
H1A	0.357624	0.877843	0.142668	0.119*	
H1B	0.162431	0.859438	0.187551	0.119*	
H1C	0.325698	0.775437	0.224327	0.119*	

### trans-Dibromidobis(4-picoline)gold(III) tetrabromidoaurate(III) nitromethane monosolvate (5) Atomic displacement parameters (Å^2^)

**Table:**

	*U*^11^	*U*^22^	*U*^33^	*U*^12^	*U*^13^	*U*^23^
Au1	0.0104 (3)	0.0106 (3)	0.0074 (3)	−0.0013 (3)	−0.0005 (2)	−0.0011 (3)
Au2	0.0109 (3)	0.0126 (3)	0.0083 (3)	−0.0021 (3)	0.0008 (2)	−0.0025 (3)
Au3	0.0126 (2)	0.0129 (2)	0.01104 (18)	−0.0007 (2)	0.00025 (16)	−0.0020 (2)
Br1	0.0152 (6)	0.0178 (6)	0.0137 (5)	−0.0058 (5)	−0.0038 (4)	−0.0013 (5)
Br2	0.0141 (6)	0.0204 (6)	0.0133 (5)	0.0010 (5)	−0.0022 (4)	−0.0044 (5)
Br3	0.0328 (8)	0.0207 (7)	0.0176 (6)	−0.0056 (5)	−0.0034 (5)	−0.0051 (5)
Br4	0.0480 (8)	0.0161 (7)	0.0146 (6)	−0.0023 (6)	0.0027 (5)	0.0005 (5)
Br5	0.0154 (6)	0.0161 (6)	0.0137 (5)	−0.0028 (5)	−0.0004 (4)	−0.0041 (5)
Br6	0.0208 (6)	0.0182 (7)	0.0117 (5)	−0.0043 (5)	0.0012 (5)	0.0002 (5)
N11	0.006 (2)	0.007 (3)	0.007 (2)	−0.0008 (18)	−0.0004 (18)	−0.0006 (18)
C12	0.014 (3)	0.013 (3)	0.012 (3)	−0.0006 (19)	0.0008 (19)	−0.0019 (19)
C13	0.020 (3)	0.018 (3)	0.019 (3)	−0.002 (2)	0.0011 (19)	−0.0022 (19)
C14	0.015 (3)	0.014 (3)	0.015 (3)	−0.0023 (19)	−0.0012 (19)	−0.0001 (19)
C15	0.019 (3)	0.019 (3)	0.016 (3)	−0.002 (2)	−0.0006 (19)	−0.0028 (19)
C16	0.012 (3)	0.011 (3)	0.011 (3)	−0.0009 (19)	0.0007 (19)	−0.0011 (19)
C17	0.027 (4)	0.024 (4)	0.017 (4)	0.002 (3)	−0.001 (3)	0.000 (3)
N21	0.006 (2)	0.006 (2)	0.007 (2)	−0.0012 (18)	0.0012 (18)	0.0009 (18)
C22	0.018 (3)	0.017 (3)	0.017 (3)	−0.0019 (19)	−0.0003 (19)	−0.0018 (19)
C23	0.011 (3)	0.012 (3)	0.010 (3)	0.0012 (19)	0.0010 (19)	−0.0009 (19)
C24	0.012 (3)	0.014 (3)	0.014 (3)	0.0000 (19)	−0.0011 (19)	−0.0029 (19)
C25	0.019 (3)	0.020 (3)	0.020 (3)	−0.001 (2)	−0.0007 (19)	−0.002 (2)
C26	0.014 (3)	0.014 (3)	0.012 (3)	−0.0005 (19)	0.0014 (19)	−0.0002 (19)
C27	0.021 (4)	0.016 (4)	0.020 (4)	−0.002 (3)	0.000 (3)	0.001 (3)

### trans-Dibromidobis(4-picoline)gold(III) tetrabromidoaurate(III) nitromethane monosolvate (5) Geometric parameters (Å, º)

**Table:**

Au1—N11^i^	2.032 (8)	C17—H17A	0.9800
Au1—N11	2.032 (8)	C17—H17B	0.9800
Au1—Br1	2.4220 (10)	C17—H17C	0.9800
Au1—Br1^i^	2.4220 (10)	N21—C22	1.329 (12)
Au2—N21^ii^	2.019 (8)	N21—C26	1.333 (13)
Au2—N21	2.019 (8)	C22—C23	1.375 (13)
Au2—Br2	2.4214 (11)	C22—H22	0.9500
Au2—Br2^ii^	2.4214 (11)	C23—C24	1.382 (14)
Au3—Br3	2.4130 (12)	C23—H23	0.9500
Au3—Br4	2.4201 (12)	C24—C25	1.386 (14)
Au3—Br6	2.4257 (12)	C24—C27	1.519 (13)
Au3—Br5	2.4258 (12)	C25—C26	1.386 (14)
N11—C16	1.308 (11)	C25—H25	0.9500
N11—C12	1.361 (13)	C26—H26	0.9500
C12—C13	1.368 (14)	C27—H27A	0.9800
C12—H12	0.9500	C27—H27B	0.9800
C13—C14	1.403 (14)	C27—H27C	0.9800
C13—H13	0.9500	N1—O2	1.208 (12)
C14—C15	1.406 (14)	N1—O1	1.213 (14)
C14—C17	1.454 (13)	N1—C1	1.411 (17)
C15—C16	1.380 (13)	C1—H1A	0.9800
C15—H15	0.9500	C1—H1B	0.9800
C16—H16	0.9500	C1—H1C	0.9800
			
N11^i^—Au1—N11	180.0	H17A—C17—H17B	109.5
N11^i^—Au1—Br1	90.3 (2)	C14—C17—H17C	109.5
N11—Au1—Br1	89.7 (2)	H17A—C17—H17C	109.5
N11^i^—Au1—Br1^i^	89.7 (2)	H17B—C17—H17C	109.5
N11—Au1—Br1^i^	90.3 (2)	C22—N21—C26	119.5 (9)
Br1—Au1—Br1^i^	180.0	C22—N21—Au2	120.9 (7)
N21^ii^—Au2—N21	180.0 (4)	C26—N21—Au2	119.5 (7)
N21^ii^—Au2—Br2	89.7 (2)	N21—C22—C23	122.6 (10)
N21—Au2—Br2	90.3 (2)	N21—C22—H22	118.7
N21^ii^—Au2—Br2^ii^	90.3 (2)	C23—C22—H22	118.7
N21—Au2—Br2^ii^	89.7 (2)	C22—C23—C24	119.5 (10)
Br2—Au2—Br2^ii^	180.0	C22—C23—H23	120.2
Br3—Au3—Br4	89.83 (4)	C24—C23—H23	120.2
Br3—Au3—Br6	90.19 (4)	C23—C24—C25	117.1 (10)
Br4—Au3—Br6	176.54 (5)	C23—C24—C27	120.6 (9)
Br3—Au3—Br5	176.38 (4)	C25—C24—C27	122.3 (10)
Br4—Au3—Br5	90.39 (4)	C26—C25—C24	120.9 (11)
Br6—Au3—Br5	89.81 (4)	C26—C25—H25	119.6
C16—N11—C12	120.3 (9)	C24—C25—H25	119.6
C16—N11—Au1	120.8 (7)	N21—C26—C25	120.5 (10)
C12—N11—Au1	118.9 (6)	N21—C26—H26	119.8
N11—C12—C13	120.0 (10)	C25—C26—H26	119.8
N11—C12—H12	120.0	C24—C27—H27A	109.5
C13—C12—H12	120.0	C24—C27—H27B	109.5
C12—C13—C14	122.5 (11)	H27A—C27—H27B	109.5
C12—C13—H13	118.8	C24—C27—H27C	109.5
C14—C13—H13	118.8	H27A—C27—H27C	109.5
C13—C14—C15	113.9 (10)	H27B—C27—H27C	109.5
C13—C14—C17	123.4 (10)	O2—N1—O1	122.1 (12)
C15—C14—C17	122.7 (10)	O2—N1—C1	118.2 (13)
C16—C15—C14	122.0 (10)	O1—N1—C1	119.6 (13)
C16—C15—H15	119.0	N1—C1—H1A	109.5
C14—C15—H15	119.0	N1—C1—H1B	109.5
N11—C16—C15	121.3 (10)	H1A—C1—H1B	109.5
N11—C16—H16	119.4	N1—C1—H1C	109.5
C15—C16—H16	119.4	H1A—C1—H1C	109.5
C14—C17—H17A	109.5	H1B—C1—H1C	109.5
C14—C17—H17B	109.5		
			
C16—N11—C12—C13	−2.6 (15)	C26—N21—C22—C23	0.2 (15)
Au1—N11—C12—C13	178.4 (7)	Au2—N21—C22—C23	−177.9 (7)
N11—C12—C13—C14	3.8 (16)	N21—C22—C23—C24	−1.1 (15)
C12—C13—C14—C15	−3.4 (15)	C22—C23—C24—C25	0.7 (15)
C12—C13—C14—C17	178.1 (9)	C22—C23—C24—C27	179.3 (9)
C13—C14—C15—C16	1.9 (15)	C23—C24—C25—C26	0.4 (15)
C17—C14—C15—C16	−179.6 (9)	C27—C24—C25—C26	−178.1 (9)
C12—N11—C16—C15	1.1 (15)	C22—N21—C26—C25	1.0 (14)
Au1—N11—C16—C15	−179.8 (7)	Au2—N21—C26—C25	179.1 (7)
C14—C15—C16—N11	−0.8 (16)	C24—C25—C26—N21	−1.3 (15)

Symmetry codes: (i) −*x*, −*y*+1, −*z*; (ii) −*x*+1, −*y*+1, −*z*+1.

### trans-Dibromidobis(4-picoline)gold(III) tetrabromidoaurate(III) nitromethane monosolvate (5) Hydrogen-bond geometry (Å, º)

**Table:**

*D*—H···*A*	*D*—H	H···*A*	*D*···*A*	*D*—H···*A*
C12—H12···Br4^iii^	0.95	2.99	3.771 (11)	141
C12—H12···Br5^iii^	0.95	3.06	3.629 (10)	120
C13—H13···O2^iv^	0.95	2.54	3.240 (15)	131
C15—H15···Br2^v^	0.95	2.96	3.802 (10)	149
C16—H16···Br6^vi^	0.95	3.05	3.826 (10)	140
C22—H22···Br5^ii^	0.95	3.04	3.766 (11)	135
C23—H23···Br1^vii^	0.95	3.06	3.883 (9)	146
C26—H26···Br6	0.95	3.07	3.665 (10)	122
C26—H26···O1	0.95	2.39	3.235 (16)	147
C1—H1*B*···Br3^vi^	0.98	3.03	3.737 (17)	130

Symmetry codes: (ii) −*x*+1, −*y*+1, −*z*+1; (iii) −*x*+1, −*y*+1, −*z*; (iv) *x*, *y*−1, *z*; (v) −*x*, −*y*+1, −*z*+1; (vi) *x*−1, *y*, *z*; (vii) *x*, *y*, *z*+1.

### 4-Picolinium tetrabromidoaurate(III) (6) Crystal data

**Table:**

(C_6_H_8_N)[AuBr_4_]	*Z* = 2
*M**_r_* = 610.74	*F*(000) = 540
Triclinic, *P*1	*D*_x_ = 3.393 Mg m^−^^3^
*a* = 7.5701 (3) Å	Mo *K*α radiation, λ = 0.71073 Å
*b* = 9.5159 (5) Å	Cell parameters from 9482 reflections
*c* = 9.5653 (5) Å	θ = 2.4–30.5°
α = 112.616 (5)°	µ = 25.63 mm^−^^1^
β = 104.788 (4)°	*T* = 100 K
γ = 96.401 (4)°	Plate, red
*V* = 597.79 (6) Å^3^	0.18 × 0.10 × 0.01 mm

### 4-Picolinium tetrabromidoaurate(III) (6) Data collection

**Table:**

Oxford Diffraction Xcalibur, Eos diffractometer	3555 independent reflections
Radiation source: Enhance (Mo) X-ray Source	3064 reflections with *I* > 2σ(*I*)
Graphite monochromator	*R*_int_ = 0.075
Detector resolution: 16.1419 pixels mm^-1^	θ_max_ = 30.9°, θ_min_ = 2.4°
ω scan	*h* = −10→10
Absorption correction: multi-scan (CrysAlisPro; Rigaku OD, 2020)	*k* = −13→13
*T*_min_ = 0.298, *T*_max_ = 1.000	*l* = −13→13
41754 measured reflections	

### 4-Picolinium tetrabromidoaurate(III) (6) Refinement

**Table:**

Refinement on *F*^2^	Primary atom site location: structure-invariant direct methods
Least-squares matrix: full	Secondary atom site location: difference Fourier map
*R*[*F*^2^ > 2σ(*F*^2^)] = 0.034	Hydrogen site location: mixed
*wR*(*F*^2^) = 0.092	H atoms treated by a mixture of independent and constrained refinement
*S* = 1.08	*w* = 1/[σ^2^(*F*_o_^2^) + (0.049*P*)^2^ + 1.9196*P*] where *P* = (*F*_o_^2^ + 2*F*_c_^2^)/3
3555 reflections	(Δ/σ)_max_ < 0.001
117 parameters	Δρ_max_ = 2.05 e Å^−^^3^
0 restraints	Δρ_min_ = −1.97 e Å^−^^3^

### 4-Picolinium tetrabromidoaurate(III) (6) Fractional atomic coordinates and isotropic or equivalent isotropic displacement parameters (Å^2^)

**Table:**

	*x*	*y*	*z*	*U*_iso_*/*U*_eq_	
Au1	0.500000	1.000000	1.000000	0.01094 (9)	
Au2	1.000000	0.500000	1.000000	0.01170 (9)	
Br1	0.42459 (9)	0.88962 (8)	0.71154 (7)	0.01804 (14)	
Br2	0.31714 (8)	0.75842 (7)	0.97178 (7)	0.01559 (14)	
Br3	0.75122 (9)	0.63921 (7)	0.98237 (7)	0.01806 (14)	
Br4	0.88137 (9)	0.34545 (7)	0.71095 (7)	0.01707 (14)	
N11	0.3623 (10)	0.4027 (9)	0.6697 (8)	0.0318 (15)	
H01	0.436 (15)	0.476 (13)	0.768 (12)	0.05 (3)*	
C12	0.2662 (12)	0.4402 (9)	0.5560 (9)	0.0315 (17)	
H12	0.272508	0.547031	0.577305	0.038*	
C13	0.1603 (11)	0.3261 (8)	0.4110 (8)	0.0224 (14)	
H13	0.092328	0.352454	0.329997	0.027*	
C14	0.1511 (9)	0.1682 (7)	0.3806 (7)	0.0161 (12)	
C15	0.2515 (10)	0.1375 (9)	0.5043 (8)	0.0222 (14)	
H15	0.245711	0.031775	0.487711	0.027*	
C16	0.3574 (10)	0.2537 (9)	0.6478 (8)	0.0255 (15)	
H16	0.426737	0.230842	0.731097	0.031*	
C17	0.0417 (11)	0.0388 (8)	0.2202 (8)	0.0252 (15)	
H17A	0.125374	−0.024973	0.177908	0.038*	
H17B	−0.013054	0.083136	0.146867	0.038*	
H17C	−0.059019	−0.026879	0.230461	0.038*	

### 4-Picolinium tetrabromidoaurate(III) (6) Atomic displacement parameters (Å^2^)

**Table:**

	*U*^11^	*U*^22^	*U*^33^	*U*^12^	*U*^13^	*U*^23^
Au1	0.01181 (15)	0.01142 (16)	0.01137 (15)	0.00391 (11)	0.00464 (11)	0.00585 (12)
Au2	0.01234 (16)	0.01162 (16)	0.01106 (15)	0.00256 (11)	0.00378 (12)	0.00487 (12)
Br1	0.0241 (3)	0.0179 (3)	0.0120 (3)	0.0044 (2)	0.0066 (2)	0.0059 (2)
Br2	0.0171 (3)	0.0129 (3)	0.0181 (3)	0.0021 (2)	0.0065 (2)	0.0080 (2)
Br3	0.0158 (3)	0.0169 (3)	0.0174 (3)	0.0062 (2)	0.0026 (2)	0.0042 (2)
Br4	0.0209 (3)	0.0174 (3)	0.0112 (3)	0.0053 (2)	0.0038 (2)	0.0051 (2)
N11	0.028 (3)	0.035 (4)	0.020 (3)	−0.002 (3)	0.003 (3)	0.005 (3)
C12	0.043 (5)	0.024 (4)	0.027 (4)	0.003 (3)	0.016 (3)	0.010 (3)
C13	0.034 (4)	0.017 (3)	0.020 (3)	0.011 (3)	0.011 (3)	0.010 (3)
C14	0.017 (3)	0.016 (3)	0.015 (3)	0.007 (2)	0.007 (2)	0.004 (2)
C15	0.023 (3)	0.024 (4)	0.023 (3)	0.012 (3)	0.010 (3)	0.011 (3)
C16	0.024 (4)	0.037 (4)	0.014 (3)	0.008 (3)	0.005 (3)	0.011 (3)
C17	0.027 (4)	0.017 (3)	0.015 (3)	0.004 (3)	−0.003 (3)	−0.004 (3)

### 4-Picolinium tetrabromidoaurate(III) (6) Geometric parameters (Å, º)

**Table:**

Au1—Br1^i^	2.4240 (6)	C12—H12	0.9500
Au1—Br1	2.4240 (6)	C13—C14	1.406 (9)
Au1—Br2	2.4282 (6)	C13—H13	0.9500
Au1—Br2^i^	2.4282 (6)	C14—C15	1.386 (9)
Au2—Br3^ii^	2.4282 (6)	C14—C17	1.493 (9)
Au2—Br3	2.4282 (6)	C15—C16	1.350 (10)
Au2—Br4	2.4340 (6)	C15—H15	0.9500
Au2—Br4^ii^	2.4340 (6)	C16—H16	0.9500
N11—C12	1.339 (10)	C17—H17A	0.9800
N11—C16	1.346 (11)	C17—H17B	0.9800
N11—H01	0.91 (11)	C17—H17C	0.9800
C12—C13	1.351 (10)		
			
Br1^i^—Au1—Br1	180.0	C12—C13—C14	119.7 (6)
Br1^i^—Au1—Br2	90.77 (2)	C12—C13—H13	120.2
Br1—Au1—Br2	89.23 (2)	C14—C13—H13	120.2
Br1^i^—Au1—Br2^i^	89.23 (2)	C15—C14—C13	117.4 (6)
Br1—Au1—Br2^i^	90.77 (2)	C15—C14—C17	121.2 (6)
Br2—Au1—Br2^i^	180.0	C13—C14—C17	121.4 (6)
Br3^ii^—Au2—Br3	180.0 (3)	C16—C15—C14	121.8 (7)
Br3^ii^—Au2—Br4	89.79 (2)	C16—C15—H15	119.1
Br3—Au2—Br4	90.21 (2)	C14—C15—H15	119.1
Br3^ii^—Au2—Br4^ii^	90.21 (2)	N11—C16—C15	118.3 (6)
Br3—Au2—Br4^ii^	89.79 (2)	N11—C16—H16	120.9
Br4—Au2—Br4^ii^	180.0	C15—C16—H16	120.9
C12—N11—C16	122.9 (7)	C14—C17—H17A	109.5
C12—N11—H01	123 (7)	C14—C17—H17B	109.5
C16—N11—H01	114 (7)	H17A—C17—H17B	109.5
N11—C12—C13	120.0 (7)	C14—C17—H17C	109.5
N11—C12—H12	120.0	H17A—C17—H17C	109.5
C13—C12—H12	120.0	H17B—C17—H17C	109.5
			
C16—N11—C12—C13	−0.8 (12)	C13—C14—C15—C16	−1.3 (10)
N11—C12—C13—C14	0.2 (12)	C17—C14—C15—C16	177.1 (7)
C12—C13—C14—C15	0.8 (11)	C12—N11—C16—C15	0.3 (12)
C12—C13—C14—C17	−177.6 (7)	C14—C15—C16—N11	0.7 (11)

Symmetry codes: (i) −*x*+1, −*y*+2, −*z*+2; (ii) −*x*+2, −*y*+1, −*z*+2.

### 4-Picolinium tetrabromidoaurate(III) (6) Hydrogen-bond geometry (Å, º)

**Table:**

*D*—H···*A*	*D*—H	H···*A*	*D*···*A*	*D*—H···*A*
N11—H01···Br2	0.91 (11)	3.04 (10)	3.628 (7)	124 (8)
N11—H01···Br3	0.91 (11)	2.56 (11)	3.395 (7)	154 (9)
C12—H12···Br1	0.95	2.96	3.872 (8)	160
C15—H15···Br1^iii^	0.95	3.07	3.764 (7)	132
C16—H16···Br2^iv^	0.95	2.96	3.890 (7)	166
C17—H17*A*···Br2^v^	0.98	3.04	4.001 (7)	167
C17—H17*C*···Br3^vi^	0.98	3.04	3.660 (7)	123
C13—H13···Br4^vii^	0.95	3.04	3.750 (6)	133

Symmetry codes: (iii) *x*, *y*−1, *z*; (iv) −*x*+1, −*y*+1, −*z*+2; (v) *x*, *y*−1, *z*−1; (vi) *x*−1, *y*−1, *z*−1; (vii) −*x*+1, −*y*+1, −*z*+1.

### 2,4-Dimethylpyridinium tetrabromidoaurate(III) (7) Crystal data

**Table:**

(C_7_H_10_N)[AuBr_4_]	*D*_x_ = 3.201 Mg m^−^^3^
*M**_r_* = 624.77	Mo *K*α radiation, λ = 0.71073 Å
Orthorhombic, *P*2_1_2_1_2_1_	Cell parameters from 8709 reflections
*a* = 8.8797 (3) Å	θ = 2.5–30.2°
*b* = 9.4081 (4) Å	µ = 23.63 mm^−^^1^
*c* = 15.5202 (5) Å	*T* = 100 K
*V* = 1296.57 (8) Å^3^	Block, red
*Z* = 4	0.25 × 0.25 × 0.07 mm
*F*(000) = 1112	

### 2,4-Dimethylpyridinium tetrabromidoaurate(III) (7) Data collection

**Table:**

Oxford Diffraction Xcalibur, Eos diffractometer	3759 independent reflections
Radiation source: fine-focus sealed X-ray tube	3574 reflections with *I* > 2σ(*I*)
Graphite monochromator	*R*_int_ = 0.066
Detector resolution: 16.1419 pixels mm^-1^	θ_max_ = 30.0°, θ_min_ = 2.5°
ω scan	*h* = −12→12
Absorption correction: multi-scan (CrysAlisPro; Rigaku OD, 2020)	*k* = −13→13
*T*_min_ = 0.212, *T*_max_ = 1.000	*l* = −21→21
33591 measured reflections	

### 2,4-Dimethylpyridinium tetrabromidoaurate(III) (7) Refinement

**Table:**

Refinement on *F*^2^	Hydrogen site location: mixed
Least-squares matrix: full	H atoms treated by a mixture of independent and constrained refinement
*R*[*F*^2^ > 2σ(*F*^2^)] = 0.024	*w* = 1/[σ^2^(*F*_o_^2^) + (0.0112*P*)^2^] where *P* = (*F*_o_^2^ + 2*F*_c_^2^)/3
*wR*(*F*^2^) = 0.040	(Δ/σ)_max_ = 0.001
*S* = 1.04	Δρ_max_ = 1.67 e Å^−^^3^
3759 reflections	Δρ_min_ = −1.19 e Å^−^^3^
125 parameters	Extinction correction: SHELXL-2019/3 (Sheldrick, 2015), Fc^*^=kFc[1+0.001xFc^2^λ^3^/sin(2θ)]^-1/4^
0 restraints	Extinction coefficient: 0.00101 (7)
Primary atom site location: structure-invariant direct methods	Absolute structure: Flack *x* determined using 1420 quotients [(*I*^+^)-(*I*^-^)]/[(*I*^+^)+(*I*^-^)] (Parsons *et al.*, 2013)
Secondary atom site location: difference Fourier map	Absolute structure parameter: −0.024 (6)

### 2,4-Dimethylpyridinium tetrabromidoaurate(III) (7) Fractional atomic coordinates and isotropic or equivalent isotropic displacement parameters (Å^2^)

**Table:**

	*x*	*y*	*z*	*U*_iso_*/*U*_eq_	
Au1	0.69472 (2)	0.93066 (3)	0.42645 (2)	0.01312 (6)	
Br1	0.48138 (6)	1.07804 (8)	0.38578 (4)	0.01891 (14)	
Br2	0.71742 (7)	1.05811 (8)	0.56089 (4)	0.02206 (15)	
Br3	0.90010 (7)	0.77649 (8)	0.46964 (4)	0.02138 (16)	
Br4	0.68403 (8)	0.81480 (9)	0.28724 (4)	0.02655 (17)	
N11	0.3103 (8)	0.8502 (7)	0.2341 (4)	0.0275 (14)	
H01	0.388 (8)	0.895 (9)	0.244 (5)	0.04 (3)*	
C12	0.2647 (7)	0.8642 (7)	0.1512 (4)	0.0182 (14)	
C13	0.1412 (7)	0.7843 (8)	0.1267 (4)	0.0194 (15)	
H13	0.104440	0.792167	0.069400	0.023*	
C14	0.0695 (7)	0.6926 (7)	0.1841 (4)	0.0205 (15)	
C15	0.1230 (8)	0.6858 (9)	0.2668 (4)	0.0292 (18)	
H15	0.074949	0.625679	0.307561	0.035*	
C16	0.2448 (8)	0.7647 (9)	0.2911 (4)	0.033 (2)	
H16	0.282451	0.758529	0.348226	0.040*	
C17	0.3522 (8)	0.9567 (9)	0.0929 (5)	0.042 (2)	
H17A	0.391704	1.037926	0.125284	0.063*	
H17B	0.286751	0.990954	0.046443	0.063*	
H17C	0.436017	0.902622	0.068167	0.063*	
C18	−0.0593 (8)	0.6032 (8)	0.1557 (5)	0.0339 (19)	
H18A	−0.021124	0.516777	0.128041	0.051*	
H18B	−0.121009	0.656604	0.114552	0.051*	
H18C	−0.120645	0.577321	0.205779	0.051*	

### 2,4-Dimethylpyridinium tetrabromidoaurate(III) (7) Atomic displacement parameters (Å^2^)

**Table:**

	*U*^11^	*U*^22^	*U*^33^	*U*^12^	*U*^13^	*U*^23^
Au1	0.01223 (11)	0.01297 (12)	0.01416 (10)	−0.00105 (10)	−0.00021 (9)	0.00202 (10)
Br1	0.0168 (3)	0.0151 (3)	0.0248 (3)	0.0019 (3)	−0.0044 (2)	0.0016 (3)
Br2	0.0241 (3)	0.0234 (4)	0.0187 (3)	0.0010 (3)	−0.0030 (2)	−0.0035 (3)
Br3	0.0185 (3)	0.0219 (4)	0.0238 (3)	0.0050 (3)	−0.0030 (3)	0.0027 (3)
Br4	0.0249 (4)	0.0356 (4)	0.0192 (3)	0.0077 (4)	−0.0033 (3)	−0.0082 (3)
N11	0.023 (3)	0.027 (4)	0.033 (3)	0.003 (3)	−0.007 (3)	−0.012 (3)
C12	0.015 (3)	0.013 (3)	0.027 (3)	0.006 (3)	0.004 (3)	0.001 (3)
C13	0.016 (3)	0.026 (4)	0.016 (3)	0.006 (3)	−0.003 (2)	−0.004 (3)
C14	0.016 (3)	0.014 (4)	0.032 (4)	0.006 (3)	0.003 (3)	−0.005 (3)
C15	0.033 (4)	0.031 (5)	0.023 (4)	0.010 (4)	0.010 (3)	0.007 (3)
C16	0.041 (4)	0.044 (6)	0.015 (3)	0.014 (4)	−0.005 (3)	−0.001 (3)
C17	0.035 (4)	0.023 (5)	0.067 (6)	0.003 (4)	0.015 (4)	0.010 (4)
C18	0.021 (4)	0.021 (4)	0.060 (5)	−0.001 (3)	0.009 (4)	−0.014 (4)

### 2,4-Dimethylpyridinium tetrabromidoaurate(III) (7) Geometric parameters (Å, º)

**Table:**

Au1—Br2	2.4151 (7)	C14—C15	1.371 (9)
Au1—Br4	2.4217 (7)	C14—C18	1.486 (9)
Au1—Br3	2.4247 (7)	C15—C16	1.365 (10)
Au1—Br1	2.4310 (7)	C15—H15	0.9500
N11—C16	1.330 (10)	C16—H16	0.9500
N11—C12	1.355 (8)	C17—H17A	0.9800
N11—H01	0.82 (8)	C17—H17B	0.9800
C12—C13	1.383 (9)	C17—H17C	0.9800
C12—C17	1.477 (9)	C18—H18A	0.9800
C13—C14	1.395 (9)	C18—H18B	0.9800
C13—H13	0.9500	C18—H18C	0.9800
			
Br2—Au1—Br4	175.99 (3)	C16—C15—C14	120.5 (7)
Br2—Au1—Br3	89.73 (2)	C16—C15—H15	119.7
Br4—Au1—Br3	90.40 (3)	C14—C15—H15	119.7
Br2—Au1—Br1	90.35 (2)	N11—C16—C15	119.5 (7)
Br4—Au1—Br1	89.69 (2)	N11—C16—H16	120.2
Br3—Au1—Br1	177.57 (3)	C15—C16—H16	120.2
C16—N11—C12	124.0 (7)	C12—C17—H17A	109.5
C16—N11—H01	124 (6)	C12—C17—H17B	109.5
C12—N11—H01	112 (6)	H17A—C17—H17B	109.5
N11—C12—C13	116.5 (6)	C12—C17—H17C	109.5
N11—C12—C17	118.8 (7)	H17A—C17—H17C	109.5
C13—C12—C17	124.6 (7)	H17B—C17—H17C	109.5
C12—C13—C14	121.5 (6)	C14—C18—H18A	109.5
C12—C13—H13	119.2	C14—C18—H18B	109.5
C14—C13—H13	119.2	H18A—C18—H18B	109.5
C15—C14—C13	117.9 (7)	C14—C18—H18C	109.5
C15—C14—C18	121.2 (7)	H18A—C18—H18C	109.5
C13—C14—C18	120.8 (6)	H18B—C18—H18C	109.5
			
C16—N11—C12—C13	0.7 (10)	C12—C13—C14—C18	−177.5 (6)
C16—N11—C12—C17	−176.6 (7)	C13—C14—C15—C16	−1.3 (11)
N11—C12—C13—C14	−1.1 (9)	C18—C14—C15—C16	177.6 (7)
C17—C12—C13—C14	176.1 (7)	C12—N11—C16—C15	−0.7 (12)
C12—C13—C14—C15	1.4 (10)	C14—C15—C16—N11	1.0 (11)

### 2,4-Dimethylpyridinium tetrabromidoaurate(III) (7) Hydrogen-bond geometry (Å, º)

**Table:**

*D*—H···*A*	*D*—H	H···*A*	*D*···*A*	*D*—H···*A*
N11—H01···Br4	0.82 (8)	2.82 (7)	3.435 (7)	134 (7)
N11—H01···Br1	0.82 (8)	2.92 (8)	3.527 (6)	133 (7)
C15—H15···Br2^i^	0.95	2.96	3.622 (7)	128
C18—H18*B*···Br2^ii^	0.98	2.94	3.780 (8)	145
C16—H16···Br3^i^	0.95	3.03	3.981 (7)	177
C18—H18*A*···Br3^iii^	0.98	2.93	3.903 (7)	174
C17—H17*A*···Br4^iv^	0.98	3.01	3.862 (8)	146

Symmetry codes: (i) *x*−1/2, −*y*+3/2, −*z*+1; (ii) −*x*+1/2, −*y*+2, *z*−1/2; (iii) −*x*+1, *y*−1/2, −*z*+1/2; (iv) −*x*+1, *y*+1/2, −*z*+1/2.
